# Complete Mitochondrial Genome of *Melophagus ovinus* from Qinghai-Tibet Plateau Provides Evidence for D-Loop Length Polymorphism

**DOI:** 10.3390/genes17060689

**Published:** 2026-06-11

**Authors:** Leyi Li, Huiling Xie, Zhibing Li, Wenqiang Tang, Chunxia Zhang, Xiaoxia Qi, Runbo Luo, Wenting Chui, Jun Kui, Fuqiang Huang

**Affiliations:** 1 School of Animal Science and Technology, Foshan University, Foshan 528225, China; le13822259727@163.com (L.L.); alice992036@outlook.com (H.X.); 18194008980@163.com (Z.L.); 2 Institute of Animal Science, Tibet Academy of Agriculture and Animal Husbandry Sciences, Lhasa 850009, China; 3 Department of Animal Medicine, Qinghai Agri-Animal Husbandry Vocational College, Xining 812100, China; 4 Animal Husbandry and Veterinary Station of Menyuan, Haibei Tibetan Autonomous Prefecture 810300, China; 5 Key Laboratory for Prevention and Control of Hydatid Disease in Xizang (Co-Constructed by Ministry and Province), Ministry of Agriculture and Rural Affairs, College of Animal Science, Xizang Agricultural and Animal Husbandry University, Nyingchi 860000, China; 6 Animal Disease Prevention and Control Center of Qinghai Province, Xining 810000, China; 7 Xining Animal Disease Prevention and Control Center, Xining 810000, China

**Keywords:** *Melophagus ovinus*, mitochondrial genome, D-loop, length polymorphism, nanopore sequencing

## Abstract

**Background/Objectives**: *Melophagus ovinus* is an economically important ectoparasite of small ruminants with a broad global distribution. Although mitochondrial genomes are widely used in population genetic studies, the D-loop region of *M. ovinus* remains poorly characterized because its high AT content and repetitive structure complicate amplification, assembly, and sequencing. **Methods**: We sequenced the mitochondrial genome of *M. ovinus* collected from Qinghai using an integrative approach combining Illumina paired-end sequencing, targeted PCR amplification, and Nanopore long-read sequencing. Comparative genomic analysis was performed against published mitogenomes from Gansu (MH024396) and Xinjiang (NC_037368). **Results**: The Qinghai mitochondrial genome contained the typical 37 mitochondrial genes within a 14,728 bp conserved region. Comparative analysis revealed exceptionally high conservation (>99.6% sequence identity) among Qinghai, Gansu, and Xinjiang isolates outside the D-loop region. Notably, the D-loop exhibited length polymorphism, with different assembly strategies or samples yielding lengths ranging from 317 bp to 2385 bp. Targeted long-read sequencing of ten individuals identified a predominant D-loop variant of approximately 844 bp in nine samples and a markedly shorter variant of approximately 164 bp in one sample. The short variant was characterized by extensive deletions and a novel 45 bp insertion. Support for this variant was obtained from independent Illumina DNA-seq, RNA-seq, Nanopore sequencing, and de novo assembly analyses. **Conclusions**: This study provides preliminary evidence for D-loop structural heterogeneity in *M. ovinus*, suggesting remarkable length polymorphism and complex indel patterns that require further validation. These findings significantly expand the genomic resources available for this important veterinary parasite and establish a foundation for future population genetic and evolutionary studies.

## 1. Introduction

The superfamily Hippoboscoidea comprises four families—Hippoboscidae, Glossinidae, Streblidae, and Nycteribiidae—of which Hippoboscidae is the most species-rich, containing more than 213 described species in 21 genera [1]. Among them, the sheep ked, *Melophagus ovinus* (Diptera: Hippoboscidae), is one of the most economically important ectoparasites of small ruminants worldwide [2,3].

*M. ovinus* is a wingless, permanent ectoparasite that completes its entire life cycle on a single host, with transmission occurring primarily through direct animal-to-animal contact [3,4]. The species reproduces via adenotrophic viviparity, a specialized reproductive strategy characteristic of hippoboscoid flies, in which females retain and nourish a single larva internally until it is deposited as a mature, ready-to-pupate larva [5]. Although its host range is largely restricted to sheep and goats [4], occasional infestations have been reported in red foxes, rabbits, and European bison [1]. The species is widely distributed across Europe, Asia, North America, Africa, and Oceania [4], and is particularly prevalent in western China, where infestations have been documented in Xinjiang [6,7,8,9,10,11,12], Qinghai [4,13], Gansu [14], and Xizang [15].

On the Qinghai–Tibet Plateau, infestations of *M. ovinus* pose a persistent threat to livestock health and productivity. Affected animals suffer intense pruritus, leading to excessive scratching, rubbing, and biting that exacerbate skin damage [2]. Heavy infestations cause anemia, impaired weight gain, and reduced wool quality and yield, while severe cases may be complicated by secondary bacterial infections and myiasis, collectively resulting in substantial economic losses to the livestock industry [2,12,16]. In addition, *M. ovinus* has been implicated as a potential vector for a diverse array of zoonotic and veterinary pathogens, including *Bartonella* spp., *Anaplasma* spp., *Trypanosoma* spp., *Rickettsia* spp., *Borrelia burgdorferi*, *Bluetongue virus*, *Border disease virus*, and *Theileria* spp. [1,2,3,7,8,9,10,11,12,13,16,17,18,19]. Recent genomic and metagenomic investigations have further highlighted the remarkable diversity of microorganisms associated with *M. ovinus*, reinforcing its importance as a potential reservoir and vector of veterinary and zoonotic pathogens [4,12].

Mitochondrial genomes have become indispensable tools in phylogenetic inference, species delimitation, and population genetic studies, owing to their relatively conserved gene content, small size, maternal inheritance, and faster evolutionary rate compared to nuclear DNA [20]. As of April 2026, only a limited number of complete mitochondrial genomes are available for Hippoboscoidea, including two published mitogenomes of *M. ovinus* from Xinjiang (NC_037368) and Gansu (MH024396). Although these resources provide valuable genomic references, little is known about mitochondrial variation among geographically distinct populations of *M. ovinus*.

The mitochondrial control region, also known as the displacement loop (D-loop) or AT-rich region, plays a central role in mitochondrial replication and transcription and is typically the most variable region of insect mitochondrial genomes [21,22]. Owing to its high A + T content, tandem repeats, and potential secondary structures, this region frequently exhibits substantial length variation and remains difficult to amplify and assemble accurately using conventional PCR and short-read sequencing approaches [14,23,24]. Consequently, D-loop sequences are often incompletely resolved or excluded from comparative mitogenomic analyses [6,14]. Recent advances in long-read sequencing technologies, particularly Oxford Nanopore sequencing, have substantially improved the characterization of repetitive and structurally complex mitochondrial regions. A recent study demonstrated the utility of Nanopore adaptive sampling for targeted mitochondrial genome sequencing and bloodmeal identification in hematophagous insects, highlighting its potential for resolving challenging mitochondrial regions [25]. However, these approaches have not yet been applied to investigate D-loop variation in *M. ovinus* [23,26].

In the present study, we sequenced and annotated the mitochondrial genome of *M. ovinus* collected from Qinghai Province, China, and compared it with published mitogenomes from Xinjiang and Gansu. Particular emphasis was placed on characterizing the structurally complex D-loop region using an integrated strategy combining Illumina sequencing, two-step PCR amplification, Sanger sequencing, and Nanopore long-read sequencing. This work expands the mitochondrial genomic resources available for *M. ovinus* and provides evidence for structural variation within its mitochondrial control region.

## 2. Materials and Methods

### 2.1. Specimen Collection and Morphological Identification

Adult specimens of *M. ovinus* were collected from naturally infested Tibetan sheep in Huangyuan County, Qinghai Province, China. A total of ten individuals were obtained during a single collection event and used for subsequent analyses. Specimens were preserved in 75% ethanol and transported to the laboratory for processing. Morphological identification was performed as described previously [27].

### 2.2. DNA Extraction and Next Generation Sequencing

Total genomic DNA was extracted from individual keds using the FastPure Blood/Cell/Tissue/Bacteria DNA Isolation Mini Kit (Vazyme Biotech, Cat. DC112-01, Nanjing, China) following the manufacturer’s protocol. For mitogenome sequencing, DNA from a single individual was used to construct a whole-genome shotgun library, which was sequenced on an Illumina NovaSeq platform (2 × 150 bp paired-end reads). Raw reads were quality-controlled using FastQC (v0.11.9) (http://www.bioinformatics.babraham.ac.uk/projects/fastqc/ (accessed on 18 June 2024)), and adapters and low-quality bases were trimmed using AdapterRemoval v2 [28].

### 2.3. Mitogenome Assembly and Annotation

Illumina sequencing was performed by Shanghai Personal Biotechnology Co., Ltd. (Shanghai, China) on the NovaSeq platform, yielding 22,271,512 raw reads. After quality filtering (Q20: 96.20%, Q30: 90.87%), 21,052,646 clean reads were retained for assembly. Two independent workflows were employed for de novo assembly and annotation. In the first workflow, clean reads were assembled using SPAdes v3.9.0 [29] and the resulting contigs were annotated with MITOS [30]. In the second workflow, assembly and annotation were performed simultaneously using MitoZ v3.6 [31] under default parameters. Preliminary annotations from both pipelines were manually curated in Geneious Prime^®^ 2025.0.2 to resolve gene boundaries, start/stop codons, and tRNA anticodons. The final curated mitogenome was compared against two published references: MH024396 (Gansu isolate) and NC_037368 (Xinjiang isolate). The latter was assembled from Sanger sequencing of 11 overlapping amplicons following the protocol described by Wen et al. [32] (as referenced in [6]), in which PCR products that could not be directly sequenced were resolved by cloning. This assembly served as the primary reference for comparative analysis.

### 2.4. D-Loop Amplification and Long-Read Sequencing

The mitochondrial D-loop region, located between *rrnS* and *trnM*, was targeted for specialized amplification due to its high AT content and repetitive architecture, which preclude reliable assembly from short-read data [22,26,33]. Primers were designed using Primer Premier 6.25 (https://www.premierbiosoft.com/primerdesign/ (accessed on 23 October 2024)) and evaluated with Oligo 7 [34]. The primer pair was Dloop-AF (5’-GTT TAA CCG CGA TTG CTG-3’) and Dloop-AR (5’-ATG GGG TAT GAA CCC ACT-3’). PCR amplification was performed using a two-step protocol that merged annealing and extension: initial denaturation at 98 °C for 90 s; 35 cycles of 98 °C for 30 s and 55 °C for 3 min; final extension at 55 °C for 7 min. Each sample was subjected to two independent PCR amplifications. Amplification products were subjected to Sanger sequencing (Sangon Biotech, Shanghai, China) and Nanopore long-read sequencing (Jiangsu CoWin Biotech, Taizhou, China) to resolve complete D-loop sequences and assess length polymorphism. For Nanopore sequencing, libraries were constructed from 200 fmol of each PCR product using the Nanopore Sequencing DNA Ligation Library Construction Kit (CW3601, CoWin, Taizhou, China) and the Native Barcoding Kit 96 V14 (SQK-NBD114.96, Oxford Nanopore Technologies, Oxford, UK). Sequencing was performed on a PromethION 2 Solo instrument with R10.4.1 flow cells (FLO-PRO114M, Oxford Nanopore Technologies, Oxford, UK). Basecalling was conducted using the super-accuracy model (sup v4.3.0). Raw reads were quality-filtered with Fastplong v0.2.2 [35] to remove low-quality and short sequences, as well as barcode self-ligation artifacts. The resulting clean reads were assembled de novo to generate preliminary PCR consensus sequences, which were further polished with Medaka v2.0.1 (https://github.com/nanoporetech/medaka (accessed on 14 April 2026)). Clean reads were then aligned back to the polished consensus using Minimap2 v2.28 [36], and a self-developed mutation detection pipeline was applied to identify and correct residual errors, yielding the final consensus sequences.

### 2.5. Variant Detection and Comparative Analysis

Clean paired-end reads were quality-filtered using the integrated BBDuK program in Geneious Prime with a minimum base quality threshold of Q20, and mapped against the reference mitogenome NC_037368 using the Geneious Mapper implemented in Geneious Prime v2025.0.2 (Biomatters Ltd., Auckland, New Zealand) under the “Medium-low Sensitivity/Fast” setting. Single-nucleotide polymorphisms (SNPs) were identified using the “Find Variations/SNPs” function in Geneious Prime. Variant calling was performed with a minimum coverage threshold of 5× and a minimum variant frequency of 25%. To assess assembly consistency in the D-loop region, Illumina clean reads were mapped to the D-loop sequences extracted from the SPAdes and MitoZ assemblies, as well as from two published references (MH024396 and NC_037368). Read coverage was calculated using samtools depth and visualized in R. Coverage statistics, including average depth, maximum depth, minimum depth, and the percentage of positions with ≥5×, ≥10×, and ≥20× coverage, were calculated for each D-loop region. Nucleotide frequencies at each polymorphic position were calculated from all mapped reads. SNP positions and read support were subsequently inspected manually and visualized using Integrative Genomics Viewer (IGV) v2.15 [37] to verify the consistency of the detected variants. Pairwise sequence identities and genetic distances among Qinghai, Xinjiang, and Gansu isolates were calculated in Geneious Prime after excluding the D-loop region. To validate the 45 bp insertion identified in the short D-loop variant, clean reads from Illumina DNA-seq, RNA-seq (SRA: SRR17267914), and Nanopore sequencing were aligned to the 415 bp D-loop sequence.

### 2.6. Survey of Cox1 Start Codon Usage

To contextualize the annotation discrepancy observed in *M. ovinus*, we surveyed *cox1* start codon usage across 186 mitochondrial genomes from Calyptratae available in the NCBI RefSeq database and 29 Hippoboscoidea mitogenomes from the GenBank database. Start codon information was extracted from the annotated CDS features of each genome. Accessions and corresponding start codons are listed in Supplementary Table S4.

## 3. Results

### 3.1. Comparative Genomic Features of M. ovinus Mitogenomes

All specimens were identified as *M. ovinus* based on morphological examination (Supplementary Figure S1). The mitogenomes of *M. ovinus* QH assembled using SPAdes v3.9.0 and MitoZ v3.6 were 17,113 bp and 15,603 bp in length, respectively. Hereafter, the SPAdes-derived assembly is referred to as *M. ovinus* QHs (Qinghai–SPAdes assembly), while the MitoZ-derived assembly is referred to as *M. ovinus QHm* (Qinghai–MitoZ assembly). The discrepancy between these assemblies was confined entirely to the D-loop region (2385 bp in QHs versus 845 bp in QHm), whereas the remaining regions were identical (Supplementary Figure S2). This variation reflects the inherent difficulty of assembling AT-rich, repetitive regions from short-read data. Consequently, the D-loop was excluded from subsequent comparative analyses, and a conserved region of 14,728 bp (excluding the D-loop) was used for population comparisons. Both assemblies contained the typical 37 mitochondrial genes: 13 protein-coding genes (PCGs), 2 rRNA genes, 22 tRNA genes, and one D-loop, with 23 genes encoded on the J-strand. Among the PCGs, eleven utilized standard ATN start codons, whereas *cox1* initiated with TCG and *nad1* with TTG (Table 1).

**Figure 1:**
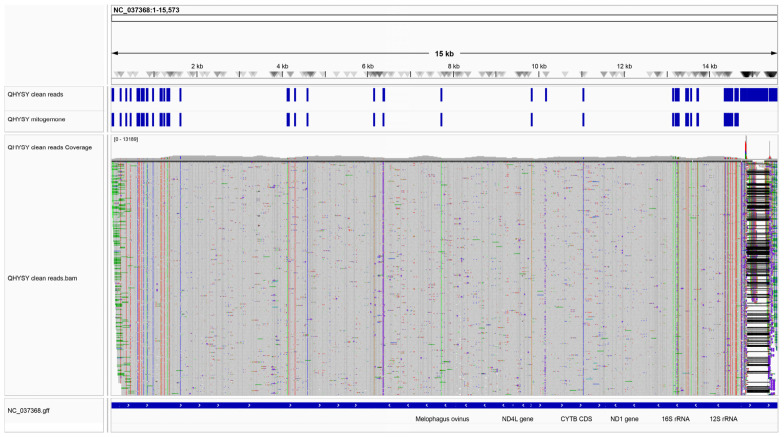
Variant calling from Illumina DNA-seq data mapped to the *M. ovinus* reference mitogenome (NC_037368). Blue bars indicate variant sites identified in Geneious Prime. The upper panel shows variants detected by mapping Illumina clean reads to the reference mitogenome, whereas the lower panel shows variants identified by comparing the de novo assembled Qinghai mitogenome (excluding the D-loop region) with the reference sequence. The black line within the D-loop region indicates a deletion signal detected in the read-mapping analysis.

The lengths of the assembled *M. ovinus* mitogenomes excluding the D-loop were 14,728 bp for QH, 14,727 bp for GS, and 14,728 bp for XJ. Sequence alignment revealed a high degree of conservation across these mitogenomes outside the D-loop, with an overall pairwise identity of 99.63%. Notably, the sequence identity between the QH and GS assemblies was 99.99% (Supplementary Table S1). The accuracy of our de novo assembly (QH) was further validated through variant analysis against the reference XJ mitogenome (NC_037368). Mapping clean reads to the reference identified a total of 55 polymorphic sites within the conserved non-D-loop regions. Among these, 51 sites (comprising single-nucleotide polymorphisms and short indels) were concordantly identified as differences between the de novo assembled QH sequence and the reference. The remaining four variant calls (one deletion and three insertions) were specific to the read-mapping analysis and were not supported by the assembly-based comparison (Figure 1, Supplementary Table S2; Supplementary Figure S2). Comparative analysis of the identified variants revealed that a subset of these mutations resulted in amino acid alterations within the protein-coding genes. Specifically, nonsynonymous substitutions were found in the *nad2*, *atp6*, *nad6*, and *cytb* genes. Notably, three amino acid changes occurred in the *atp6* gene (Supplementary Table S2).

### 3.2. Structural Characterization of the D-Loop Region

To assess whether the assembly discrepancies between SPAdes and MitoZ were caused by insufficient sequencing depth, Illumina clean reads were mapped to the D-loop regions of all four assemblies. Mean coverage ranged from 1119.7× to 2566.5×, and more than 98.5% of positions were covered at ≥20× depth (Table 2, Supplementary Figure S3).

**Figure 2:**
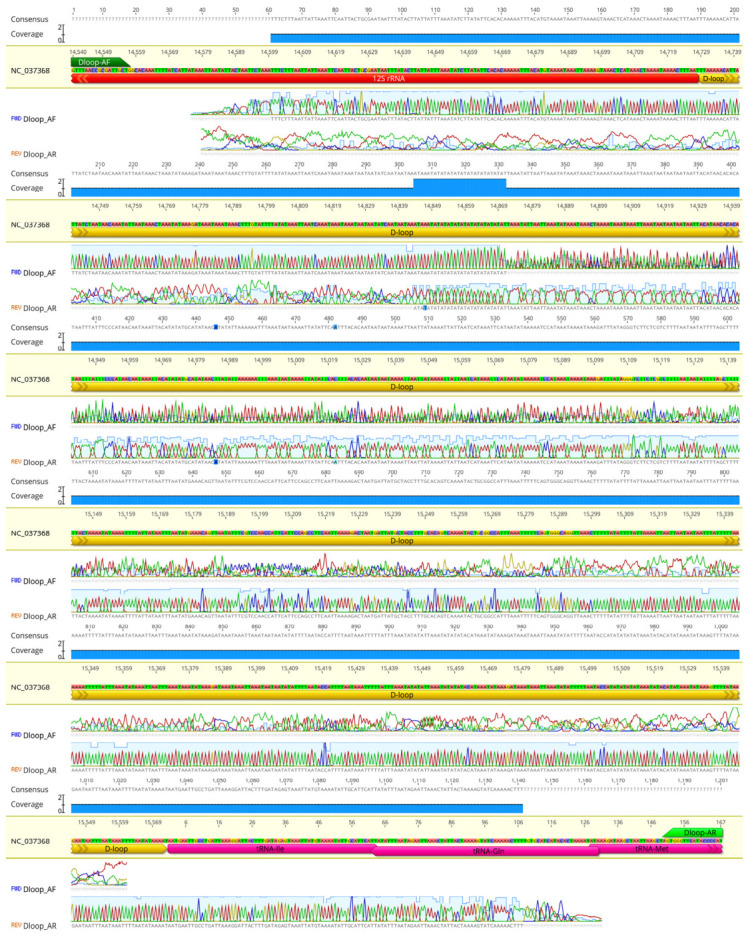
Sanger sequencing of the *M. ovinus* D-loop region. Alignment of representative forward and reverse reads to the *rrnS*–*trnM* region of the reference mitogenome (NC_037368). The forward read yielded 272 bp of high-quality sequence, whereas the reverse read yielded 837 bp. Both chromatograms showed signal deterioration downstream of a 12 bp AT-repeat tract (positions 14848–14871).

**Figure 3:**
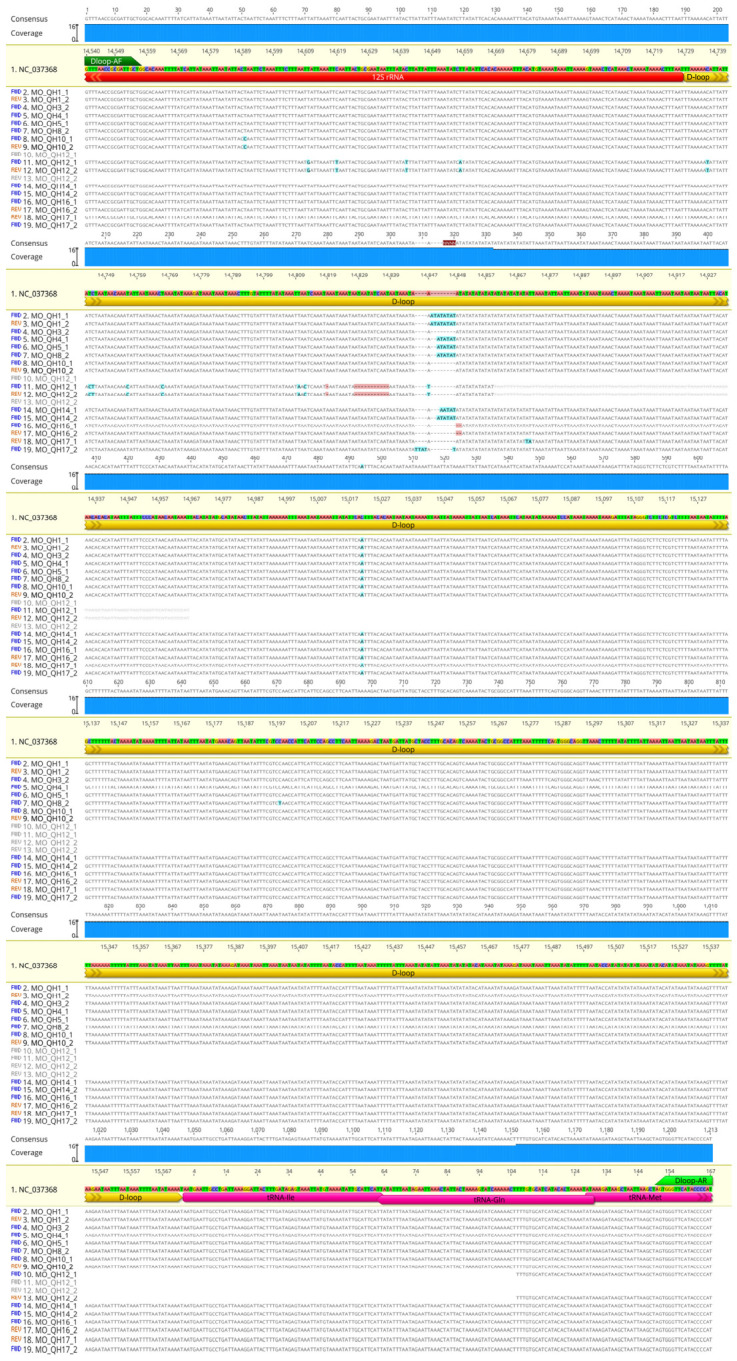
D-loop length variants identified by Nanopore sequencing. Alignment of long-read sequences to the *rrnS*-*trnM* region of NC_037368. Nine samples yielded ~1200 bp D-loop sequences, whereas one sample contained a shortened 415 bp variant with a large deletion and a 45 bp insertion.

To validate the D-loop structure, the region between *rrnS* and *trnM* was amplified from ten individuals and sequenced using Sanger and Nanopore platforms. Sanger sequencing consistently produced longer reverse reads, whereas forward reads were shorter or frequently failed (Figure 2, Supplementary Figure S4). Nanopore sequencing generated complete D-loop sequences, revealing amplicons of approximately 1200 bp (1199–1209 bp; including 189 bp *rrnS* and 167 bp tRNA flanking regions) in nine individuals and a markedly shorter 415 bp variant in one individual (including 189 bp *rrnS* and 62 bp tRNA flanking regions) (Figure 3). Although only ten individuals were analyzed, and the short D-loop variant was detected in a single specimen, this finding represents the first report of extreme length polymorphism in the *M. ovinus* D-loop. The short variant was supported by 1145 Nanopore reads, with read lengths predominantly distributed between 380 and 460 bp and a modal length of 415 bp (389 reads, 34.0%; Table S3).

**Figure 4:**
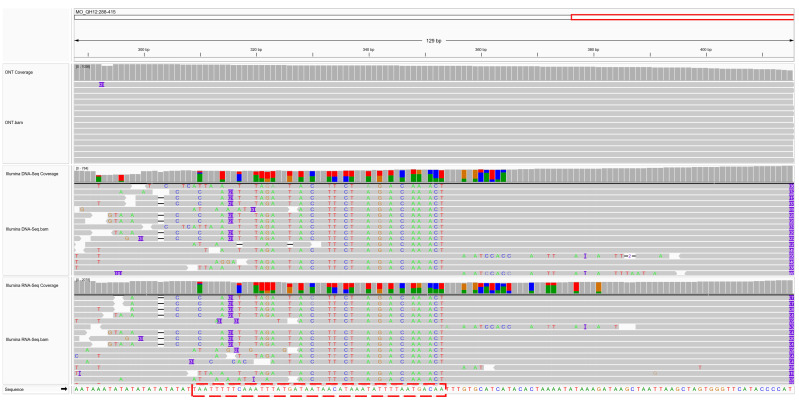
Validation of the 45 bp insertion by independent sequencing datasets. Illumina DNA-seq, RNA-seq (SRR17267914), and Nanopore reads mapped to the 415 bp D-loop sequence. The red dashed box denotes the 45 bp insertion (positions 309–353), which was detected across all three datasets.

Relative to NC_037368, the short variant contained a large deletion (positions 14860–15573 and 1–105) and a novel 45 bp insertion between positions 309 and 353 (Figure 3 and Figure 4). Deletion signals within the D-loop region were also independently detected by Illumina read mapping (Figure 1). Furthermore, the 45 bp insertion was supported by Illumina DNA-seq, RNA-seq, and Nanopore sequencing data (Figure 4). Notably, an identical 45 bp sequence was recovered in the D-loop region (positions 14858–14902) of the mitochondrial genome assembled de novo using MitoZ, and the same sequence aligned to positions 14903–14939 of the SPAdes assembly. The recovery of this insertion by two independent assembly strategies and multiple sequencing datasets supports its mitochondrial origin and reduces the likelihood that it resulted from sequencing artifacts or contamination by nuclear mitochondrial DNA segments (NUMTs).

### 3.3. Cox1 Start Codon Usage Across Calyptratae

Among 186 Calyptratae mitochondrial genomes from the RefSeq database, none used the standard ATG start codon; TCG was predominant (77.4%, 144/186), followed by CAA (14.0%, 26/186), CGA (5.4%, 10/186), ATT (2.7%, 5/186), and GTT (0.5%, 1/186). In contrast, 29 Hippoboscoidea mitochondrial genomes from GenBank exhibited greater diversity: CAA and CGA each accounted for 27.6% (8/29), AAA for 24.1% (7/29), TCG for 17.2% (5/29), and CAG for 3.4% (1/29) (Supplementary Table S4).

## 4. Discussion

The mitochondrial genome of *M. ovinus* from Qinghai falls within the expected size range for Diptera. Comparative analysis of three geographically distinct populations (Qinghai, Gansu, and Xinjiang) revealed exceptional conservation outside the D-loop region (>99.6% identity), with only 51 concordant polymorphic sites identified across the conserved regions. These findings suggest limited mitochondrial differentiation among *M. ovinus* populations in western China, although broader sampling will be necessary to confirm this pattern.

An important finding concerns the annotation of the *cox1* start codon. The two previously published *M. ovinus* mitogenomes assigned different initiation codons (TCG [6] and AAA [14]) to identical *cox1* sequences. Our comparative survey revealed that TCG was the predominant *cox1* start codon in curated Calyptratae RefSeq mitogenomes (77.4%), whereas considerable annotation variability was observed among Hippoboscoidea entries. Because identical *cox1* sequences can receive different start codon annotations, much of this variation likely reflects annotation inconsistencies rather than genuine biological differences. These findings highlight the need for standardized annotation practices in mitochondrial genome studies, particularly for genes with non-canonical initiation sites.

Most PCGs terminated with complete TAA or TAG stop codons, whereas *cox2*, *cox3*, and *nad5* possessed incomplete stop codons (T-), a common feature in insect mitochondrial genomes that is corrected through post-transcriptional polyadenylation [38,39,40]. Several nonsynonymous substitutions were detected in *nad2*, *atp6*, *nad6*, and *cytb*, including three amino acid changes in *atp6*. Because these genes encode components of the mitochondrial oxidative phosphorylation pathway, the observed substitutions may potentially influence mitochondrial function. However, their biological significance remains unclear and warrants further investigation.

Accurately characterizing the mitochondrial D-loop remains a persistent technical challenge due to its high AT-content and repetitive structure [14,24]. The D-loop region exhibited substantial length polymorphism. Long-read sequencing revealed D-loop sequences of approximately 844 bp in nine individuals and a markedly shorter variant (~164 bp) in one individual. The short variant contained extensive deletions and a novel 45 bp insertion. Independent support for this shortened haplotype was obtained from Illumina read mapping, which identified deletion signals within the D-loop region. The insertion sequence was also recovered by two independent assembly strategies and supported by Illumina DNA-seq, RNA-seq, and Nanopore sequencing data, reducing the likelihood that it resulted from sequencing artifacts or NUMT contamination. Although the functional significance of this structural variation remains unclear, such extensive indel polymorphism may complicate the use of the D-loop as a marker for population genetic analyses.

Several limitations should be acknowledged. Firstly, only ten individuals from a single locality were examined, and the short D-loop variant was detected in a single specimen, limiting the generalizability of the findings. Secondly, although multiple independent datasets support the authenticity of the short variant, alternative explanations such as heteroplasmy cannot be completely excluded. Future studies incorporating larger sample sizes, broader geographic coverage, and PCR-free mitochondrial sequencing approaches will be necessary to determine the prevalence and biological significance of this variant.

Despite these limitations, the combined use of Illumina sequencing for conserved mitochondrial regions and targeted Nanopore sequencing for the D-loop proved effective for resolving structurally complex mitochondrial regions. This integrated strategy provides a practical approach for complete mitogenome characterization in non-model organisms. Future work should expand geographic sampling and incorporate additional genetic markers to improve our understanding of the evolutionary history and population structure of *M. ovinus*.

## 5. Conclusions

This study provides preliminary evidence for D-loop structural heterogeneity in *M. ovinus* based on ten individuals from Qinghai. The main findings are:

(1) The *M. ovinus* mitogenome is highly conserved across the Qinghai, Gansu, and Xinjiang populations (>99.6% identity outside the D-loop).
(2) The D-loop region exhibits length polymorphism, including a rare short variant (~164 bp) with a novel 45 bp insertion, and large deletions were detected, which warrants further validation.
(3) Discrepancies in *cox1* start codon annotation among published *M. ovinus* mitogenomes result from annotation inconsistencies rather than biological variation, underscoring the need for standardized protocols.

These findings expand genomic resources available for *M. ovinus* and provide a foundation for future population genetic studies. Broader geographic sampling and PCR-free approaches will be necessary to validate the rare short variant and definitively characterize D-loop heterogeneity.

## Figures and Tables

**Table 1:** Comparing organization of the mitochondrial genome of *M. ovinus* from Qinghai (assembled using SPAdes and MitoZ), Xinjiang and Gansu, China.

Genes	Nucleotide					Codon		
	*M. ovinus* QHs/QHm/XJ/GS *					*M. ovinus* QH/XJ/GS *		
	Start	End	Length (bp)	Intergenic Nucleotide	Strand	Start	Stop	Anti- Codon
*trnI*	1/1/1/1	64/64/64/66	64/64/64/66	0/0/0/0	J			GAT
*trnQ*	62/62/62/62	130/130/130/132	69/69/69/71	−3/−3/−3/−5	N			TTG
*trnM*	128/128/128/129	195/195/195/196	68/68/68/68	−3/−3/−3/−4	J			CAT
*n* *ad2*	196/196/196/197	1206/1206/1206/1207	1011/1011/1011/1011	0/0/0/0	J	ATT/ATT/ATT/ATT	TAG/TAG/TAG/TAG	
*trnW*	1212/1212/1212/1213	1278/1278/1278/1279	67/67/67/67	5/5/5/5	J			TCA
*trnC*	1271/1271/1271/1272	1334/1334/1334/1335	64/64/64/64	−8/−8/−8/−8	N			GCA
*trnY*	1344/1334/1344/1344	1409/1409/1409/1409	66/66/66/66	9/9/9/8	N			GTA
*cox1*	1408/1408/1408/1411	2941/2941/2941/2941	1534/1534/1534/1531	−2/−2/−2/1	J	TCG/TCG/TCG/AAA	T--/T--/T--/T--	
*trnL2*	2042/2942/2942/2942	3006/3006/3006/3006	65/65/65/65	−5/−5/−5/−5	J			TAA
*cox2*	3011/3011/3011/3011	3695/3695/3692/3692	685/685/682/682	4/4/4/4	J	ATG/ATG/ATG/ATG	T--/T--/T--/T--	CTT
*trnK*	3693/3693/3693/3693	3763/3763/3763/3763	71/71/71/71	0/0/0/0	J			GTC
*trnD*	3765/3765/3765/3765	3830/3830/3830/3830	66/66/66/66	1/1/1/1	J			
*atp8*	3831/3831/3831/3831	3986/3986/3986/3986	156/156/156/156	0/0/0/0	J	ATT/ATT/ATT/ATT	TAA/TAA/TAA/TAA	
*atp6*	3980/3980/3980/3980	4657/4657/4657/4657	678/678/678/678	−7/−7/−7/−7	J	ATG/ATG/ATG/ATG	TAA/TAA/TAA/TAA	
*cox3*	4657/4657/4657/4657	5443/5443/5443/5443	787/787/787/787	−1/−1/−1/−1	J	ATG/ATG/ATG/ATG	T--/T--/T--/T--	
*trnG*	5444/5444/5444/5444	5505/5505/5505/5505	62/62/62/62/62	0/0/0/0	J			TCC
*nad3*	5506/5506/5506/5506	5859/5859/5859/5859	354/354/354/354	0/0/0/0	J	ATT/ATT/ATT/ATT	TAA/TAA/TAA/TAA	
*trnA*	5860/5860/5860/5860	5922/5922/5922/5922	63/63/63/63	0/0/0/0	J			TGC
*trnR*	5922/5922/5922/5922	5984/5984/5984/5984	63/63/63/63	−1/−1/−1/−1	J			TCG
*trnJ*	5986/5986/5986/5986	6049/6049/6049/6049	64/64/64/64	1/1/1/1	J			GTT
*trnS1*	6049/6049/6049/6048	6115/6115/6115/6110	67/67/67/63	−1/−1/−1/−2	J			TCT
*trnE*	6117/6117/6117/6117	6183/6183/6183/6183	67/67/67/67	1/1/1/6	J			TTC
*trnF*	6203/6203/6203/6204	6267/6267/6267/6267	65/65/65/64	19/19/19/20	N			GAA
*nad5*	6268/6268/6268/6268	7993/7993/7993/7978	1726/1726/1726/1711	0/0/0/0	N	ATT/ATT/ATT/ATT	T--/T--/T--/T--	
*trnH*	7994/7994/7994/7995	8058/8058/8058/8057	65/65/65/63	0/0/0/16	N			GTG
*nad4*	8058/8058/8058/8058	9395/9395/9395/9395	1338/1338/1338/1338	−1/−1/−1/0	N	ATG/ATG/ATG/ATG	TAA/TAA/TAA/TAA	
*nad4l*	9395/9395/9395/9395	9679/9679/9679/9679	285/285/285/285	−1/−1/−1/−1	N	ATG/ATG/ATG/ATG	TAA/TAA/TAA/TAA	
*trnT*	9682/9682/9682/9682	9744/9744/9744/9744	63/63/63/63	2/2/2/2	J			TGT
*trnP*	9745/9745/9745/9745	9811/9811/9811/9811	67/67/67/67	0/0/0/0	N			TGG
*nad6*	9813/9813/9813/9813	10,331/10,331/10,331/10,331	519/519/519/519	1/1/1/1	J	ATT/ATT/ATT/ATT	TAA/TAA/TAA/TAA	
*cytb*	10,331/10,331/10,331/10,331	11,467/11,467/11,467/11,467	1137/1137/1137	−1/−1/−1/−1	J	ATG/ATG/ATG/ATG	TAG/TAG/TAG/TAG	
*trnS2*	11,466/11,466/11,466/11,466	11,531/11,531/11,531/11,531	66/66/66/66	−2/−2/−2/−2	J			TGA
*nad1*	11,552/11,552/11,552/11,552	12,499/12,499/12,499/12,499	948/948/948/948	20/20/20/20	N	TTG/TTG/TTG/TTG	TAA/TAA/TAA/TAA	
*trnL1*	12,500/12,500/12,500/12,500	12,562/12,562/12,562/12,562	63/63/63/63	0/0/0/0	N			TAG
*rrnL*	12,563/12,563/12,563/12,563	13,878/13,878/13,878/13,878	1316/1316/1316/1316	0/0/0/0	N			
*trnV*	13,879/13,879/13,879/13,879	13,949/13,949/13,949/13,949	71/71/71/71	0/0/0/0	N			TAC
*rrnS*	13,950/13,950/13,950/13,950	14,728/14,728/14,728/14,727	779/779/779/778	0/0/0/0	N			
D-loop	14,729/14,729/14,729/14,728	17,113/15,603/15,573/15,044	2385/875/845/317	0/0/0/0	-			

* Abbreviations: QH—Qinghai; XJ—Xinjiang; GS—Gansu. The superscripts ‘QHs’ and ‘QHm’ denote de novo assemblies generated using SPAdes [29] and MitoZ [31], respectively. The mitogenome from Qinghai was sequenced and assembled in this study. The reference sequences from Xinjiang (Accession: NC_037368) and Gansu (Accession: MH024396) were retrieved from NCBI Nucleotide Databases (https://www.ncbi.nlm.nih.gov/nuccore (accessed on 12 November 2025)).

**Table 2:** Read coverage statistics for D-loop regions across four assemblies.

Assembly	D-Loop Length (bp)	Average Depth	Maximum Depth	Minimum Depth	≥5× (%)	≥10× (%)	≥20× (%)
SPAdes	2385	1246.1	16,456	38	100	100	100
MitoZ	875	1350	18,059	254	100	100	100
MH024396	317	2566.5	11,141	79	100	100	100
NC_037368	845	1119.7	11,845	2	99.64	99.29	98.58

## Data Availability

The original contributions presented in this study are included in the Article/Supplementary Materials. A total of 16 consensus sequences were deposited in GenBank under accession numbers PZ370947–PZ370962. These records correspond to the final assembled consensus sequences only. Raw Nanopore reads and Illumina sequencing data are not publicly available. Further inquiries can be directed to the corresponding author.

## Supplementary Materials

The following supporting information can be downloaded at https://www.mdpi.com/article/10.3390/genes17060689/s1. Figure S1. Morphology of *M. ovinus* under stereomicroscope. (A) Dorsal view of the female. (B) Ventral view of the female. (C) Posterior end of the female. (D) Dorsal view of the male. (E) Ventral view of the male. (F) Posterior end of the male. Co.—Coxa; Tr.—Trochanter; Fe.—Femur; Ti.—Tibia; Ta.—Tarsus; Pr.—Pretarsus; C1.—Claw. Symbols: triangle—dorsal sclerite; thick arrow—ventral sclerite; thin arrow—paramere; swallowtail arrow—pygophore. Figure S2. Comparative alignment of mitochondrial genomes from three geographic populations of *M. ovinus*. Sequence alignment of complete mitochondrial genome sequences from Qinghai (QH), Xinjiang (XJ), and Gansu (GS) isolates. The two Qinghai assemblies (QHYSY_SPAdes and QHYSY_MitoZ) are identical outside the D-loop region. Accession numbers: MH024396 (Gansu), NC_037368 (Xinjiang). QH_SPAdes and QH_MitoZ represent de novo assemblies from this study generated using SPAdes and MitoZ, respectively. Figure S3. Read coverage profiles of D-loop regions across four assemblies. (A) SPAdes (14,729–17,113 bp; 2385 bp), (B) MitoZ (14,729–15,603 bp; 875 bp), (C) MH024396 (14,728–15,044 bp; 317 bp), and (D) NC_037368 (14,729–15,573 bp; 845 bp). The lower *x*-axis indicates the mitogenome position (bp); the upper *x*-axis indicates the D-loop internal position (bp). Figure S4. Alignment of Sanger forward and reverse sequencing sequences (from several samples) to the *rrnS*-*trnM* region (spanning the D-loop) of the *M. ovinus* reference mitogenome. Alignment of forward and reverse reads from three representative samples (14, 15, 18) to the *rrnS*-*trnM* region of NC_037368. Each sample was subjected to two independent PCR amplifications with separate sequencing runs. Reverse primer sequencing consistently yielded high-quality reads, whereas forward primer sequencing produced variable results, with some replicates showing limited coverage and others failing entirely. This asymmetry reflects secondary structure interference from a 12 bp AT-repeat tract. Table S1. Genetic distances based on mitochondrial genome sequences (excluding the D-loop region) of *M. ovinus* from Xinjiang, Gansu and Qinghai. Table S2. Detection of polymorphic sites by alignment of NGS reads and the de novo assembled QH mitogenome to the reference, analyzed using Geneious Prime. Table S3. The 415 bp consensus sequence reads length distribution. Table S4. *cox1* start codon usage across 215 mitochondrial genomes from Diptera.

## Abbreviations

The following abbreviations are used in this manuscript:

QH	Qinghai
XJ	Xinjiang
GS	Gansu
D-loop	Displacement loop
NGS	Next-generation sequencing
NUMTs	Nuclear mitochondrial pseudogenes
PCGs	Protein-coding genes
