# Supplementary material for: Complete Mitochondrial Genome of Melophagus ovinus from Qinghai-Tibet Plateau Provides Evidence for D-Loop Length Polymorphism
Source: Genes (Basel). 2026 Jun 11;17(6):689. doi: 10.3390/genes17060689 (PMC13299176; doi:10.3390/genes17060689)
Supplement: Supplementary file 1 [file genes-17-00689-s001.zip › Table S1.pdf]

Table S1. Genetic distances based on mitochondrial genome sequences (excluding the D-loop region) of *Melophagus ovinus* from Xinjiang, Gansu and Qinghai

	***M. ovinus* XJ**	***M. ovinus* GS**	***M. ovinus* QHm**	***M. ovinus* QHs**
***M. ovinus* XJ**		99.63%	99.64%	99.64%
***M. ovinus* GS**	99.63%		99.99%	99.99%
***M. ovinus* QHm**	99.64%	99.99%		100%
***M. ovinus* QHs**	99.64%	99.99%	100%	

Note: Provincial abbreviations: QH, Qinghai; XJ, Xinjiang; GS, Gansu. The superscripts 'QHs' and 'OHm' denote that the corresponding genomes were assembled using the SPAdes (Bankevich et al., 2012) and MitoZ (Meng et al., 2019) software, respectively.
