# Supplementary figures and images for: Complete Mitochondrial Genome of Melophagus ovinus from Qinghai-Tibet Plateau Provides Evidence for D-Loop Length Polymorphism
Source: Genes (Basel). 2026 Jun 11;17(6):689. doi: 10.3390/genes17060689 (PMC13299176; doi:10.3390/genes17060689)

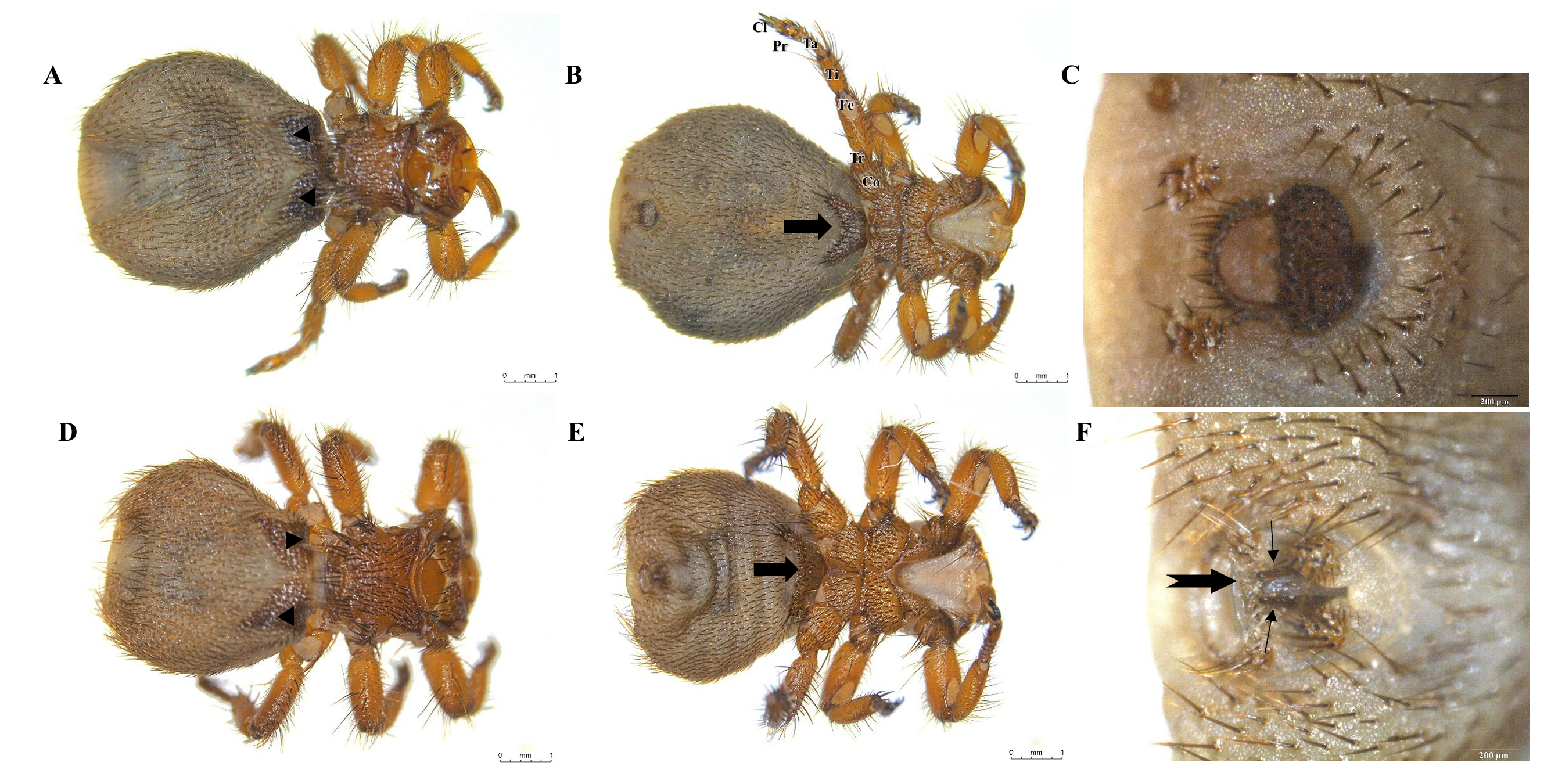

Supplement: Supplementary file 1 [file genes-17-00689-s001.zip › Figure S1.png]

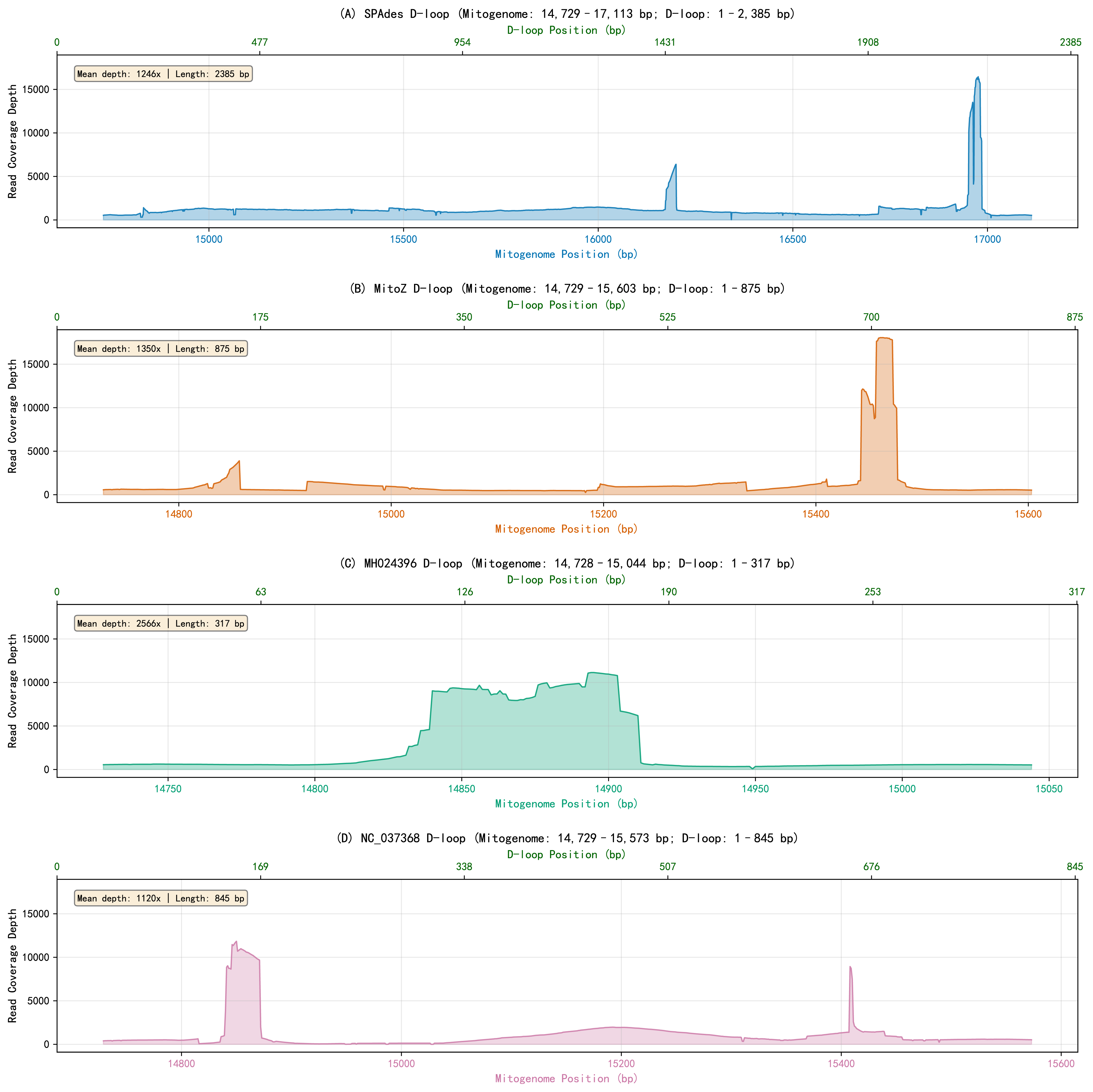

Supplement: Supplementary file 1 [file genes-17-00689-s001.zip › Figure S3.tif]

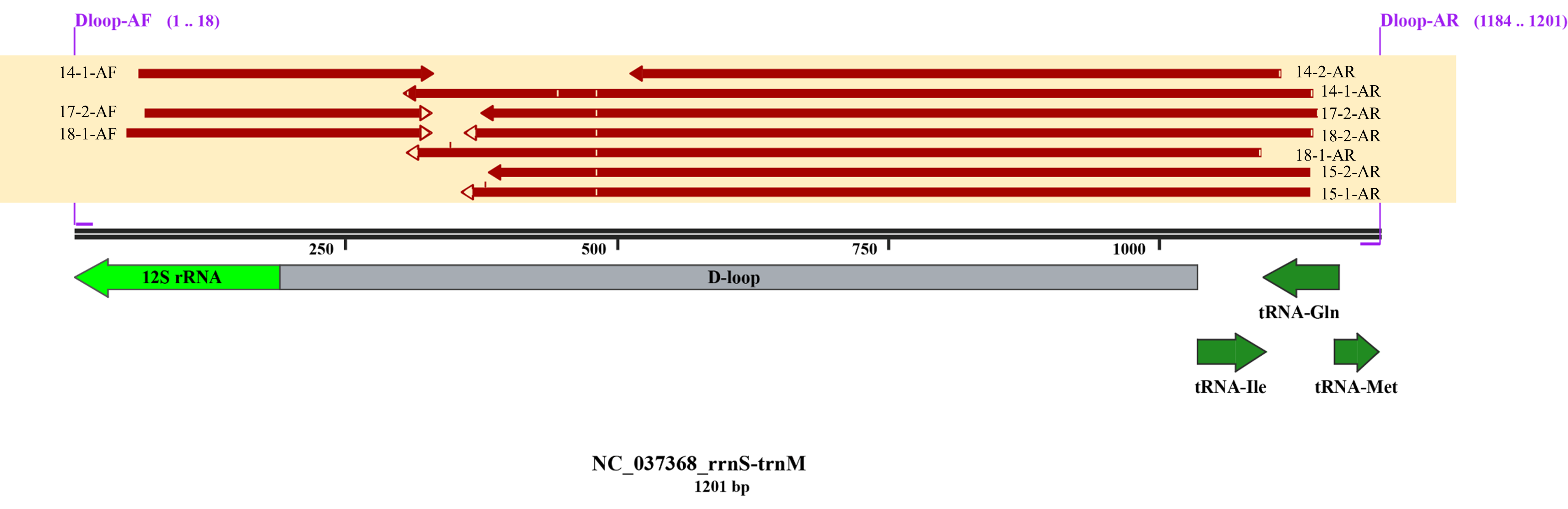

Supplement: Supplementary file 1 [file genes-17-00689-s001.zip › Figure S4.tif]

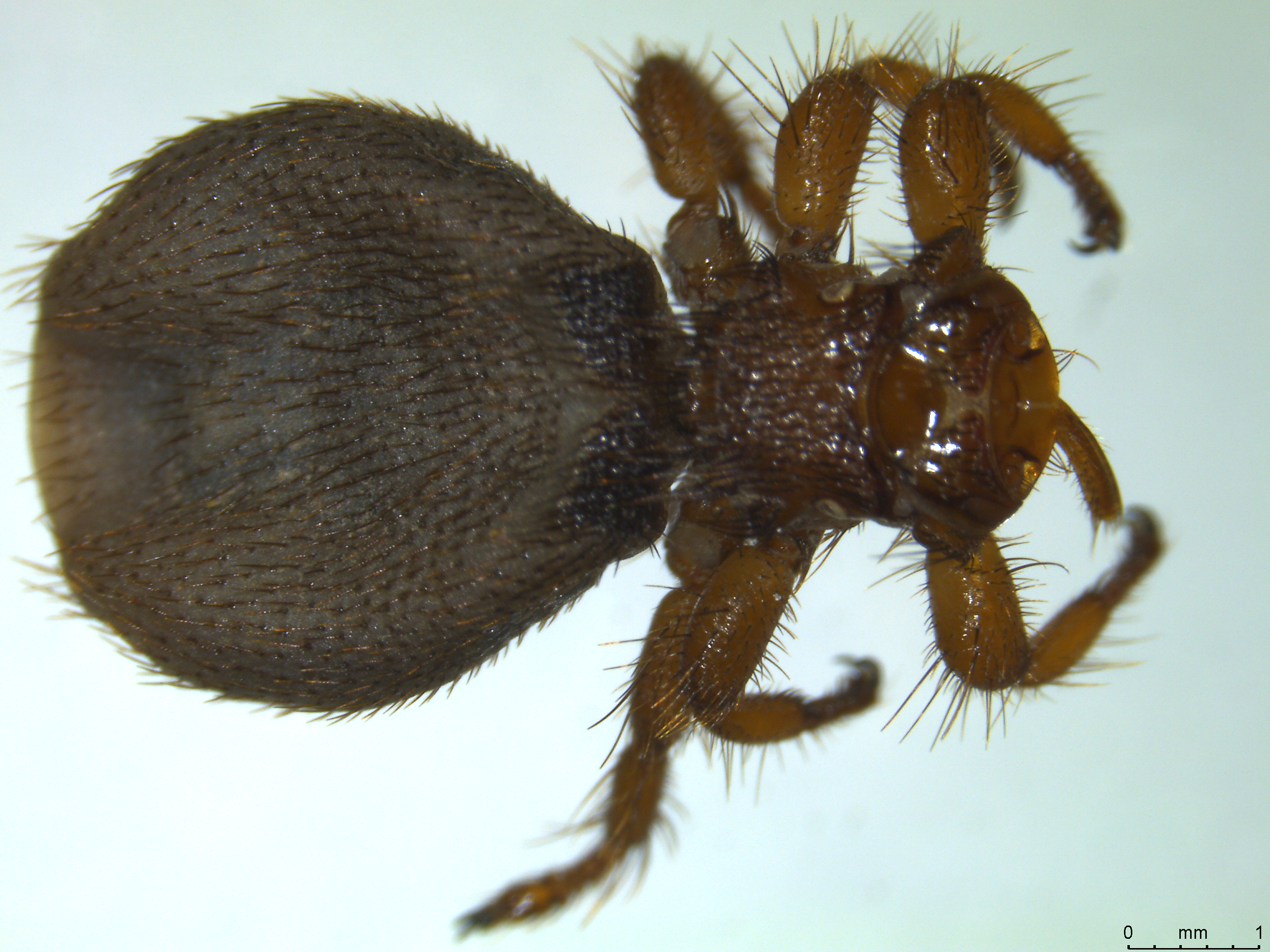

Supplement: Supplementary file 1 [file genes-17-00689-s001.zip › Original Images-Figure S1/Figure S1A.tif]

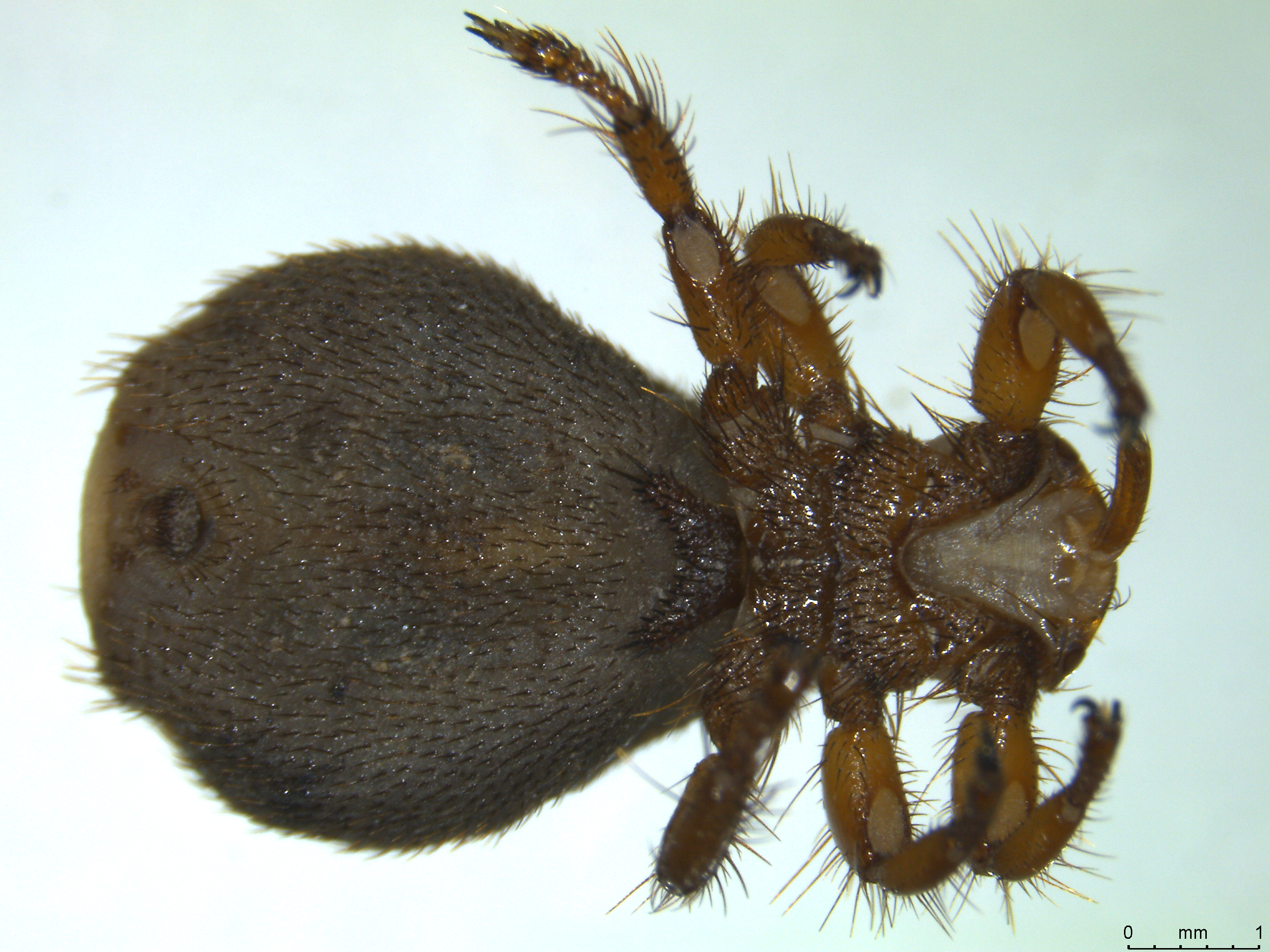

Supplement: Supplementary file 1 [file genes-17-00689-s001.zip › Original Images-Figure S1/Figure S1B.tif]

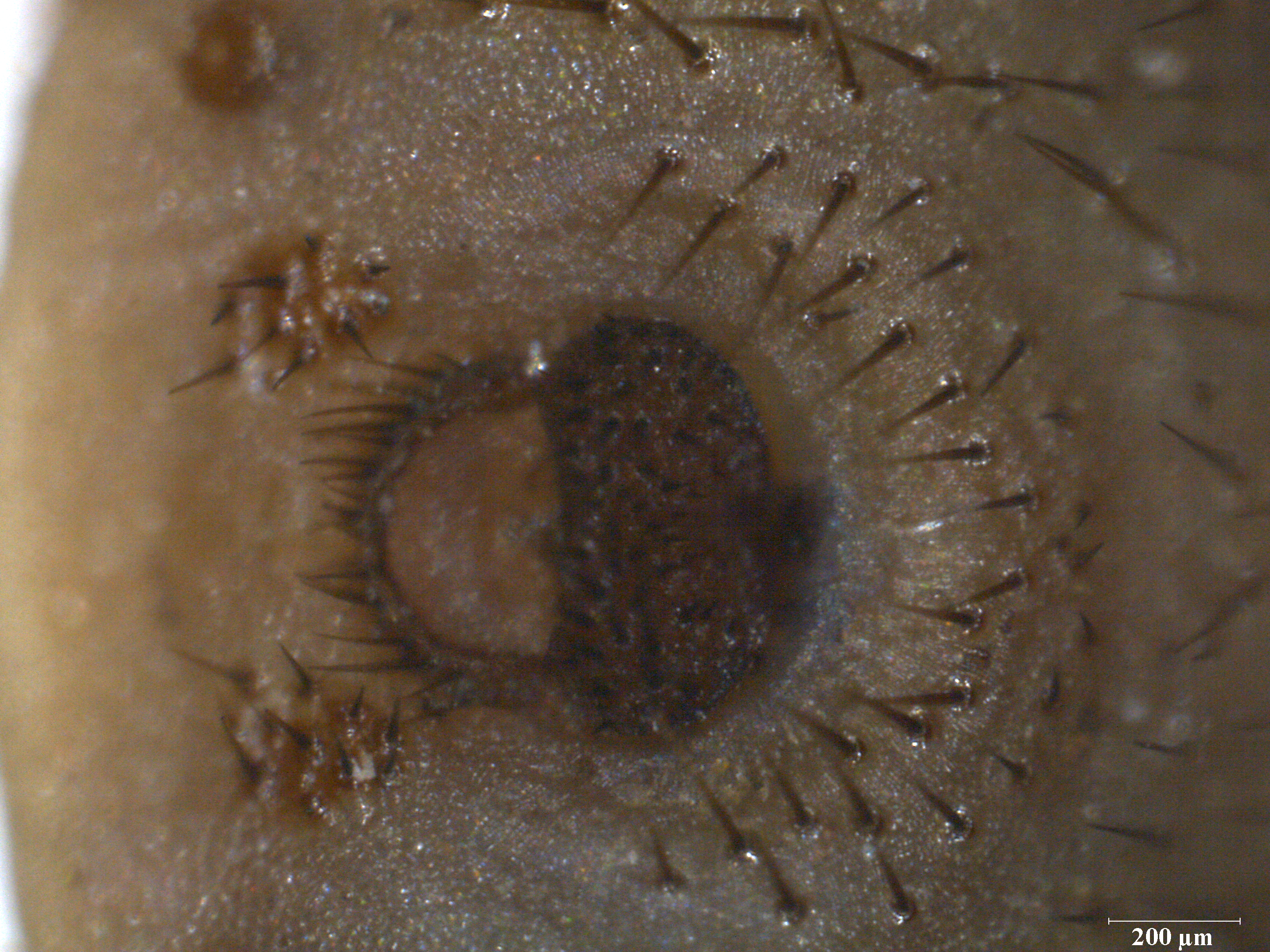

Supplement: Supplementary file 1 [file genes-17-00689-s001.zip › Original Images-Figure S1/Figure S1C.tif]

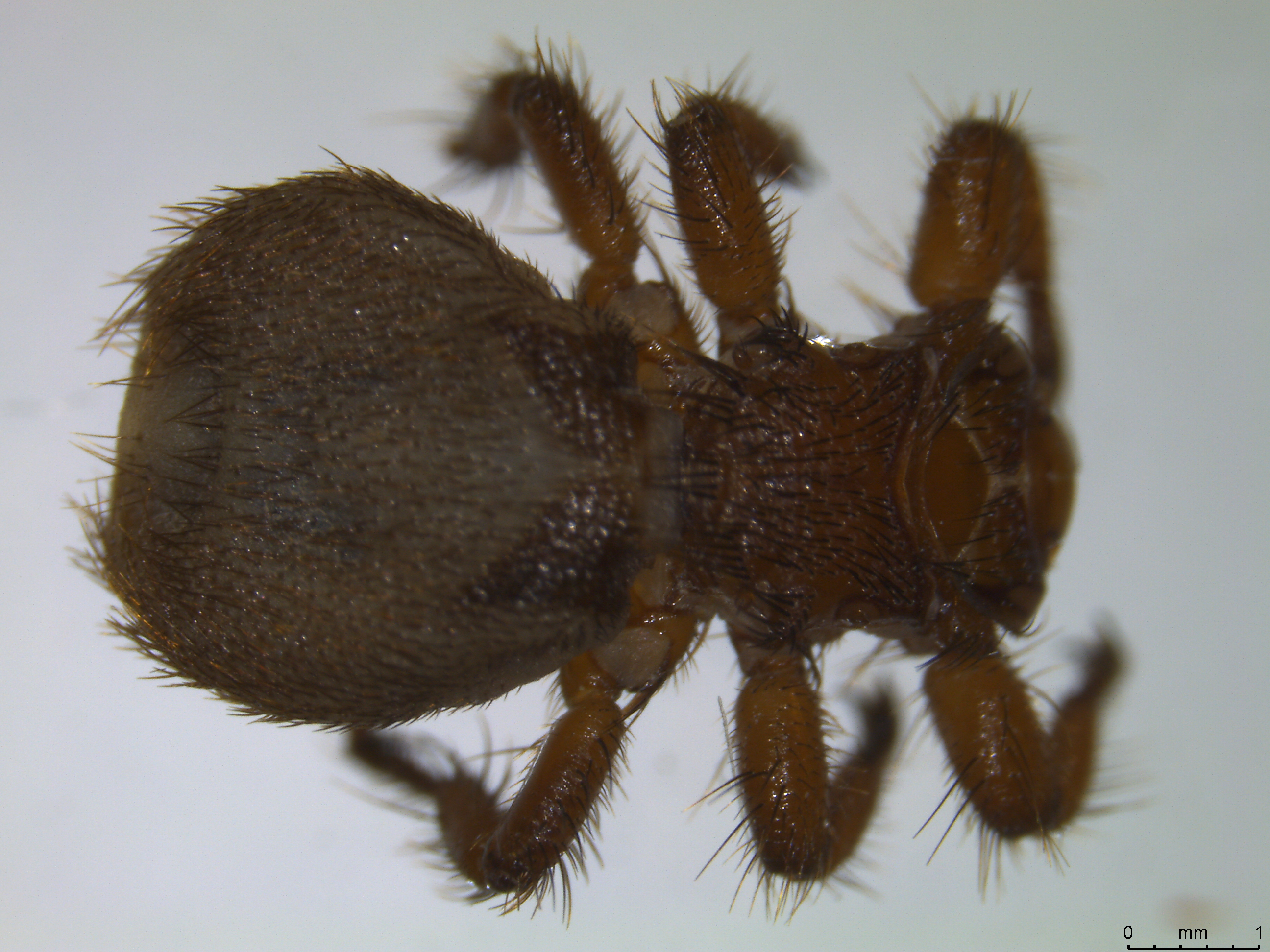

Supplement: Supplementary file 1 [file genes-17-00689-s001.zip › Original Images-Figure S1/Figure S1D.tif]

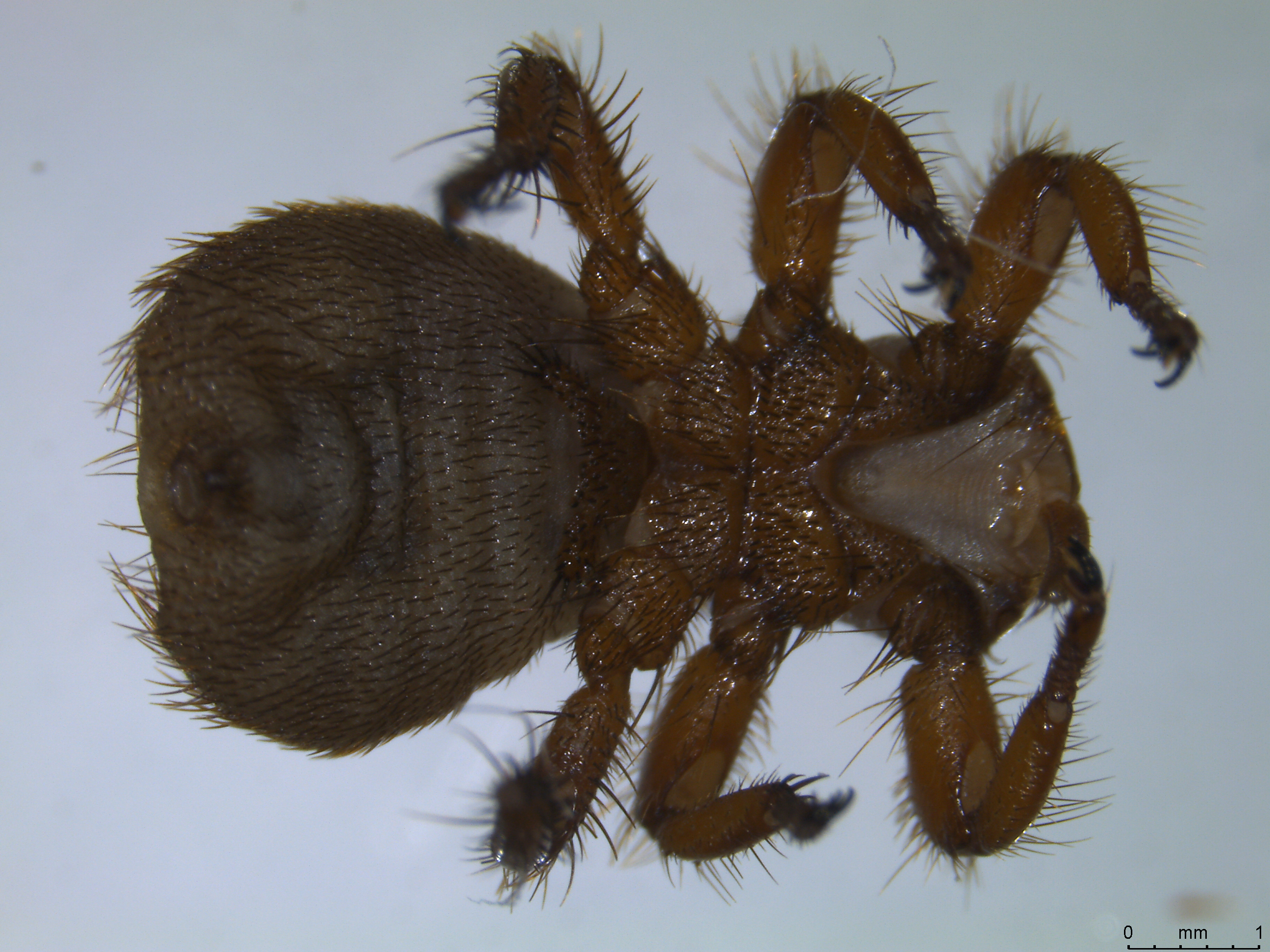

Supplement: Supplementary file 1 [file genes-17-00689-s001.zip › Original Images-Figure S1/Figure S1E.tif]

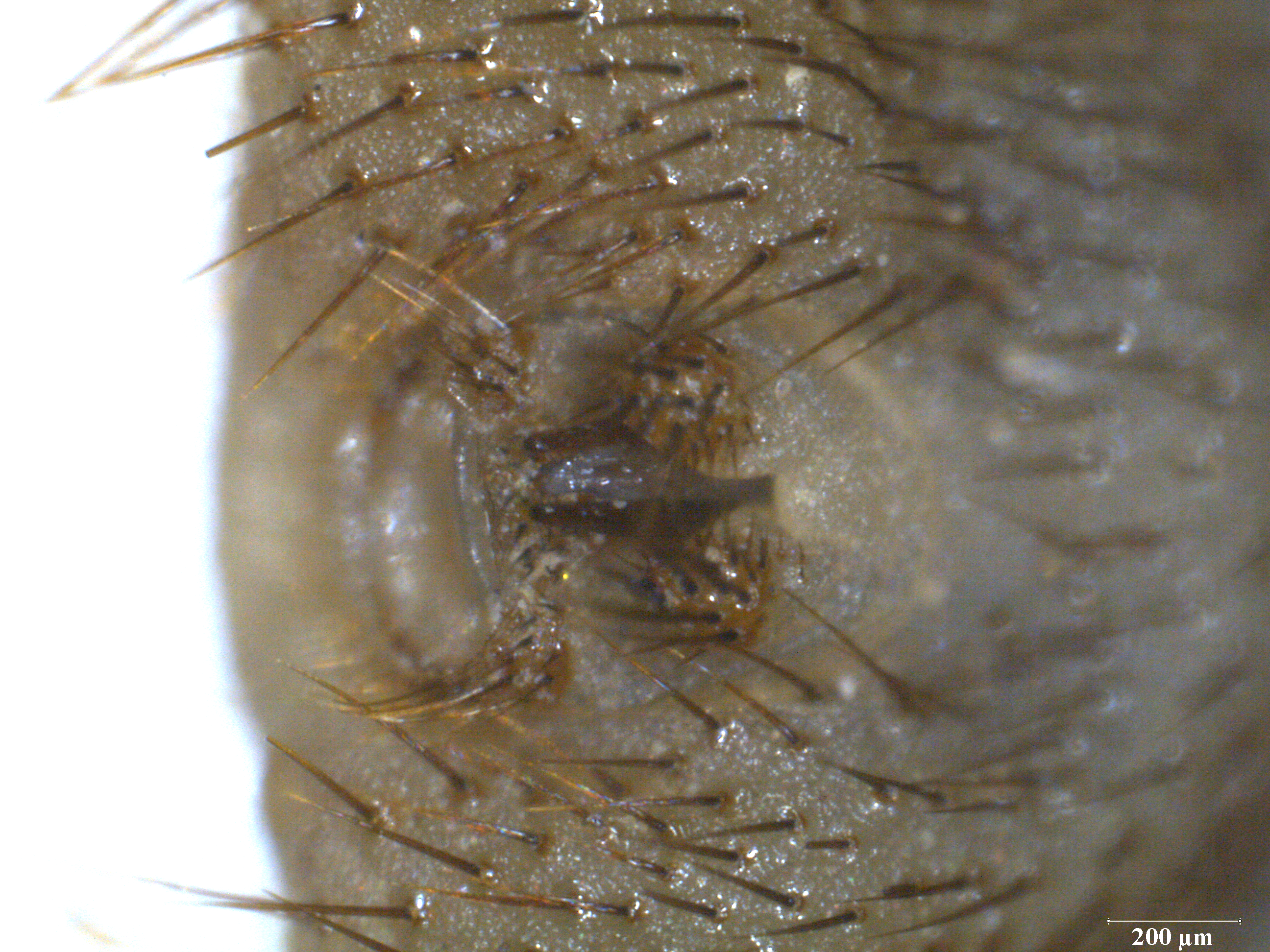

Supplement: Supplementary file 1 [file genes-17-00689-s001.zip › Original Images-Figure S1/Figure S1F.tif]
