# Supplementary material for: Complete Mitochondrial Genome of Melophagus ovinus from Qinghai-Tibet Plateau Provides Evidence for D-Loop Length Polymorphism
Source: Genes (Basel). 2026 Jun 11;17(6):689. doi: 10.3390/genes17060689 (PMC13299176; doi:10.3390/genes17060689)
Supplement: Supplementary file 1 [file Figure S2.pdf › Figure S2.pdf]

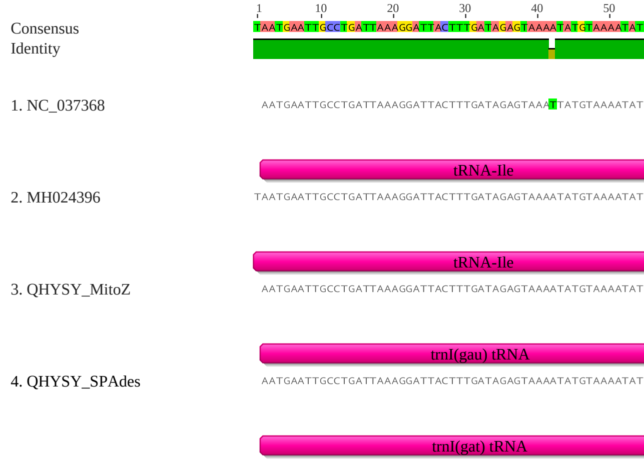

1
10
20
30
40
50
Consensus
TAATGAATTGCCTGATTAAAGGATTACTTTGATAGAGTAAAATATGTAAAATAT
Identity
1. NC_037368
AATGAATTGCCTGATTAAAGGATTACTTTGATAGAGTAAATTATGTAAAATAT
tRNA-Ile
2. MH024396
TAATGAATTGCCTGATTAAAGGATTACTTTGATAGAGTAAAATATGTAAAATAT
tRNA-Ile
3. QHYSY_MitoZ
AATGAATTGCCTGATTAAAGGATTACTTTGATAGAGTAAAATATGTAAAATAT
trnI(gau) tRNA
4. QHYSY_SPAdes
AATGAATTGCCTGATTAAAGGATTACTTTGATAGAGTAAAATATGTAAAATAT
trnI(gat) tRNA

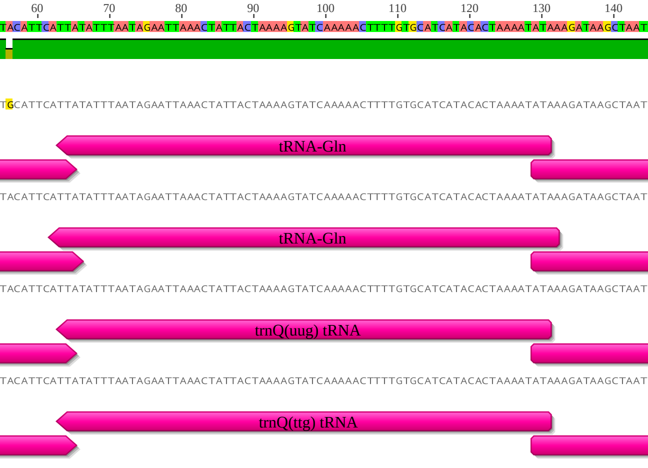
60
70
80
90
100
110
120
130
140
TACATTCATTATATTTAATAGAATTAAACTATTACTAAAAGTATCAAAAACTTTTGTGCATCATACACTAAAATATAAAGATAAGCTAAT
TGCATTCATTATATTTAATAGAATTAAACTATTACTAAAAGTATCAAAAACTTTTGTGCATCATACACTAAAATATAAAGATAAGCTAAT
tRNA-Gln
TACATTCATTATATTTAATAGAATTAAACTATTACTAAAAGTATCAAAAACTTTTGTGCATCATACACTAAAATATAAAGATAAGCTAAT
tRNA-Gln
TACATTCATTATATTTAATAGAATTAAACTATTACTAAAAGTATCAAAAACTTTTGTGCATCATACACTAAAATATAAAGATAAGCTAAT
trnQ(uug) tRNA
TACATTCATTATATTTAATAGAATTAAACTATTACTAAAAGTATCAAAAACTTTTGTGCATCATACACTAAAATATAAAGATAAGCTAAT
trnQ(ttg) tRNA

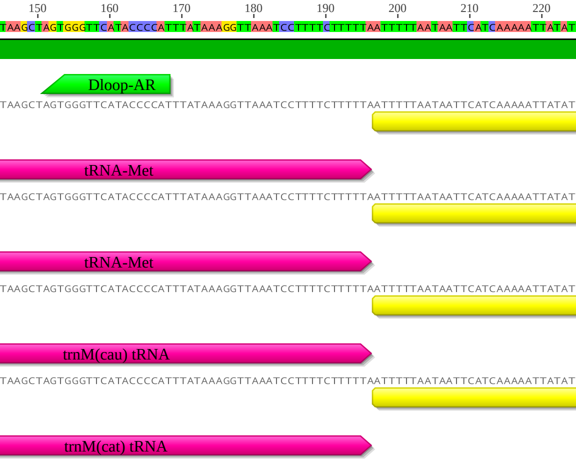
150
160
170
180
190
200
210
220
TAAGCTAGTGGGTTCATACCCCATTTATAAAGGTTAAATCCTTTTCTTTTTAATTTTTAATAATTCATCAAAAATTATAT
Dloop-AR
TAAGCTAGTGGGTTCATACCCCATTTATAAAGGTTAAATCCTTTTCTTTTTAATTTTTAATAATTCATCAAAAATTATAT
tRNA-Met
TAAGCTAGTGGGTTCATACCCCATTTATAAAGGTTAAATCCTTTTCTTTTTAATTTTTAATAATTCATCAAAAATTATAT
tRNA-Met
TAAGCTAGTGGGTTCATACCCCATTTATAAAGGTTAAATCCTTTTCTTTTTAATTTTTAATAATTCATCAAAAATTATAT
trnM(cau) tRNA
TAAGCTAGTGGGTTCATACCCCATTTATAAAGGTTAAATCCTTTTCTTTTTAATTTTTAATAATTCATCAAAAATTATAT
trnM(cat) tRNA

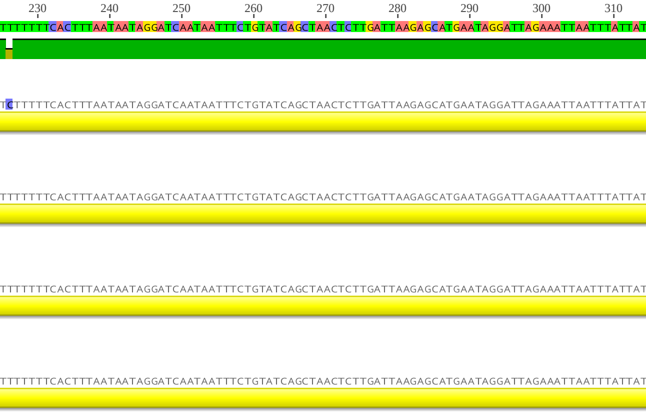
230 240 250 260 270 280 290 300 310
TTTTTTTCACTTTAATAATAGGATCAATAATTTCTGTATCAGCTAACTCTTGATTAAGAGCATGAATAGGATTAGAAATTAATTTATTAT
TCTTTTTCACTTTAATAATAGGATCAATAATTTCTGTATCAGCTAACTCTTGATTAAGAGCATGAATAGGATTAGAAATTAATTTATTAT
TTTTTTTCACTTTAATAATAGGATCAATAATTTCTGTATCAGCTAACTCTTGATTAAGAGCATGAATAGGATTAGAAATTAATTTATTAT
TTTTTTTCACTTTAATAATAGGATCAATAATTTCTGTATCAGCTAACTCTTGATTAAGAGCATGAATAGGATTAGAAATTAATTTATTAT
TTTTTTTCACTTTAATAATAGGATCAATAATTTCTGTATCAGCTAACTCTTGATTAAGAGCATGAATAGGATTAGAAATTAATTTATTAT

320 330 340 350 360 370 380

CTTTTATCCCCCTAATAAGAGATATAAAATTAATATCTACTGAATCTTCATTGAAATATTTCTTAATTCAAGC

CTTTTATCCCCCTAATAAGAGATATAAAATTAATATCTACTGAAGCTTCATTGAAATATTTCTTAATTCAAGC

CTTTTATCCCCCTAATAAGAGATATAAAATTAATATCTACTGAATCTTCATTGAAATATTTCTTAATTCAAGC

CTTTTATCCCCCTAATAAGAGATATAAAATTAATATCTACTGAATCTTCATTGAAATATTTCTTAATTCAAGC

CTTTTATCCCCCTAATAAGAGATATAAAATTAATATCTACTGAATCTTCATTGAAATATTTCTTAATTCAAGC

390 400 410 420 430 440 450 460

TTTAGCTTCTATAATTTTATTGTTTTCTATAATTTCAATGTATATAAAAATGAATATATTAAATTATTATTATTG

TTTAGCTTCTATAATTTTATTGTTTTCTATAATTTCAATGTATATAAAAATGAATATATTAAATTATTATTACTG

TTTAGCTTCTATAATTTTATTGTTTTCTATAATTTCAATGTATATAAAAATGAATATATTAAATTATTATTATTG

TTTAGCTTCTATAATTTTATTGTTTTCTATAATTTCAATGTATATAAAAATGAATATATTAAATTATTATTATTG

TTTAGCTTCTATAATTTTATTGTTTTCTATAATTTCAATGTATATAAAAATGAATATATTAAATTATTATTATTG

470 480 490 500 510 520 530 540

TAAATTAATAATTATTTCATCACTATTAATAAAAATAGGATCAGCTCCTTTTCATTTTTGATTTCCTATAATTATAGAAGGTTTAT

TAAATTAATAATTATTTCATCACTATTAATAAAAATAGGATCAGCTCCTTTTCATTTTTGATTTCCTATAATTATAGAAGGTTTAT

TAAATTAATAATTATTTCATCACTATTAATAAAAATAGGATCAGCTCCTTTTCATTTTTGATTTCCTATAATTATAGAAGGTTTAT

TAAATTAATAATTATTTCATCACTATTAATAAAAATAGGATCAGCTCCTTTTCATTTTTGATTTCCTATAATTATAGAAGGTTTAT

TAAATTAATAATTATTTCATCACTATTAATAAAAATAGGATCAGCTCCTTTTCATTTTTGATTTCCTATAATTATAGAAGGTTTAT

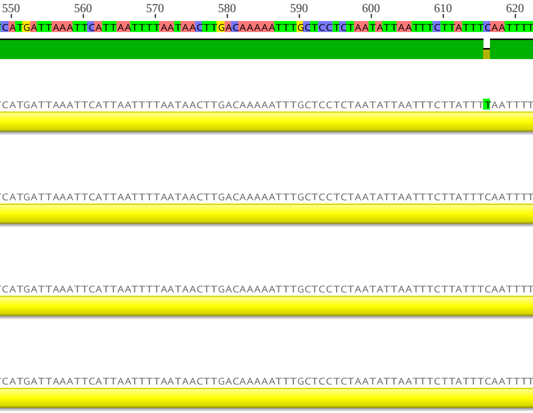

550
560
570
580
590
600
610
620
CATGATTAAATTCATTAATTTTAATAACTTGACAAAAATTTGCTCCTCTAATATTAATTTCTTATTTCAATTTT
CATGATTAAATTCATTAATTTTAATAACTTGACAAAAATTTGCTCCTCTAATATTAATTTCTTATTTTAATTTT
CATGATTAAATTCATTAATTTTAATAACTTGACAAAAATTTGCTCCTCTAATATTAATTTCTTATTTCAATTTT
CATGATTAAATTCATTAATTTTAATAACTTGACAAAAATTTGCTCCTCTAATATTAATTTCTTATTTCAATTTT
CATGATTAAATTCATTAATTTTAATAACTTGACAAAAATTTGCTCCTCTAATATTAATTTCTTATTTCAATTTT

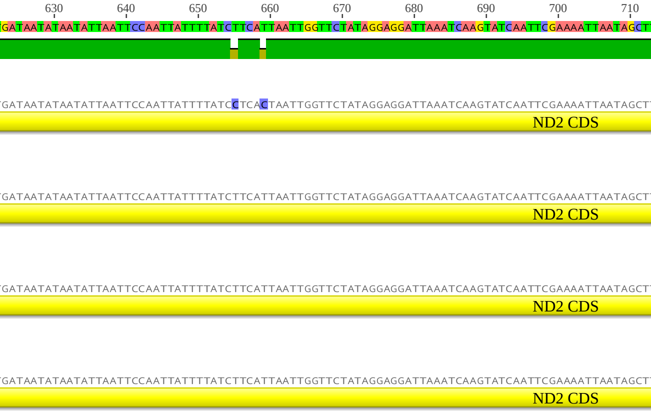
630 640 650 660 670 680 690 700 710
GATAATATAATATTAATTCCAATTATTTTATCTTCATTAATTGGTTCTATAGGAGGATTAAATCAAGTATCAATTCGAAAATTAATAGCT
GATAATATAATATTAATTCCAATTATTTTATCCTCACTAATTGGTTCTATAGGAGGATTAAATCAAGTATCAATTCGAAAATTAATAGCT
ND2 CDS
GATAATATAATATTAATTCCAATTATTTTATCTTCATTAATTGGTTCTATAGGAGGATTAAATCAAGTATCAATTCGAAAATTAATAGCT
ND2 CDS
GATAATATAATATTAATTCCAATTATTTTATCTTCATTAATTGGTTCTATAGGAGGATTAAATCAAGTATCAATTCGAAAATTAATAGCT
ND2 CDS
GATAATATAATATTAATTCCAATTATTTTATCTTCATTAATTGGTTCTATAGGAGGATTAAATCAAGTATCAATTCGAAAATTAATAGCT
ND2 CDS

720 730 740 750 760 770 780 790

TACTCATCAATTAATCATTTAAGATGAATATTAATAAGAATTATAAATAATAATAACATTTGAATTTTATATTTTTTAATTTATT

TACTCATCAATCAATCATTTAAGATGAATATTAATAAGAATTATAAATAATAATAATATTTGAATTTTATATTTTTTAATTTATT

TACTCATCAATTAATCATTTAAGATGAATATTAATAAGAATTATAAATAATAATAACATTTGAATTTTATATTTTTTAATTTATT

TACTCATCAATTAATCATTTAAGATGAATATTAATAAGAATTATAAATAATAATAACATTTGAATTTTATATTTTTTAATTTATT

TACTCATCAATTAATCATTTAAGATGAATATTAATAAGAATTATAAATAATAATAACATTTGAATTTTATATTTTTTAATTTATT

800 810 820 830 840 850 860 870

CATTATTAAATTTTATTATTACAATAATATTTCATGAATTTAAAATTTCTTATATTAACCAATTAACATTTATAATT

CATTATTAAATTTTATTATTACAATAATATTTCATGTATTTAAAATTTCTTATATTAATCAATTAACATTTATAATT

CATTATTAAATTTTATTATTACAATAATATTTCATGAATTTAAAATTTCTTATATTAACCAATTAACATTTATAATT

CATTATTAAATTTTATTATTACAATAATATTTCATGAATTTAAAATTTCTTATATTAACCAATTAACATTTATAATT

CATTATTAAATTTTATTATTACAATAATATTTCATGAATTTAAAATTTCTTATATTAACCAATTAACATTTATAATT

880 890 900 910 920 930 940 950

TTTAATTCAAAATTAATAAAATTTTTTATTTTTATAAATTTATTATCTTTAGGAGGATTACCTCCTTTCTTAGGATTTTTTCC

TTTAATTCAAAATTAATAAAATTTTTTATTTTTATAAATTTATTATCTTTAGGAGGATTACCTCCTTTCTTAGGATTTTTTCC

TTTAATTCAAAATTAATAAAATTTTTTATTTTTATAAATTTATTATCTTTAGGAGGATTACCTCCTTTCTTAGGATTTTTTCC

TTTAATTCAAAATTAATAAAATTTTTTATTTTTATAAATTTATTATCTTTAGGAGGATTACCTCCTTTCTTAGGATTTTTTCC

TTTAATTCAAAATTAATAAAATTTTTTATTTTTATAAATTTATTATCTTTAGGAGGATTACCTCCTTTCTTAGGATTTTTTCC

960 970 980 990 1,000 1,010 1,020 1,030 1,040

TAAATGACTAATTATTCAAGAAATAGTAATAAACAAACAAATTATATTAATATTAATTTTAATAATAACAACATTAATTTCATTATTC

TAAATGACTAATTATTCAAGAAATAGTAATAAATAAACAAATTATATTAATATTAATTTTAATAATAACAACATTAATTTCATTATTC

TAAATGACTAATTATTCAAGAAATAGTAATAAACAAACAAATTATATTAATATTAATTTTAATAATAACAACATTAATTTCATTATTC

TAAATGACTAATTATTCAAGAAATAGTAATAAACAAACAAATTATATTAATATTAATTTTAATAATAACAACATTAATTTCATTATTC

TAAATGACTAATTATTCAAGAAATAGTAATAAACAAACAAATTATATTAATATTAATTTTAATAATAACAACATTAATTTCATTATTC

1,050 1,060 1,070 1,080 1,090 1,100 1,110 1,120

TATTATATTCGAATTAGATACACAACTTTTTTATTAAATCATTATGAAATTAATTGAATTAAATTTTCTTTTCACAGAAATT

TATTATATTCGAATTAGATACACAACTTTTTTATTAAATCATTATGAAATTAATTGAATTAAATTTTCTTTTCACAGAAATT

TATTATATTCGAATTAGATACACAACTTTTTTATTAAATCATTATGAAATTAATTGAATTAAATTTTCTTTTCACAGAAATT

TATTATATTCGAATTAGATACACAACTTTTTTATTAAATCATTATGAAATTAATTGAATTAAATTTTCTTTTCACAGAAATT

TATTATATTCGAATTAGATACACAACTTTTTTATTAAATCATTATGAAATTAATTGAATTAAATTTTCTTTTCACAGAAATT

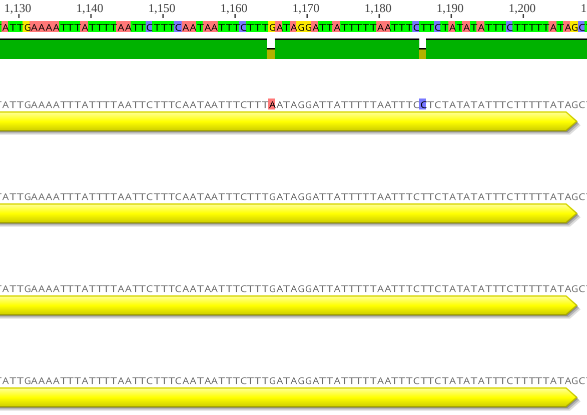

1,130 1,140 1,150 1,160 1,170 1,180 1,190 1,200
ATTGAAAATTTATTTTAATTCTTTCAATAATTTCTTTGATAGGATTATTTTTAATTTCTTCTATATATTTCTTTTTATAGC
TATTGAAAATTTATTTTAATTCTTTCAATAATTTCTTTAATAGGATTATTTTTAATTTCCTCTATATATTTCTTTTTATAGC
TATTGAAAATTTATTTTAATTCTTTCAATAATTTCTTTGATAGGATTATTTTTAATTTCTTCTATATATTTCTTTTTATAGC
TATTGAAAATTTATTTTAATTCTTTCAATAATTTCTTTGATAGGATTATTTTTAATTTCTTCTATATATTTCTTTTTATAGC
TATTGAAAATTTATTTTAATTCTTTCAATAATTTCTTTGATAGGATTATTTTTAATTTCTTCTATATATTTCTTTTTATAGC

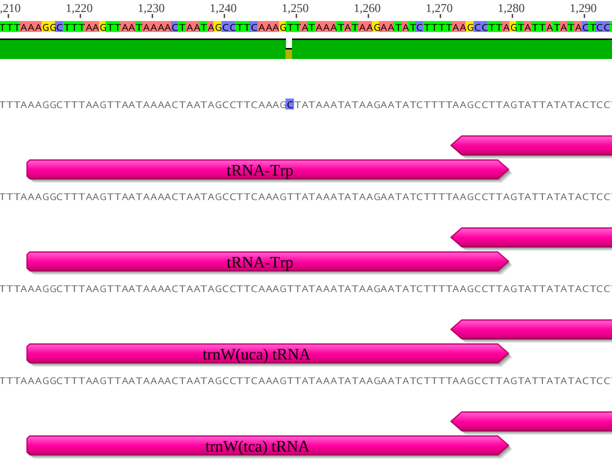

,210 1,220 1,230 1,240 1,250 1,260 1,270 1,280 1,290
TTTAAAGGCTTTAAGTTAATAAAACTAATAGCCTTCAAAGTTATAAATATAAGAATATCTTTTAAGCCTTAGTATTATATACTCC
TTTAAAGGCTTTAAGTTAATAAAACTAATAGCCTTCAAAGCTATAAATATAAGAATATCTTTTAAGCCTTAGTATTATATACTCC
tRNA-Trp
TTTAAAGGCTTTAAGTTAATAAAACTAATAGCCTTCAAAGTTATAAATATAAGAATATCTTTTAAGCCTTAGTATTATATACTCC
tRNA-Trp
TTTAAAGGCTTTAAGTTAATAAAACTAATAGCCTTCAAAGTTATAAATATAAGAATATCTTTTAAGCCTTAGTATTATATACTCC
trnW(uca) tRNA
TTTAAAGGCTTTAAGTTAATAAAACTAATAGCCTTCAAAGTTATAAATATAAGAATATCTTTTAAGCCTTAGTATTATATACTCC
trnW(tca) tRNA

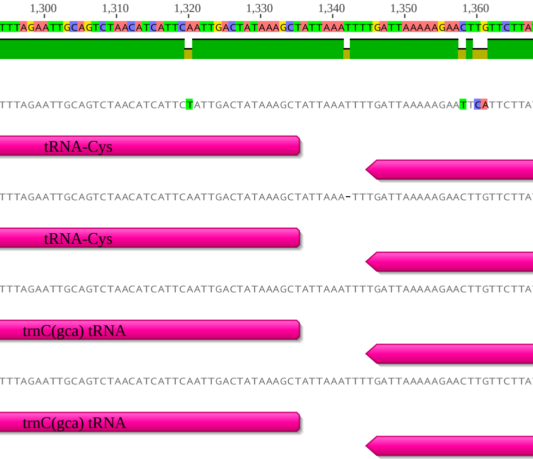

1,300
1,310
1,320
1,330
1,340
1,350
1,360
TTTAGAATTGCAGTCTAACATCATTCAATTGACTATAAAGCTATTAAATTTTGATTAAAAAGAACTTGTTCTTA
TTTAGAATTGCAGTCTAACATCATTCTATTGACTATAAAGCTATTAAATTTTGATTAAAAAGAATTCAATTCTTA
tRNA-Cys
TTTAGAATTGCAGTCTAACATCATTCAATTGACTATAAAGCTATTAAA-TTTGATTAAAAAGAACTTGTTCTTA
tRNA-Cys
TTTAGAATTGCAGTCTAACATCATTCAATTGACTATAAAGCTATTAAATTTTGATTAAAAAGAACTTGTTCTTA
trnC(gca) tRNA
TTTAGAATTGCAGTCTAACATCATTCAATTGACTATAAAGCTATTAAATTTTGATTAAAAAGAACTTGTTCTTA
trnC(gca) tRNA

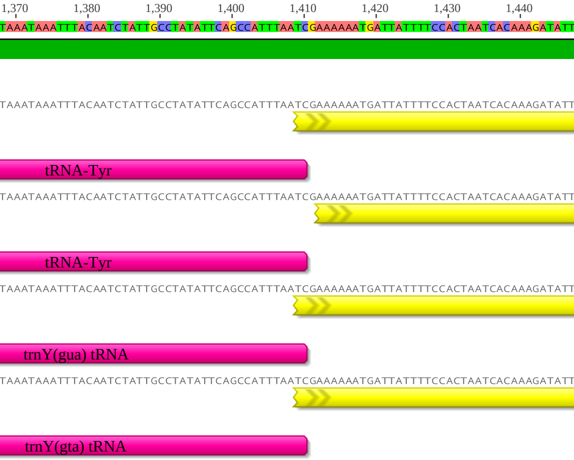

1,370
1,380
1,390
1,400
1,410
1,420
1,430
1,440
TAAATAAATTTACAATCTATTGCCTATATTCAGCCATTTAATCGAAAAAATGATTATTTTCCACTAATCACAAAGATATT
TAAATAAATTTACAATCTATTGCCTATATTCAGCCATTTAATCGAAAAAATGATTATTTTCCACTAATCACAAAGATATT
tRNA-Tyr
TAAATAAATTTACAATCTATTGCCTATATTCAGCCATTTAATCGAAAAAATGATTATTTTCCACTAATCACAAAGATATT
tRNA-Tyr
TAAATAAATTTACAATCTATTGCCTATATTCAGCCATTTAATCGAAAAAATGATTATTTTCCACTAATCACAAAGATATT
trnY(gua) tRNA
TAAATAAATTTACAATCTATTGCCTATATTCAGCCATTTAATCGAAAAAATGATTATTTTCCACTAATCACAAAGATATT
trnY(gta) tRNA

1,450 1,460 1,470 1,480 1,490 1,500 1,510 1,520 1,530

GGCACATTATATTTTATTTTTGGAGCATGATCAGGAATAATTGGTACATCTCTAAGAATTTTAATTCGAATAGAATTAGGACATCCT

GGCACATTATATTTTATTTTTGGAGCATGATCAGGAATAATTGGTACATCTCTAAGAATTTTAATTCGAATAGAATTAGGACATCCT

GGCACATTATATTTTATTTTTGGAGCATGATCAGGAATAATTGGTACATCTCTAAGAATTTTAATTCGAATAGAATTAGGACATCCT

GGCACATTATATTTTATTTTTGGAGCATGATCAGGAATAATTGGTACATCTCTAAGAATTTTAATTCGAATAGAATTAGGACATCCT

GGCACATTATATTTTATTTTTGGAGCATGATCAGGAATAATTGGTACATCTCTAAGAATTTTAATTCGAATAGAATTAGGACATCCT

1,540 1,550 1,560 1,570 1,580 1,590 1,600 1,610 1,620

GGATCTTTAATTGGAAATGATCAAATTTATAATGTAATTGTTACTGCTCATGCATTTATTATAATTTTTTTTATAGTAATACCTACAG

GGATCTTTAATTGGAAATGATCAAATTTATAATGTAATTGTTACTGCTCATGCATTTATTATAATTTTTTTTATAGTAATACCTACAG

GGATCTTTAATTGGAAATGATCAAATTTATAATGTAATTGTTACTGCTCATGCATTTATTATAATTTTTTTTATAGTAATACCTACAG

GGATCTTTAATTGGAAATGATCAAATTTATAATGTAATTGTTACTGCTCATGCATTTATTATAATTTTTTTTATAGTAATACCTACAG

GGATCTTTAATTGGAAATGATCAAATTTATAATGTAATTGTTACTGCTCATGCATTTATTATAATTTTTTTTATAGTAATACCTACAG

1,630 1,640 1,650 1,660 1,670 1,680 1,690 1,700 1,710

TTATTGGTGGCTTTGGAAATTGATTAGTTCCATTAATATTAGGAGCTCCAGATATAGCCTTTCCACGAATAAATAATATAAGATTTTGAT

TTATTGGTGGATTTGGAAATTGATTAGTTCCATTAATATTAGGAGCTCCAGATATAGCCTTTCCACGAATAAATAATATAAGATTTTGAT

TTATTGGTGGCTTTGGAAATTGATTAGTTCCATTAATATTAGGAGCTCCAGATATAGCCTTTCCACGAATAAATAATATAAGATTTTGAT

TTATTGGTGGCTTTGGAAATTGATTAGTTCCATTAATATTAGGAGCTCCAGATATAGCCTTTCCACGAATAAATAATATAAGATTTTGAT

TTATTGGTGGCTTTGGAAATTGATTAGTTCCATTAATATTAGGAGCTCCAGATATAGCCTTTCCACGAATAAATAATATAAGATTTTGAT

1,720 1,730 1,740 1,750 1,760 1,770 1,780 1,790

TACTTCCTCCTGCTTTAACTCTATTGCTCATAAGTATAATAACGGAATATGGAGTTGGAACAGGTTGAACTATTTATCCTCC

TACTTCCTCCTGCTTTAACTCTATTGCTCATAAGTATAATAACGGAATATGGAGTTGGAACAGGTTGAACTATTTATCCTCC

TACTTCCTCCTGCTTTAACTCTATTGCTCATAAGTATAATAACGGAATATGGAGTTGGAACAGGTTGAACTATTTATCCTCC

TACTTCCTCCTGCTTTAACTCTATTGCTCATAAGTATAATAACGGAATATGGAGTTGGAACAGGTTGAACTATTTATCCTCC

TACTTCCTCCTGCTTTAACTCTATTGCTCATAAGTATAATAACGGAATATGGAGTTGGAACAGGTTGAACTATTTATCCTCC

1,800 1,810 1,820 1,830 1,840 1,850 1,860 1,870

TCTCTCATCTATAATTGCTCATAGAGGAGCATCAGTTGATTTAGCTATTTTTTCACTTCATTTAGCAGGAATTTCATC

TCTCTCATCTATAATTGCTCATAGAGGAGCATCAGTTGATTTAGCTATTTTTTCACTTCATTTAGCAGGAATTTCATC

TCTCTCATCTATAATTGCTCATAGAGGAGCATCAGTTGATTTAGCTATTTTTTCACTTCATTTAGCAGGAATTTCATC

TCTCTCATCTATAATTGCTCATAGAGGAGCATCAGTTGATTTAGCTATTTTTTCACTTCATTTAGCAGGAATTTCATC

TCTCTCATCTATAATTGCTCATAGAGGAGCATCAGTTGATTTAGCTATTTTTTCACTTCATTTAGCAGGAATTTCATC

1,880 1,890 1,900 1,910 1,920 1,930 1,940 1,950

TATTCTTGGAGCAGTAAATTTTATCACAACAATTATTAATATACGTTCATATGGAATTTCATTTGATCGAATACCTTTATTTG

TATTCTTGGAGCAGTAAATTTTATCACAACAATTATTAATATACGTTCATATGGAATTTCATTTGATCGAATACCTTTATTTG

TATTCTTGGAGCAGTAAATTTTATCACAACAATTATTAATATACGTTCATATGGAATTTCATTTGATCGAATACCTTTATTTG

TATTCTTGGAGCAGTAAATTTTATCACAACAATTATTAATATACGTTCATATGGAATTTCATTTGATCGAATACCTTTATTTG

TATTCTTGGAGCAGTAAATTTTATCACAACAATTATTAATATACGTTCATATGGAATTTCATTTGATCGAATACCTTTATTTG

1,960 1,970 1,980 1,990 2,000 2,010 2,020 2,030 2,040

TTTGATCAGTAATAATTACAGCAATTTTATTATTATTATCATTACCTGTATTAGCAGGAGCAATTACTATATTATTAACAGATCGAAATTT

TTTGATCAGTAATAATTACAGCAATTTTATTATTATTATCATTACCTGTATTAGCAGGAGCAATTACTATATTATTAACAGATCGAAATTT

TTTGATCAGTAATAATTACAGCAATTTTATTATTATTATCATTACCTGTATTAGCAGGAGCAATTACTATATTATTAACAGATCGAAATTT

TTTGATCAGTAATAATTACAGCAATTTTATTATTATTATCATTACCTGTATTAGCAGGAGCAATTACTATATTATTAACAGATCGAAATTT

TTTGATCAGTAATAATTACAGCAATTTTATTATTATTATCATTACCTGTATTAGCAGGAGCAATTACTATATTATTAACAGATCGAAATTT

2,050 2,060 2,070 2,080 2,090 2,100 2,110 2,120

TAAATACATCATTTTTTGATCCAGCTGGAGGAGGTGATCCTATTCTTTATCAACATTTATTTTGATTCTTTGGTCAT

TAAATACATCATTTTTTGATCCAGCTGGAGGAGGTGATCCTATTCTTTATCAACATTTATTTTGATTCTTTGGTCAT

TAAATACATCATTTTTTGATCCAGCTGGAGGAGGTGATCCTATTCTTTATCAACATTTATTTTGATTCTTTGGTCAT

TAAATACATCATTTTTTGATCCAGCTGGAGGAGGTGATCCTATTCTTTATCAACATTTATTTTGATTCTTTGGTCAT

TAAATACATCATTTTTTGATCCAGCTGGAGGAGGTGATCCTATTCTTTATCAACATTTATTTTGATTCTTTGGTCAT

2,130 2,140 2,150 2,160 2,170 2,180 2,190 2,200

CCAGAAGTTTATATTTTAATTCTTCCAGGATTTGGAATAATCTCTCATATTATTAGTCAAGAATCTGGAAAAAAAGAAAC

TCCAGAAGTTTATATTTTAATTCTTCCAGGATTTGGAATAATCTCTCATATTATTAGTCAAGAATCTGGAAAAAAAGAAACT

COX1 CDS

TCCAGAAGTTTATATTTTAATTCTTCCAGGATTTGGAATAATCTCTCATATTATTAGTCAAGAATCTGGAAAAAAAGAAACT

COX1 CDS

TCCAGAAGTTTATATTTTAATTCTTCCAGGATTTGGAATAATCTCTCATATTATTAGTCAAGAATCTGGAAAAAAAGAAACT

COX1 CDS

TCCAGAAGTTTATATTTTAATTCTTCCAGGATTTGGAATAATCTCTCATATTATTAGTCAAGAATCTGGAAAAAAAGAAACT

COX1 CDS

2,210 2,220 2,230 2,240 2,250 2,260 2,270 2,280

TTTTGGTTCATTAGGAATAATTTATGCAATACTAGCTATTGGATTATTAGGATTTATTGTATGAGCTCATCATATATTTACAG

TTTTGGTTCATTAGGAATAATTTATGCAATACTAGCTATTGGATTATTAGGATTTATTGTATGAGCTCATCATATATTTACAG

TTTTGGTTCATTAGGAATAATTTATGCAATACTAGCTATTGGATTATTAGGATTTATTGTATGAGCTCATCATATATTTACAG

TTTTGGTTCATTAGGAATAATTTATGCAATACTAGCTATTGGATTATTAGGATTTATTGTATGAGCTCATCATATATTTACAG

TTTTGGTTCATTAGGAATAATTTATGCAATACTAGCTATTGGATTATTAGGATTTATTGTATGAGCTCATCATATATTTACAG

2,290 2,300 2,310 2,320 2,330 2,340 2,350 2,360

TTGGAATAGATGTAGATACACGAGCTTACTTTACTTCTGCTACAATAATTATCGCTGTTCCAACAGGAATTAAAATTTT

TTGGAATAGATGTAGATACACGAGCTTACTTTACTTCTGCTACAATAATTATCGCTGTTCCAACAGGAATTAAAATTTT

TTGGAATAGATGTAGATACACGAGCTTACTTTACTTCTGCTACAATAATTATCGCTGTTCCAACAGGAATTAAAATTTT

TTGGAATAGATGTAGATACACGAGCTTACTTTACTTCTGCTACAATAATTATCGCTGTTCCAACAGGAATTAAAATTTT

TTGGAATAGATGTAGATACACGAGCTTACTTTACTTCTGCTACAATAATTATCGCTGTTCCAACAGGAATTAAAATTTT

2,370 2,380 2,390 2,400 2,410 2,420 2,430 2,440 2,450

CAGTTGATTAGCAACTCTTCATGGTACACAATTAAACTATTCCCCTGCTATTATATGAGCTTTAGGATTTATTTTTTTATTTACAGTT

TCAGTTGATTAGCAACTCTTCATGGTACACAATTAAACTATTCCCCTGCTATTATATGAGCTTTAGGATTTATTTTTTTATTTACAGTT

TCAGTTGATTAGCAACTCTTCATGGTACACAATTAAACTATTCCCCTGCTATTATATGAGCTTTAGGATTTATTTTTTTATTTACAGTT

TCAGTTGATTAGCAACTCTTCATGGTACACAATTAAACTATTCCCCTGCTATTATATGAGCTTTAGGATTTATTTTTTTATTTACAGTT

TCAGTTGATTAGCAACTCTTCATGGTACACAATTAAACTATTCCCCTGCTATTATATGAGCTTTAGGATTTATTTTTTTATTTACAGTT

2,460 2,470 2,480 2,490 2,500 2,510 2,520 2,530 2,540

GGAGGATTAACAGGAGTAGTATTAGCAAATTCATCAATTGATATTATTTTACATGATACTTATTATGTTGTTGCTCATTTCCATTATGTA

GGAGGATTAACAGGAGTAGTATTAGCAAATTCATCAATTGATATTATTTTACATGATACTTATTATGTTGTTGCTCATTTCCATTATGTA

GGAGGATTAACAGGAGTAGTATTAGCAAATTCATCAATTGATATTATTTTACATGATACTTATTATGTTGTTGCTCATTTCCATTATGTA

GGAGGATTAACAGGAGTAGTATTAGCAAATTCATCAATTGATATTATTTTACATGATACTTATTATGTTGTTGCTCATTTCCATTATGTA

GGAGGATTAACAGGAGTAGTATTAGCAAATTCATCAATTGATATTATTTTACATGATACTTATTATGTTGTTGCTCATTTCCATTATGTA

2,550 2,560 2,570 2,580 2,590 2,600 2,610 2,620

TTATCTATAGGAGCTGTATTTGCAATTATAGGAGGATTTATTCATTGATATCCCTTATTTACTGGATTAACATTAAATGATAA

TTATCTATAGGAGCTGTATTTGCAATTATAGGAGGATTTATTCATTGATATCCCTTATTTACTGGATTAACATTAAATGATAAT

TTATCTATAGGAGCTGTATTTGCAATTATAGGAGGATTTATTCATTGATATCCCTTATTTACTGGATTAACATTAAATGATAAT

TTATCTATAGGAGCTGTATTTGCAATTATAGGAGGATTTATTCATTGATATCCCTTATTTACTGGATTAACATTAAATGATAAT

TTATCTATAGGAGCTGTATTTGCAATTATAGGAGGATTTATTCATTGATATCCCTTATTTACTGGATTAACATTAAATGATAAT

2,630 2,640 2,650 2,660 2,670 2,680 2,690 2,700 2,710

TTATCTAAAAACTCAATTTTTAATTATATTTATTGGAGTAAATTTAACATTTTTTCCTCAACATTTTCTTGGTTTATCTGGATTACCTCG

TTATCTAAAAACTCAATTTTTAATTATATTTATTGGAGTAAATTTAACATTTTTTCCTCAACATTTTCTTGGTTTATCTGGATTACCTCG

TTATCTAAAAACTCAATTTTTAATTATATTTATTGGAGTAAATTTAACATTTTTTCCTCAACATTTTCTTGGTTTATCTGGATTACCTCG

TTATCTAAAAACTCAATTTTTAATTATATTTATTGGAGTAAATTTAACATTTTTTCCTCAACATTTTCTTGGTTTATCTGGATTACCTCG

TTATCTAAAAACTCAATTTTTAATTATATTTATTGGAGTAAATTTAACATTTTTTCCTCAACATTTTCTTGGTTTATCTGGATTACCTCG

2,720 2,730 2,740 2,750 2,760 2,770 2,780 2,790 2,800

TCGATATTCTGATTATCCAGATGCTTATATAATATGAAATTTAATTTCAACAATAGGATCAACAATTTCATTTCTTAGAATTATATATTTT

TCGATATTCTGATTATCCAGATGCTTATATAATATGAAATTTAATTTCAACAATAGGATCAACAATTTCATTTCTTAGAATTATATATTTT

TCGATATTCTGATTATCCAGATGCTTATATAATATGAAATTTAATTTCAACAATAGGATCAACAATTTCATTTCTTAGAATTATATATTTT

TCGATATTCTGATTATCCAGATGCTTATATAATATGAAATTTAATTTCAACAATAGGATCAACAATTTCATTTCTTAGAATTATATATTTT

TCGATATTCTGATTATCCAGATGCTTATATAATATGAAATTTAATTTCAACAATAGGATCAACAATTTCATTTCTTAGAATTATATATTTT

2,810 2,820 2,830 2,840 2,850 2,860 2,870 2,880

TCTTTTTATCATCTGAGAAAGAATTGTTTGTAAACGAATAACTTTATTCCCTATTCAATTAAATTCATCTATTGAAT

TCTTTTTATCATCTGAGAAAGAATTGTTTGTAAACGAATAACTTTATTCCCTATTCAATTAAATTCATCTATTGAAT

TCTTTTTATCATCTGAGAAAGAATTGTTTGTAAACGAATAACTTTATTCCCTATTCAATTAAATTCATCTATTGAAT

TCTTTTTATCATCTGAGAAAGAATTGTTTGTAAACGAATAACTTTATTCCCTATTCAATTAAATTCATCTATTGAAT

TCTTTTTATCATCTGAGAAAGAATTGTTTGTAAACGAATAACTTTATTCCCTATTCAATTAAATTCATCTATTGAAT

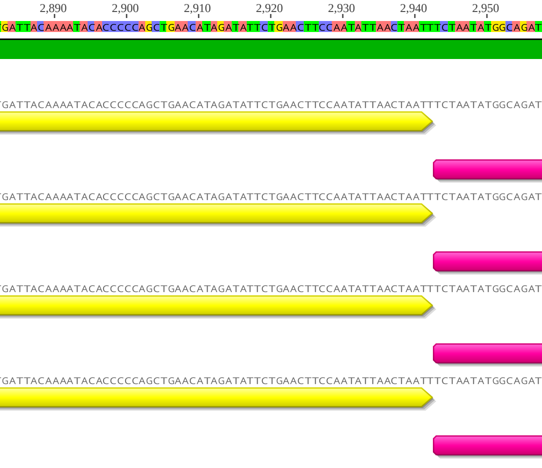
2,890
2,900
2,910
2,920
2,930
2,940
2,950
GATTACAAAATACACCCCCAGCTGAACATAGATATTCTGAACTTCCAATATTAACTAATTTCTAATATGGCAGAT
GATTACAAAATACACCCCCAGCTGAACATAGATATTCTGAACTTCCAATATTAACTAATTTCTAATATGGCAGAT
GATTACAAAATACACCCCCAGCTGAACATAGATATTCTGAACTTCCAATATTAACTAATTTCTAATATGGCAGAT
GATTACAAAATACACCCCCAGCTGAACATAGATATTCTGAACTTCCAATATTAACTAATTTCTAATATGGCAGAT
GATTACAAAATACACCCCCAGCTGAACATAGATATTCTGAACTTCCAATATTAACTAATTTCTAATATGGCAGAT

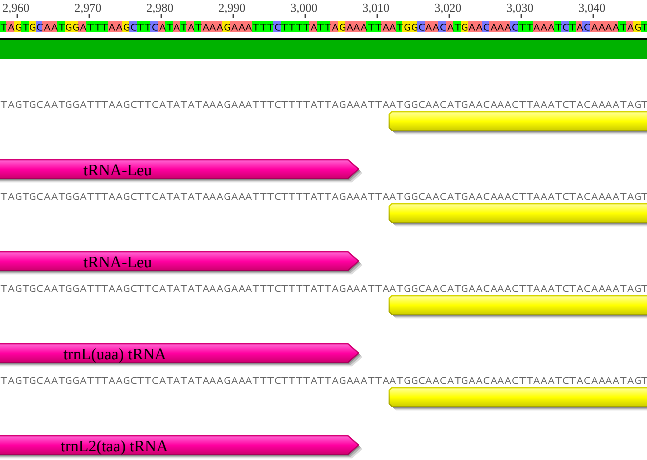
2,960
2,970
2,980
2,990
3,000
3,010
3,020
3,030
3,040
TAGTGCAATGGATTTAAGCTTCATATATAAAGAAATTTCTTTTATTAGAAATTAATGGCAACATGAACAAACTTAAATCTACAAAATAGT
TAGTGCAATGGATTTAAGCTTCATATATAAAGAAATTTCTTTTATTAGAAATTAATGGCAACATGAACAAACTTAAATCTACAAAATAGT
tRNA-Leu
TAGTGCAATGGATTTAAGCTTCATATATAAAGAAATTTCTTTTATTAGAAATTAATGGCAACATGAACAAACTTAAATCTACAAAATAGT
tRNA-Leu
TAGTGCAATGGATTTAAGCTTCATATATAAAGAAATTTCTTTTATTAGAAATTAATGGCAACATGAACAAACTTAAATCTACAAAATAGT
trnL(uaa) tRNA
TAGTGCAATGGATTTAAGCTTCATATATAAAGAAATTTCTTTTATTAGAAATTAATGGCAACATGAACAAACTTAAATCTACAAAATAGT
trnL2(taa) tRNA

```
3,050     3,060     3,070     3,080     3,090     3,100     3,110     3,120     3,130
GCCTCTCCTCTTATAGAACAATTAATTTTTTTTCATGATCATACTTTAATAATTTTAATTATAATTACAACTATAGTAAGATATC

TGCCTCTCCTCTTATAGAACAATTAATTTTTTTTCATGATCATACTTTAATAATTTTAATTATAATTACAACTATAGTAAGATATC

TGCCTCTCCTCTTATAGAACAATTAATTTTTTTTCATGATCATACTTTAATAATTTTAATTATAATTACAACTATAGTAAGATATC

TGCCTCTCCTCTTATAGAACAATTAATTTTTTTTCATGATCATACTTTAATAATTTTAATTATAATTACAACTATAGTAAGATATC

TGCCTCTCCTCTTATAGAACAATTAATTTTTTTTCATGATCATACTTTAATAATTTTAATTATAATTACAACTATAGTAAGATATC
```

3,140 3,150 3,160 3,170 3,180 3,190 3,200 3,210

TAATATTCATATTATTTTTTAATAAACTAATTAATCGATTTTTATTACATGGTCAAATTATTGAAATTATTTGAACAATTATT

TAATATTCATATTATTTTTTAATAAACTAATTAATCGATTTTTATTACATGGTCAAATTATTGAAATTATTTGAACAATTATT

TAATATTCATATTATTTTTTAATAAACTAATTAATCGATTTTTATTACATGGTCAAATTATTGAAATTATTTGAACAATTATT

TAATATTCATATTATTTTTTAATAAACTAATTAATCGATTTTTATTACATGGTCAAATTATTGAAATTATTTGAACAATTATT

TAATATTCATATTATTTTTTAATAAACTAATTAATCGATTTTTATTACATGGTCAAATTATTGAAATTATTTGAACAATTATT

3,220 3,230 3,240 3,250 3,260 3,270 3,280 3,290

CCAGCTATTATTTTATTATTTATTGCATTTCCCTCATTACGATTATTATATTTATTAGATGAAATTAATGAACCAATAATTT

TCCAGCTATTATTTTATTATTTATTGCATTTCCCTCATTACGATTATTATATTTATTAGATGAAATTAATGAACCAATAATTT

TCCAGCTATTATTTTATTATTTATTGCATTTCCCTCATTACGATTATTATATTTATTAGATGAAATTAATGAACCAATAATTT

TCCAGCTATTATTTTATTATTTATTGCATTTCCCTCATTACGATTATTATATTTATTAGATGAAATTAATGAACCAATAATTT

TCCAGCTATTATTTTATTATTTATTGCATTTCCCTCATTACGATTATTATATTTATTAGATGAAATTAATGAACCAATAATTT

3,300 3,310 3,320 3,330 3,340 3,350 3,360 3,370 3,380

CCATTAAAGCAATTGGTCATCAATGATATTGAAAATATGAATATTCTGATTTTAAAAATATTGAATTTGATTCATATATAATTCCTAT

CCATTAAAGCAATTGGTCATCAATGATATTGAAAATATGAATATTCTGATTTTAAAAATATTGAATTTGATTCATATATAATTCCTAT

COX2 CDS

CCATTAAAGCAATTGGTCATCAATGATATTGAAAATATGAATATTCTGATTTTAAAAATATTGAATTTGATTCATATATAATTCCTAT

COX2 CDS

CCATTAAAGCAATTGGTCATCAATGATATTGAAAATATGAATATTCTGATTTTAAAAATATTGAATTTGATTCATATATAATTCCTAT

COX2 CDS

CCATTAAAGCAATTGGTCATCAATGATATTGAAAATATGAATATTCTGATTTTAAAAATATTGAATTTGATTCATATATAATTCCTAT

COX2 CDS

3,390 3,400 3,410 3,420 3,430 3,440 3,450 3,460

TAATGAATTACCTACTAATGGTTTCCGATTATTAGATGTAGACAATCGAATAATTATCCCAATAAATGTTCAAATTCGAATT

TAATGAATTACCTACTAATGGTTTCCGATTATTAGATGTAGACAATCGAATAATTATCCCAATAAATGTTCAAATTCGAATT

TAATGAATTACCTACTAATGGTTTCCGATTATTAGATGTAGACAATCGAATAATTATCCCAATAAATGTTCAAATTCGAATT

TAATGAATTACCTACTAATGGTTTCCGATTATTAGATGTAGACAATCGAATAATTATCCCAATAAATGTTCAAATTCGAATT

TAATGAATTACCTACTAATGGTTTCCGATTATTAGATGTAGACAATCGAATAATTATCCCAATAAATGTTCAAATTCGAATT

3,470	3,480	3,490	3,500	3,510	3,520	3,530	3,540

TTAGTAACCGCAACTGATGTAATTCATTCTTGAACTATTCCTTCTTTAGGAATTAAAATAGATGCTACACCTGGT

TTAGTAACCGCAACTGATGTAATTCATTCTTGAACTATTCCTTCTTTAGGAATTAAAATAGATGCTACACCTGGT

TTAGTAACCGCAACTGATGTAATTCATTCTTGAACTATTCCTTCTTTAGGAATTAAAATAGATGCTACACCTGGT

TTAGTAACCGCAACTGATGTAATTCATTCTTGAACTATTCCTTCTTTAGGAATTAAAATAGATGCTACACCTGGT

TTAGTAACCGCAACTGATGTAATTCATTCTTGAACTATTCCTTCTTTAGGAATTAAAATAGATGCTACACCTGGT

3,550 3,560 3,570 3,580 3,590 3,600 3,610 3,620

CGATTAAACCAAACTAATTTTATAATTAATCGACCAGGATTATTTTTTGGACAATGCTCAGAAATTTGTGGAACCAATCATT

TCGATTAAACCAAACTAATTTTATAATTAATCGACCAGGATTATTTTTTGGACAATGCTCAGAAATTTGTGGAACCAATCATT

TCGATTAAACCAAACTAATTTTATAATTAATCGACCAGGATTATTTTTTGGACAATGCTCAGAAATTTGTGGAACCAATCATT

TCGATTAAACCAAACTAATTTTATAATTAATCGACCAGGATTATTTTTTGGACAATGCTCAGAAATTTGTGGAACCAATCATT

TCGATTAAACCAAACTAATTTTATAATTAATCGACCAGGATTATTTTTTGGACAATGCTCAGAAATTTGTGGAACCAATCATT

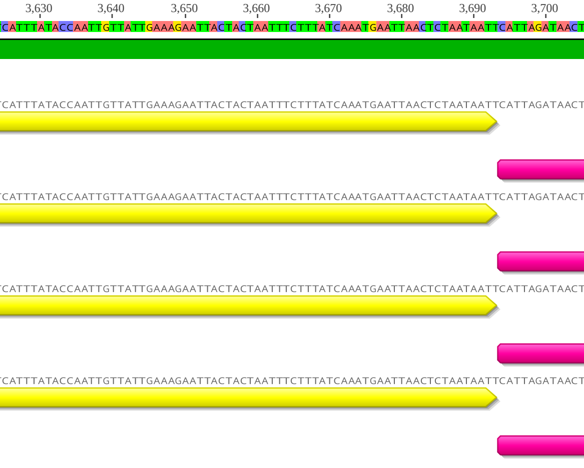
3,630
3,640
3,650
3,660
3,670
3,680
3,690
3,700
CATTTATACCAATTGTTATTGAAAGAATTACTACTAATTTCTTTATCAAATGAATTAACTCTAATAATTCATTAGATAACT
CATTTATACCAATTGTTATTGAAAGAATTACTACTAATTTCTTTATCAAATGAATTAACTCTAATAATTCATTAGATAACT
CATTTATACCAATTGTTATTGAAAGAATTACTACTAATTTCTTTATCAAATGAATTAACTCTAATAATTCATTAGATAACT
CATTTATACCAATTGTTATTGAAAGAATTACTACTAATTTCTTTATCAAATGAATTAACTCTAATAATTCATTAGATAACT
CATTTATACCAATTGTTATTGAAAGAATTACTACTAATTTCTTTATCAAATGAATTAACTCTAATAATTCATTAGATAACT

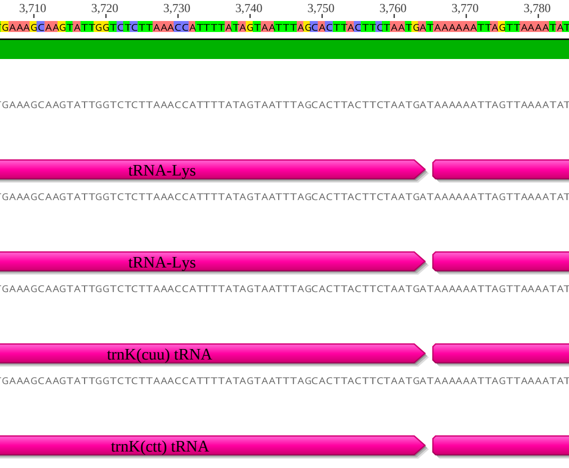

3,710
3,720
3,730
3,740
3,750
3,760
3,770
3,780
GAAAGCAAGTATTGGTCTCTTAAACCATTTTATAGTAATTTAGCACTTACTTCTAATGATAAAAAATTAGTTAAAATAT
tRNA-Lys
GAAAGCAAGTATTGGTCTCTTAAACCATTTTATAGTAATTTAGCACTTACTTCTAATGATAAAAAATTAGTTAAAATAT
tRNA-Lys
GAAAGCAAGTATTGGTCTCTTAAACCATTTTATAGTAATTTAGCACTTACTTCTAATGATAAAAAATTAGTTAAAATAT
trnK(cuu) tRNA
GAAAGCAAGTATTGGTCTCTTAAACCATTTTATAGTAATTTAGCACTTACTTCTAATGATAAAAAATTAGTTAAAATAT
trnK(ctt) tRNA

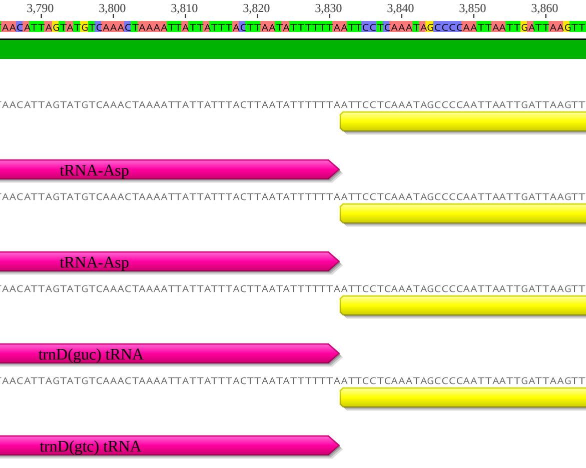

3,790
3,800
3,810
3,820
3,830
3,840
3,850
3,860
AACATTAGTATGTCAAACTAAAATTATTATTTACTTAATATTTTTTAATTCCTCAAATAGCCCCAATTAATTGATTAAGTT
AACATTAGTATGTCAAACTAAAATTATTATTTACTTAATATTTTTTAATTCCTCAAATAGCCCCAATTAATTGATTAAGTT
tRNA-Asp
AACATTAGTATGTCAAACTAAAATTATTATTTACTTAATATTTTTTAATTCCTCAAATAGCCCCAATTAATTGATTAAGTT
tRNA-Asp
AACATTAGTATGTCAAACTAAAATTATTATTTACTTAATATTTTTTAATTCCTCAAATAGCCCCAATTAATTGATTAAGTT
trnD(guc) tRNA
AACATTAGTATGTCAAACTAAAATTATTATTTACTTAATATTTTTTAATTCCTCAAATAGCCCCAATTAATTGATTAAGTT
trnD(gtc) tRNA

3,870 3,880 3,890 3,900 3,910 3,920 3,930 3,940 3,950

TATTTTTTATATTTAATTTTATTTTTATTATTTTATGTATATTAAATTATTTTAATTATAATTTAATAATTACAAAATCAAAAAATAAAA

TATTTTTTATATTTAATTTTATTTTTATTATTTTATGTATATTAAATTATTTTAATTATAATTTAATAATTACAAAATCAAAAAATAAAA

ATP8 CDS

TATTTTTTATATTTAATTTTATTTTTATTATTTTATGTATATTAAATTATTTTAATTATAATTTAATAATTACAAAATCAAAAAATAAAA

ATP8 CDS

TATTTTTTATATTTAATTTTATTTTTATTATTTTATGTATATTAAATTATTTTAATTATAATTTAATAATTACAAAATCAAAAAATAAAA

ATP8 CDS

TATTTTTTATATTTAATTTTATTTTTATTATTTTATGTATATTAAATTATTTTAATTATAATTTAATAATTACAAAATCAAAAAATAAAA

ATP8 CDS

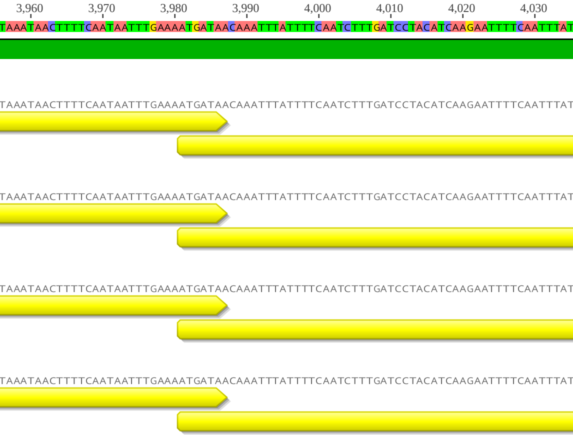
3,960
3,970
3,980
3,990
4,000
4,010
4,020
4,030
TAAATAACTTTTCAATAATTTGAAAATGATAACAAATTTATTTTCAATCTTTGATCCTACATCAAGAATTTTCAATTTAT
TAAATAACTTTTCAATAATTTGAAAATGATAACAAATTTATTTTCAATCTTTGATCCTACATCAAGAATTTTCAATTTAT
TAAATAACTTTTCAATAATTTGAAAATGATAACAAATTTATTTTCAATCTTTGATCCTACATCAAGAATTTTCAATTTAT
TAAATAACTTTTCAATAATTTGAAAATGATAACAAATTTATTTTCAATCTTTGATCCTACATCAAGAATTTTCAATTTAT
TAAATAACTTTTCAATAATTTGAAAATGATAACAAATTTATTTTCAATCTTTGATCCTACATCAAGAATTTTCAATTTAT

4,040 4,050 4,060 4,070 4,080 4,090 4,100 4,110

CTATTAATTGAATTAGTTCTATAATAAGAATTTTAATAATTCCATCAATATATTGAATTATCCCTTCTCGAATTAATTAT

TCTATTAATTGAATTAGTTCTATAATAAGAATTTTAATAATTCCATCAATATATTGAATTATCCCTTCTCGAATTAATTAT

TCTATTAATTGAATTAGTTCTATAATAAGAATTTTAATAATTCCATCAATATATTGAATTATCCCTTCTCGAATTAATTAT

TCTATTAATTGAATTAGTTCTATAATAAGAATTTTAATAATTCCATCAATATATTGAATTATCCCTTCTCGAATTAATTAT

TCTATTAATTGAATTAGTTCTATAATAAGAATTTTAATAATTCCATCAATATATTGAATTATCCCTTCTCGAATTAATTAT

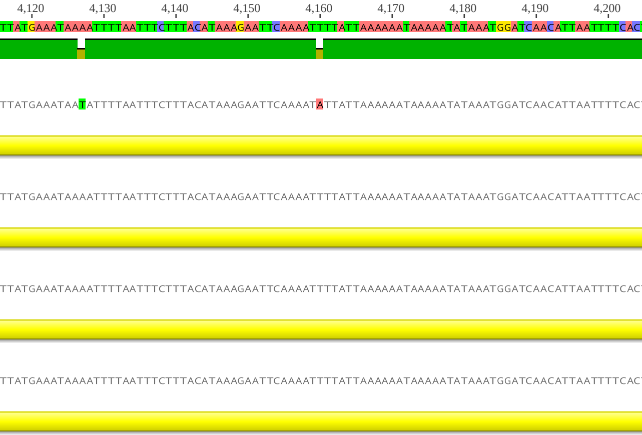
4,120 4,130 4,140 4,150 4,160 4,170 4,180 4,190 4,200
TTATGAAATAAAATTTTAATTTCTTTACATAAAGAATTCAAAATTTTATTAAAAAATAAAAATATAAATGGATCAACATTAATTTTCAC
TTATGAAATAATATTTTAATTTCTTTACATAAAGAATTCAAAATATTATTAAAAAATAAAAATATAAATGGATCAACATTAATTTTCAC
TTATGAAATAAAATTTTAATTTCTTTACATAAAGAATTCAAAATTTTATTAAAAAATAAAAATATAAATGGATCAACATTAATTTTCAC
TTATGAAATAAAATTTTAATTTCTTTACATAAAGAATTCAAAATTTTATTAAAAAATAAAAATATAAATGGATCAACATTAATTTTCAC
TTATGAAATAAAATTTTAATTTCTTTACATAAAGAATTCAAAATTTTATTAAAAAATAAAAATATAAATGGATCAACATTAATTTTCAC

4,210 4,220 4,230 4,240 4,250 4,260 4,270 4,280 4,290

TTCTTTATTTTCAATAATCTTTTTTAATAATTTTATAGGATTATTTCCATATATTTTTACTAGAACCAGACATTTATCTTTAACATTATC

TTCTTTATTTTCAATAATCTTTTTTAATAATTTTATAGGATTATTTCCATATATTTTTACTAGAACCAGACATTTATCTTTAACATTATC

TTCTTTATTTTCAATAATCTTTTTTAATAATTTTATAGGATTATTTCCATATATTTTTACTAGAACCAGACATTTATCTTTAACATTATC

TTCTTTATTTTCAATAATCTTTTTTAATAATTTTATAGGATTATTTCCATATATTTTTACTAGAACCAGACATTTATCTTTAACATTATC

TTCTTTATTTTCAATAATCTTTTTTAATAATTTTATAGGATTATTTCCATATATTTTTACTAGAACCAGACATTTATCTTTAACATTATC

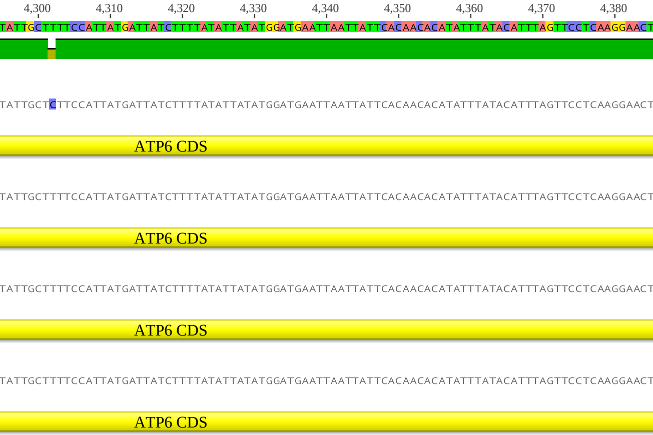
4,300 4,310 4,320 4,330 4,340 4,350 4,360 4,370 4,380
TATTGCTTTTCCATTATGATTATCTTTTATATTATATGGATGAATTAATTATTCACAACACATATTTATACATTTAGTTCCTCAAGGAACT
TATTGCTCTTCCATTATGATTATCTTTTATATTATATGGATGAATTAATTATTCACAACACATATTTATACATTTAGTTCCTCAAGGAACT
ATP6 CDS
TATTGCTTTTCCATTATGATTATCTTTTATATTATATGGATGAATTAATTATTCACAACACATATTTATACATTTAGTTCCTCAAGGAACT
ATP6 CDS
TATTGCTTTTCCATTATGATTATCTTTTATATTATATGGATGAATTAATTATTCACAACACATATTTATACATTTAGTTCCTCAAGGAACT
ATP6 CDS
TATTGCTTTTCCATTATGATTATCTTTTATATTATATGGATGAATTAATTATTCACAACACATATTTATACATTTAGTTCCTCAAGGAACT
ATP6 CDS

4,390 4,400 4,410 4,420 4,430 4,440 4,450 4,460 4,470

TCCATCAATTTTAATACCATTTATAGTATGCATTGAATCAATTAGAAATATAATTCGACCAATTACATTAGCTATTCGATTAACAGCTAATT

TCCATCAATTTTAATACCATTTATAGTATGCATTGAATCAATTAGAAATATAATTCGACCAATTACATTAGCTATTCGATTAACAGCTAAT

TCCATCAATTTTAATACCATTTATAGTATGCATTGAATCAATTAGAAATATAATTCGACCAATTACATTAGCTATTCGATTAACAGCTAAT

TCCATCAATTTTAATACCATTTATAGTATGCATTGAATCAATTAGAAATATAATTCGACCAATTACATTAGCTATTCGATTAACAGCTAAT

TCCATCAATTTTAATACCATTTATAGTATGCATTGAATCAATTAGAAATATAATTCGACCAATTACATTAGCTATTCGATTAACAGCTAAT

4,480 4,490 4,500 4,510 4,520 4,530 4,540 4,550 4,560

ATAATTGCAGGTCATTTATTATTAACTCTTCTAGGAAATAACGGACCATCAATAAATTTATTCTTTTTAAATATTTTAATTATTTCT

TATAATTGCAGGTCATTTATTATTAACTCTTCTAGGAAATAACGGACCATCAATAAATTTATTCTTTTTAAATATTTTAATTATTTCT

TATAATTGCAGGTCATTTATTATTAACTCTTCTAGGAAATAACGGACCATCAATAAATTTATTCTTTTTAAATATTTTAATTATTTCT

TATAATTGCAGGTCATTTATTATTAACTCTTCTAGGAAATAACGGACCATCAATAAATTTATTCTTTTTAAATATTTTAATTATTTCT

TATAATTGCAGGTCATTTATTATTAACTCTTCTAGGAAATAACGGACCATCAATAAATTTATTCTTTTTAAATATTTTAATTATTTCT

4,570 4,580 4,590 4,600 4,610 4,620 4,630 4,640

TCAAATAATATTATTAACTTTAGAAAGAGCTGTTGCCATCATCCAATCCTATGTTTTTTCAGTACTAAGAACTTTATATTCT

TCAAATAATATTATTAACTTTAGAAAGAGCTGTTGCCATCATTCAATCCTATGTTTTTTCAGTACTAAGAACTTTATATTCT

TCAAATAATATTATTAACTTTAGAAAGAGCTGTTGCCATCATCCAATCCTATGTTTTTTCAGTACTAAGAACTTTATATTCT

TCAAATAATATTATTAACTTTAGAAAGAGCTGTTGCCATCATCCAATCCTATGTTTTTTCAGTACTAAGAACTTTATATTCT

TCAAATAATATTATTAACTTTAGAAAGAGCTGTTGCCATCATCCAATCCTATGTTTTTTCAGTACTAAGAACTTTATATTCT

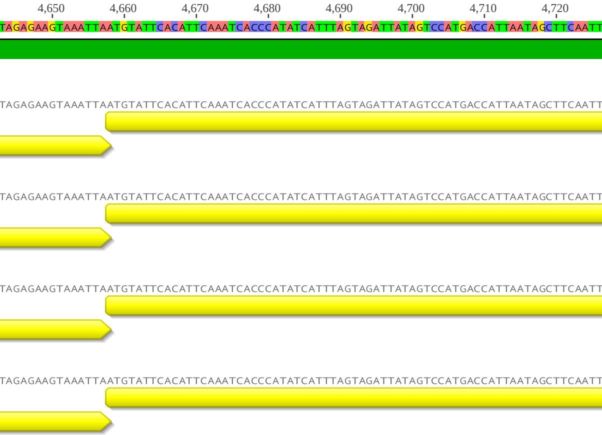
4,650 4,660 4,670 4,680 4,690 4,700 4,710 4,720
TAGAGAAGTAAATTAATGTATTCACATTCAAATCACCCATATCATTTAGTAGATTATAGTCCATGACCATTAATAGCTTCAATT
TAGAGAAGTAAATTAATGTATTCACATTCAAATCACCCATATCATTTAGTAGATTATAGTCCATGACCATTAATAGCTTCAATT
TAGAGAAGTAAATTAATGTATTCACATTCAAATCACCCATATCATTTAGTAGATTATAGTCCATGACCATTAATAGCTTCAATT
TAGAGAAGTAAATTAATGTATTCACATTCAAATCACCCATATCATTTAGTAGATTATAGTCCATGACCATTAATAGCTTCAATT
TAGAGAAGTAAATTAATGTATTCACATTCAAATCACCCATATCATTTAGTAGATTATAGTCCATGACCATTAATAGCTTCAATT

4,730 4,740 4,750 4,760 4,770 4,780 4,790 4,800 4,810

GGAATTATAACTTCAGTTTCAGGTCTTGTAAAATGATTCCATTATTATGAAATAAATTTATTTTTATTAGGTAACTTAATTTTATTAATT

GGAATTATAACTTCAGTTTCAGGTCTTGTAAAATGATTCCATTATTATGAAATAAATTTATTTTTATTAGGTAACTTAATTTTATTAATT

GGAATTATAACTTCAGTTTCAGGTCTTGTAAAATGATTCCATTATTATGAAATAAATTTATTTTTATTAGGTAACTTAATTTTATTAATT

GGAATTATAACTTCAGTTTCAGGTCTTGTAAAATGATTCCATTATTATGAAATAAATTTATTTTTATTAGGTAACTTAATTTTATTAATT

GGAATTATAACTTCAGTTTCAGGTCTTGTAAAATGATTCCATTATTATGAAATAAATTTATTTTTATTAGGTAACTTAATTTTATTAATT

4,820 4,830 4,840 4,850 4,860 4,870 4,880 4,890

ACTATTTTTCAATGATGACGAGATGTAATTCGAGAAAGAACATTTCAAGGAATACATACTCAAATTGTAATTAACAGATT

TACTATTTTTCAATGATGACGAGATGTAATTCGAGAAAGAACATTTCAAGGAATACATACTCAAATTGTAATTAACAGATT

TACTATTTTTCAATGATGACGAGATGTAATTCGAGAAAGAACATTTCAAGGAATACATACTCAAATTGTAATTAACAGATT

TACTATTTTTCAATGATGACGAGATGTAATTCGAGAAAGAACATTTCAAGGAATACATACTCAAATTGTAATTAACAGATT

TACTATTTTTCAATGATGACGAGATGTAATTCGAGAAAGAACATTTCAAGGAATACATACTCAAATTGTAATTAACAGATT

4,900 4,910 4,920 4,930 4,940 4,950 4,960 4,970 4,980

TAAAATATGGAATAATTCTATTTATTATTTCTGAAATTATATTCTTTATTTCCTTTTTTTGAGCTTTTTTTCATAGAAGACTTGCCCCAT

TAAAATATGGAATAATTCTATTTATTATTTCTGAAATTATATTCTTTATTTCCTTTTTTTGAGCTTTTTTTCATAGAAGACTTGCCCCAT

TAAAATATGGAATAATTCTATTTATTATTTCTGAAATTATATTCTTTATTTCCTTTTTTTGAGCTTTTTTTCATAGAAGACTTGCCCCAT

TAAAATATGGAATAATTCTATTTATTATTTCTGAAATTATATTCTTTATTTCCTTTTTTTGAGCTTTTTTTCATAGAAGACTTGCCCCAT

TAAAATATGGAATAATTCTATTTATTATTTCTGAAATTATATTCTTTATTTCCTTTTTTTGAGCTTTTTTTCATAGAAGACTTGCCCCAT

4,990 5,000 5,010 5,020 5,030 5,040 5,050 5,060 5,070

TCAATTGAAACAGGATTAATTTGACCTCCTAAAGGAATTATTCCATTTAATCCTATACAAATTCCACTATTAAATACAATAATTCTTTTAT

TCAATTGAAACAGGATTAATTTGACCTCCTAAAGGAATTATTCCATTTAATCCTATACAAATTCCACTATTAAATACAATAATTCTTTTAT

COX3 CDS

TCAATTGAAACAGGATTAATTTGACCTCCTAAAGGAATTATTCCATTTAATCCTATACAAATTCCACTATTAAATACAATAATTCTTTTAT

COX3 CDS

TCAATTGAAACAGGATTAATTTGACCTCCTAAAGGAATTATTCCATTTAATCCTATACAAATTCCACTATTAAATACAATAATTCTTTTAT

COX3 CDS

TCAATTGAAACAGGATTAATTTGACCTCCTAAAGGAATTATTCCATTTAATCCTATACAAATTCCACTATTAAATACAATAATTCTTTTAT

COX3 CDS

5,080 5,090 5,100 5,110 5,120 5,130 5,140 5,150 5,160

CTTCAGGTATTACTATCACTTGAGCTCATCATAGAATTATAAATAATAATTATACTCAAATAACTCAAGGATTATCCTTTACAATTC

CTTCAGGTATTACTATCACTTGAGCTCATCATAGAATTATAAATAATAATTATACTCAAATAACTCAAGGATTATCCTTTACAATTC

CTTCAGGTATTACTATCACTTGAGCTCATCATAGAATTATAAATAATAATTATACTCAAATAACTCAAGGATTATCCTTTACAATTC

CTTCAGGTATTACTATCACTTGAGCTCATCATAGAATTATAAATAATAATTATACTCAAATAACTCAAGGATTATCCTTTACAATTC

CTTCAGGTATTACTATCACTTGAGCTCATCATAGAATTATAAATAATAATTATACTCAAATAACTCAAGGATTATCCTTTACAATTC

5,170 5,180 5,190 5,200 5,210 5,220 5,230 5,240

TTTTAGGTATTTATTTTTCTATACTTCAAATATATGAATATATTGAATCTAAATTTACAATTGCAGATTCAATTTATGGATC

TTTTAGGTATTTATTTTTCTATACTTCAAATATATGAATATATTGAATCTAAATTTACAATTGCAGATTCAATTTATGGATC

TTTTAGGTATTTATTTTTCTATACTTCAAATATATGAATATATTGAATCTAAATTTACAATTGCAGATTCAATTTATGGATC

TTTTAGGTATTTATTTTTCTATACTTCAAATATATGAATATATTGAATCTAAATTTACAATTGCAGATTCAATTTATGGATC

TTTTAGGTATTTATTTTTCTATACTTCAAATATATGAATATATTGAATCTAAATTTACAATTGCAGATTCAATTTATGGATC

5,250 5,260 5,270 5,280 5,290 5,300 5,310 5,320 5,330

TACATTTTTCATAACAACAGGTTTCCATGGAATTCATGTTCTTATTGGAACATCATTTTTATTAATCTGTTTAATTCGACATATAAATTTT

TACATTTTTCATAACAACAGGTTTCCATGGAATTCATGTTCTTATTGGAACATCATTTTTATTAATCTGTTTAATTCGACATATAAATTTT

TACATTTTTCATAACAACAGGTTTCCATGGAATTCATGTTCTTATTGGAACATCATTTTTATTAATCTGTTTAATTCGACATATAAATTTT

TACATTTTTCATAACAACAGGTTTCCATGGAATTCATGTTCTTATTGGAACATCATTTTTATTAATCTGTTTAATTCGACATATAAATTTT

TACATTTTTCATAACAACAGGTTTCCATGGAATTCATGTTCTTATTGGAACATCATTTTTATTAATCTGTTTAATTCGACATATAAATTTT

5,340 5,350 5,360 5,370 5,380 5,390 5,400 5,410 5,420

CACTTCTCAAAAAATCATCACTTTGGATTTGAAGCTGCAGCTTGATATTGACATTTTGTTGATGTAATTTGATTATTTCTTTTTATC

CACTTCTCAAAAAATCATCACTTTGGATTTGAAGCTGCAGCTTGATATTGACATTTTGTTGATGTAATTTGATTATTTCTTTTTATC

CACTTCTCAAAAAATCATCACTTTGGATTTGAAGCTGCAGCTTGATATTGACATTTTGTTGATGTAATTTGATTATTTCTTTTTATC

CACTTCTCAAAAAATCATCACTTTGGATTTGAAGCTGCAGCTTGATATTGACATTTTGTTGATGTAATTTGATTATTTCTTTTTATC

CACTTCTCAAAAAATCATCACTTTGGATTTGAAGCTGCAGCTTGATATTGACATTTTGTTGATGTAATTTGATTATTTCTTTTTATC

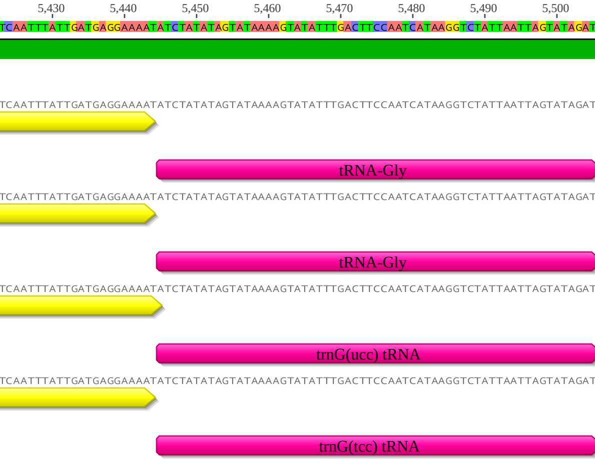
5,430
5,440
5,450
5,460
5,470
5,480
5,490
5,500
TCAATTTATTGATGAGGAAAATATCTATATAGTATAAAAGTATATTTGACTTCCAATCATAAGGTCTATTAATTAGTATAGAT
TCAATTTATTGATGAGGAAAATATCTATATAGTATAAAAGTATATTTGACTTCCAATCATAAGGTCTATTAATTAGTATAGAT
tRNA-Gly
TCAATTTATTGATGAGGAAAATATCTATATAGTATAAAAGTATATTTGACTTCCAATCATAAGGTCTATTAATTAGTATAGAT
tRNA-Gly
TCAATTTATTGATGAGGAAAATATCTATATAGTATAAAAGTATATTTGACTTCCAATCATAAGGTCTATTAATTAGTATAGAT
trnG(ucc) tRNA
TCAATTTATTGATGAGGAAAATATCTATATAGTATAAAAGTATATTTGACTTCCAATCATAAGGTCTATTAATTAGTATAGAT
trnG(tcc) tRNA

5,510 5,520 5,530 5,540 5,550 5,560 5,570 5,580 5,590

AATTTATTCAATTTTTATAATTAGATTAATTTTAATTTCTATTTCAATAACAATTATATTTATTTCTTCACTATTATCAAAAAAATCAAT

AATTTATTCAATTTTTATAATTAGATTAATTTTAATTTCTATTTCAATAACAATTATATTTATTTCTTCACTATTATCAAAAAAATCAAT

AATTTATTCAATTTTTATAATTAGATTAATTTTAATTTCTATTTCAATAACAATTATATTTATTTCTTCACTATTATCAAAAAAATCAAT

AATTTATTCAATTTTTATAATTAGATTAATTTTAATTTCTATTTCAATAACAATTATATTTATTTCTTCACTATTATCAAAAAAATCAAT

AATTTATTCAATTTTTATAATTAGATTAATTTTAATTTCTATTTCAATAACAATTATATTTATTTCTTCACTATTATCAAAAAAATCAAT

5,600 5,610 5,620 5,630 5,640 5,650 5,660 5,670

CATTGATCGAGAAAAAAGATCTCCTTTTGAATGTGGATTTGATCCAAAATCTTCTTCTCGAATACCTTTCTCTTTACGATT

CATTGATCGAGAAAAAAGATCTCCTTTTGAATGTGGATTTGATCCAAAATCTTCTTCTCGAATACCTTTCTCTTTACGATT

CATTGATCGAGAAAAAAGATCTCCTTTTGAATGTGGATTTGATCCAAAATCTTCTTCTCGAATACCTTTCTCTTTACGATT

CATTGATCGAGAAAAAAGATCTCCTTTTGAATGTGGATTTGATCCAAAATCTTCTTCTCGAATACCTTTCTCTTTACGATT

CATTGATCGAGAAAAAAGATCTCCTTTTGAATGTGGATTTGATCCAAAATCTTCTTCTCGAATACCTTTCTCTTTACGATT

5,680 5,690 5,700 5,710 5,720 5,730 5,740 5,750

TTTTTTAATTACAATTATTTTTTTAATTTTTGATGTAGAAATTGCATTACTATTACCCATTATAATAATCTATAAAATTT

TTTTTTAATTACAATTATTTTTTTAATTTTTGATGTAGAAATTGCATTACTATTACCCATTATAATAATCTATAAAATTT

ND3 CDS

TTTTTTAATTACAATTATTTTTTTAATTTTTGATGTAGAAATTGCATTACTATTACCCATTATAATAATCTATAAAATTT

ND3 CDS

TTTTTTAATTACAATTATTTTTTTAATTTTTGATGTAGAAATTGCATTACTATTACCCATTATAATAATCTATAAAATTT

ND3 CDS

TTTTTTAATTACAATTATTTTTTTAATTTTTGATGTAGAAATTGCATTACTATTACCCATTATAATAATCTATAAAATTT

ND3 CDS

5,760 5,770 5,780 5,790 5,800 5,810 5,820 5,830 5,840

CAAATTTAACAACTTGAATCTTTACATCTATTTTATTTATTATTATTTTACTAATAGGATTATATCATGAATGAAATCAAGGAATATT

CAAATTTAACAACTTGAATCTTTACATCTATTTTATTTATTATTATTTTACTAATAGGATTATATCATGAATGAAATCAAGGAATATT

CAAATTTAACAACTTGAATCTTTACATCTATTTTATTTATTATTATTTTACTAATAGGATTATATCATGAATGAAATCAAGGAATATT

CAAATTTAACAACTTGAATCTTTACATCTATTTTATTTATTATTATTTTACTAATAGGATTATATCATGAATGAAATCAAGGAATATT

CAAATTTAACAACTTGAATCTTTACATCTATTTTATTTATTATTATTTTACTAATAGGATTATATCATGAATGAAATCAAGGAATATT

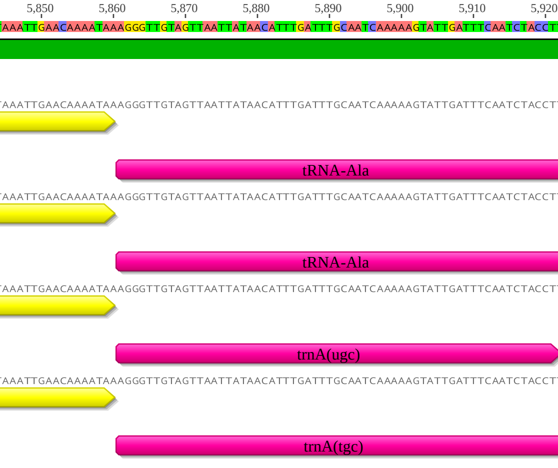

5,850
5,860
5,870
5,880
5,890
5,900
5,910
5,920
AAATTGAACAAAATAAAGGGTTGTAGTTAATTATAACATTTGATTTGCAATCAAAAAGTATTGATTTCAATCTACC
AAATTGAACAAAATAAAGGGTTGTAGTTAATTATAACATTTGATTTGCAATCAAAAAGTATTGATTTCAATCTACCT
tRNA-Ala
AAATTGAACAAAATAAAGGGTTGTAGTTAATTATAACATTTGATTTGCAATCAAAAAGTATTGATTTCAATCTACCT
tRNA-Ala
AAATTGAACAAAATAAAGGGTTGTAGTTAATTATAACATTTGATTTGCAATCAAAAAGTATTGATTTCAATCTACCT
trnA(ugc)
AAATTGAACAAAATAAAGGGTTGTAGTTAATTATAACATTTGATTTGCAATCAAAAAGTATTGATTTCAATCTACCT
trnA(tgc)

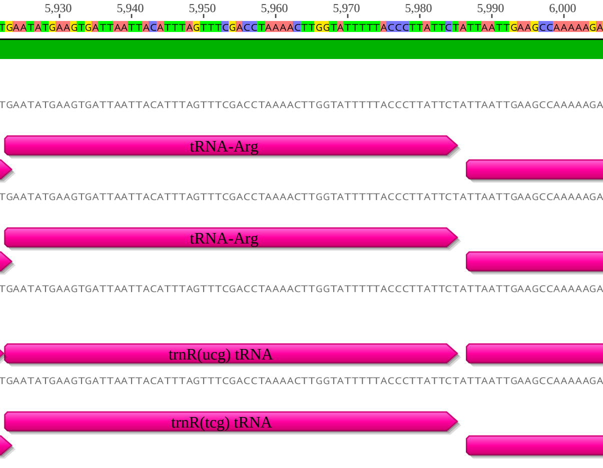

5,930
5,940
5,950
5,960
5,970
5,980
5,990
6,000
TGAATATGAAGTGATTAATTACATTTAGTTTCGACCTAAAACTTGGTATTTTTACCCTTATTCTATTAATTGAAGCCAAAAAGA
TGAATATGAAGTGATTAATTACATTTAGTTTCGACCTAAAACTTGGTATTTTTACCCTTATTCTATTAATTGAAGCCAAAAAGA
tRNA-Arg
TGAATATGAAGTGATTAATTACATTTAGTTTCGACCTAAAACTTGGTATTTTTACCCTTATTCTATTAATTGAAGCCAAAAAGA
tRNA-Arg
TGAATATGAAGTGATTAATTACATTTAGTTTCGACCTAAAACTTGGTATTTTTACCCTTATTCTATTAATTGAAGCCAAAAAGA
trnR(ucg) tRNA
TGAATATGAAGTGATTAATTACATTTAGTTTCGACCTAAAACTTGGTATTTTTACCCTTATTCTATTAATTGAAGCCAAAAAGA
trnR(tcg) tRNA

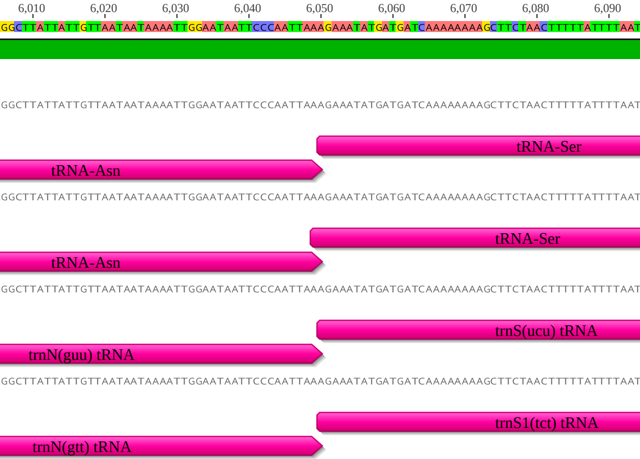

6,010
6,020
6,030
6,040
6,050
6,060
6,070
6,080
6,090
GGCTTATTATTGTTAATAATAAAATTGGAATAATTCCCAATTAAAGAAATATGATGATCAAAAAAAAGCTTCTAACTTTTTATTTTAAT
tRNA-Ser
tRNA-Asn
GGCTTATTATTGTTAATAATAAAATTGGAATAATTCCCAATTAAAGAAATATGATGATCAAAAAAAAGCTTCTAACTTTTTATTTTAAT
tRNA-Ser
tRNA-Asn
GGCTTATTATTGTTAATAATAAAATTGGAATAATTCCCAATTAAAGAAATATGATGATCAAAAAAAAGCTTCTAACTTTTTATTTTAAT
trnS(ucu) tRNA
trnN(guu) tRNA
GGCTTATTATTGTTAATAATAAAATTGGAATAATTCCCAATTAAAGAAATATGATGATCAAAAAAAAGCTTCTAACTTTTTATTTTAAT
trnS1(tct) tRNA
trnN(gtt) tRNA

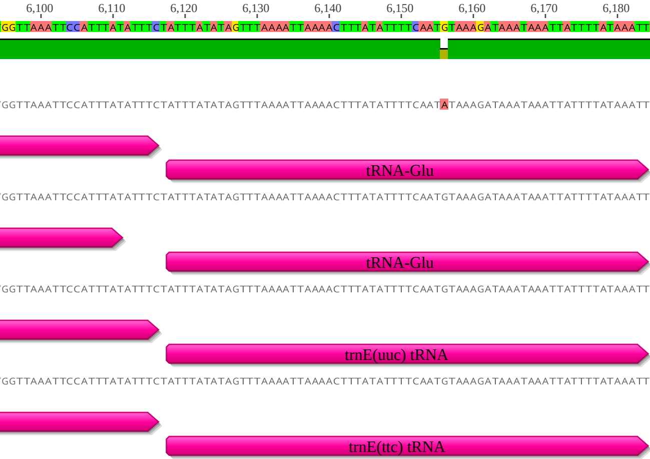
6,100
6,110
6,120
6,130
6,140
6,150
6,160
6,170
6,180
GGTTAAATTCCATTTATATTTCTATTTATATAGTTTAAAATTAAAACTTTATATTTTCAATGTAAAGATAAATAAATTATTTTATAAATT
GGTTAAATTCCATTTATATTTCTATTTATATAGTTTAAAATTAAAACTTTATATTTTCAATATAAAGATAAATAAATTATTTTATAAATT
tRNA-Glu
GGTTAAATTCCATTTATATTTCTATTTATATAGTTTAAAATTAAAACTTTATATTTTCAATGTAAAGATAAATAAATTATTTTATAAATT
tRNA-Glu
GGTTAAATTCCATTTATATTTCTATTTATATAGTTTAAAATTAAAACTTTATATTTTCAATGTAAAGATAAATAAATTATTTTATAAATT
trnE(uuc) tRNA
GGTTAAATTCCATTTATATTTCTATTTATATAGTTTAAAATTAAAACTTTATATTTTCAATGTAAAGATAAATAAATTATTTTATAAATT
trnE(ttc) tRNA

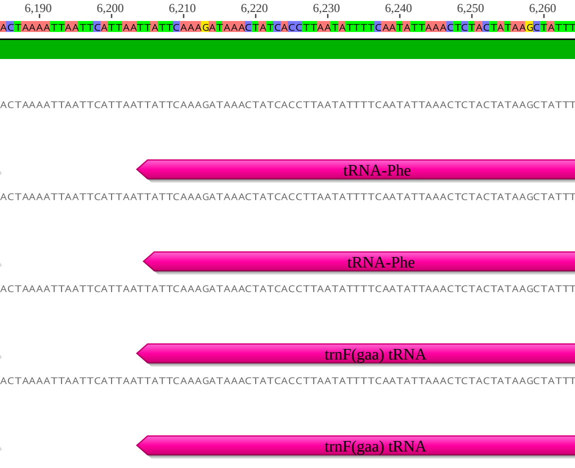
6,190
6,200
6,210
6,220
6,230
6,240
6,250
6,260
ACTAAAATTAATTCATTAATTATTCAAAGATAAACTATCACCTTAATATTTTCAATATTAAACTCTACTATAAGCTATTT
ACTAAAATTAATTCATTAATTATTCAAAGATAAACTATCACCTTAATATTTTCAATATTAAACTCTACTATAAGCTATTT
tRNA-Phe
ACTAAAATTAATTCATTAATTATTCAAAGATAAACTATCACCTTAATATTTTCAATATTAAACTCTACTATAAGCTATTT
tRNA-Phe
ACTAAAATTAATTCATTAATTATTCAAAGATAAACTATCACCTTAATATTTTCAATATTAAACTCTACTATAAGCTATTT
trnF(gaa) tRNA
ACTAAAATTAATTCATTAATTATTCAAAGATAAACTATCACCTTAATATTTTCAATATTAAACTCTACTATAAGCTATTT
trnF(gaa) tRNA

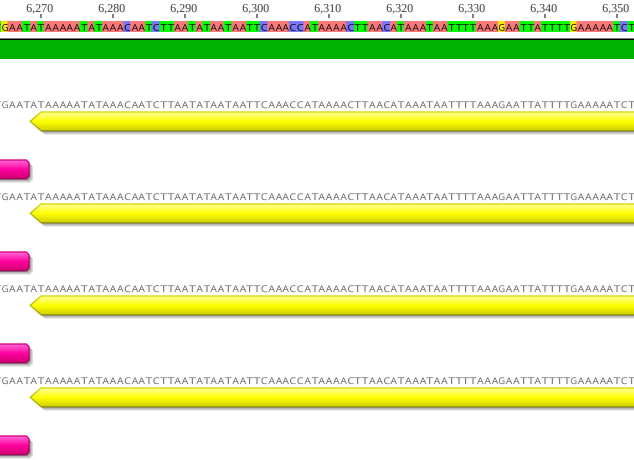
6,270
6,280
6,290
6,300
6,310
6,320
6,330
6,340
6,350
GAATATAAAAATATAAACAATCTTAATATAATAATTCAAACCATAAAACTTAACATAAATAATTTTAAAGAATTATTTTGAAAAATCT
GAATATAAAAATATAAACAATCTTAATATAATAATTCAAACCATAAAACTTAACATAAATAATTTTAAAGAATTATTTTGAAAAATCT
GAATATAAAAATATAAACAATCTTAATATAATAATTCAAACCATAAAACTTAACATAAATAATTTTAAAGAATTATTTTGAAAAATCT
GAATATAAAAATATAAACAATCTTAATATAATAATTCAAACCATAAAACTTAACATAAATAATTTTAAAGAATTATTTTGAAAAATCT
GAATATAAAAATATAAACAATCTTAATATAATAATTCAAACCATAAAACTTAACATAAATAATTTTAAAGAATTATTTTGAAAAATCT

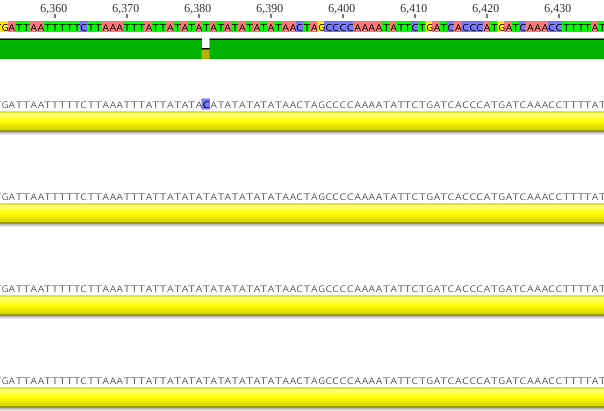
6,360 6,370 6,380 6,390 6,400 6,410 6,420 6,430
TGATTAATTTTTCTTAAATTTATTATATATATATATATATATAACTAGCCCCAAAATATTCTGATCACCCATGATCAAACCTTTTAT
TGATTAATTTTTCTTAAATTTATTATATACATATATATATAACTAGCCCCAAAATATTCTGATCACCCATGATCAAACCTTTTAT
TGATTAATTTTTCTTAAATTTATTATATATATATATATATATAACTAGCCCCAAAATATTCTGATCACCCATGATCAAACCTTTTAT
TGATTAATTTTTCTTAAATTTATTATATATATATATATATATAACTAGCCCCAAAATATTCTGATCACCCATGATCAAACCTTTTAT
TGATTAATTTTTCTTAAATTTATTATATATATATATATATATAACTAGCCCCAAAATATTCTGATCACCCATGATCAAACCTTTTAT

6,440 6,450 6,460 6,470 6,480 6,490 6,500 6,510 6,520

TAAACTAATTTACCAATTATTAAAGGAAAATATATAATTCCATAAGTTCTAATATAAGGTAAAAATCACATTGAACTAAAAAAAAAT

TAAACTAATTTACCAATTATTAAAGGAAAATATATAATTCCATAAGTTCTAATATAAGGTAAAAATCACATTGAACTAAAAAAAAAT

TAAACTAATTTACCAATTATTAAAGGAAAATATATAATTCCATAAGTTCTAATATAAGGTAAAAATCACATTGAACTAAAAAAAAAT

TAAACTAATTTACCAATTATTAAAGGAAAATATATAATTCCATAAGTTCTAATATAAGGTAAAAATCACATTGAACTAAAAAAAAAT

TAAACTAATTTACCAATTATTAAAGGAAAATATATAATTCCATAAGTTCTAATATAAGGTAAAAATCACATTGAACTAAAAAAAAAT

6,530 6,540 6,550 6,560 6,570 6,580 6,590

TCTTAAACTATAAAACTTTAAAGTAATACTTTTTTTATATAAAGAAATGTTTGATAACAATAAACCAAAAATTAT

TCTTAAACTATAAAACTTTAAAGTAATACTTTTTTTATATAAAGAAATGTTTGATAACAATAAACCAAAAATTAT

TCTTAAACTATAAAACTTTAAAGTAATACTTTTTTTATATAAAGAAATGTTTGATAACAATAAACCAAAAATTAT

TCTTAAACTATAAAACTTTAAAGTAATACTTTTTTTATATAAAGAAATGTTTGATAACAATAAACCAAAAATTAT

TCTTAAACTATAAAACTTTAAAGTAATACTTTTTTTATATAAAGAAATGTTTGATAACAATAAACCAAAAATTAT

6,600 6,610 6,620 6,630 6,640 6,650 6,660 6,670

TCCTATAAAACAAATAAATAAAACTAACATTTTCAAATAAAAAGGTAAAATAATAATATTAATTTTAGAAAATAATAAT

TCCTATAAAACAAATAAATAAAACTAACATTTTCAAATAAAAAGGTAAAATAATAATATTAATTTTAGAAAATAATAAT

TCCTATAAAACAAATAAATAAAACTAACATTTTCAAATAAAAAGGTAAAATAATAATATTAATTTTAGAAAATAATAAT

TCCTATAAAACAAATAAATAAAACTAACATTTTCAAATAAAAAGGTAAAATAATAATATTAATTTTAGAAAATAATAAT

TCCTATAAAACAAATAAATAAAACTAACATTTTCAAATAAAAAGGTAAAATAATAATATTAATTTTAGAAAATAATAAT

6,680 6,690 6,700 6,710 6,720 6,730 6,740 6,750

CAACTTAATATACTACCACTAAAAATTCTAAATAACATTAATGTTAATATTCTCTTTAATATAACTCAACCTTCATCATTT

CAACTTAATATACTACCACTAAAAATTCTAAATAACATTAATGTTAATATTCTCTTTAATATAACTCAACCTTCATCATTT

CAACTTAATATACTACCACTAAAAATTCTAAATAACATTAATGTTAATATTCTCTTTAATATAACTCAACCTTCATCATTT

CAACTTAATATACTACCACTAAAAATTCTAAATAACATTAATGTTAATATTCTCTTTAATATAACTCAACCTTCATCATTT

CAACTTAATATACTACCACTAAAAATTCTAAATAACATTAATGTTAATATTCTCTTTAATATAACTCAACCTTCATCATTT

6,760 6,770 6,780 6,790 6,800 6,810 6,820 6,830

AATAAATTTAAAGAAAAAAAATTATAATTTCCTAATATAGTATAAAATACTAAACGAAATGAATAACATACTGTTAAACC

AATAAATTTAAAGAAAAAAAATTATAATTTCCTAATATAGTATAAAATACTAAACGAAATGAATAACATACTGTTAAACC

AATAAATTTAAAGAAAAAAAATTATAATTTCCTAATATAGTATAAAATACTAAACGAAATGAATAACATACTGTTAAACC

AATAAATTTAAAGAAAAAAAATTATAATTTCCTAATATAGTATAAAATACTAAACGAAATGAATAACATACTGTTAAACC

AATAAATTTAAAGAAAAAAAATTATAATTTCCTAATATAGTATAAAATACTAAACGAAATGAATAACATACTGTTAAACC

6,840 6,850 6,860 6,870 6,880 6,890 6,900 6,910

TGTAGAAAATACAAATAAAAACATAATAAATAAATTAACATAACTTATTATTACTATTTCTAAAATTATATCTTTT

TGTAGAAAATACAAATAAAAACATAATAAATAAATTAACATAACTTATTATTACTATTTCTAAAATTATATCTTTT

TGTAGAAAATACAAATAAAAACATAATAAATAAATTAACATAACTTATTATTACTATTTCTAAAATTATATCTTTT

TGTAGAAAATACAAATAAAAACATAATAAATAAATTAACATAACTTATTATTACTATTTCTAAAATTATATCTTTT

TGTAGAAAATACAAATAAAAACATAATAAATAAATTAACATAACTTATTATTACTATTTCTAAAATTATATCTTTT

6,920 6,930 6,940 6,950 6,960 6,970 6,980 6,990

TGAATAAAATCCAGATAAAAAAGGTATTCCACATAAAGATAAATTAGCTACATTAAAACAAGAAGAAGTAATTGGTATTATTAAT

TGAATAAAATCCAGATAAAAAAGGTATTCCACATAAAGATAAATTAGCTACATTAAAACAAGAAGAAGTAATTGGTATTATTAAT

TGAATAAAATCCAGATAAAAAAGGTATTCCACATAAAGATAAATTAGCTACATTAAAACAAGAAGAAGTAATTGGTATTATTAAT

TGAATAAAATCCAGATAAAAAAGGTATTCCACATAAAGATAAATTAGCTACATTAAAACAAGAAGAAGTAATTGGTATTATTAAT

TGAATAAAATCCAGATAAAAAAGGTATTCCACATAAAGATAAATTAGCTACATTAAAACAAGAAGAAGTAATTGGTATTATTAAT

7,000 7,010 7,020 7,030 7,040 7,050 7,060 7,070

CTTAATCTTCCTATTATTCGAATATCTTGACAATTACTTATATTATGAATAATAGAACCTGCACATAAAAATAACAAAGC

CTTAATCTTCCTATTATTCGAATATCTTGACAATTACTTATATTATGAATAATAGAACCTGCACATAAAAATAACAAAGC

CTTAATCTTCCTATTATTCGAATATCTTGACAATTACTTATATTATGAATAATAGAACCTGCACATAAAAATAACAAAGC

CTTAATCTTCCTATTATTCGAATATCTTGACAATTACTTATATTATGAATAATAGAACCTGCACATAAAAATAACAAAGC

CTTAATCTTCCTATTATTCGAATATCTTGACAATTACTTATATTATGAATAATAGAACCTGCACATAAAAATAACAAAGC

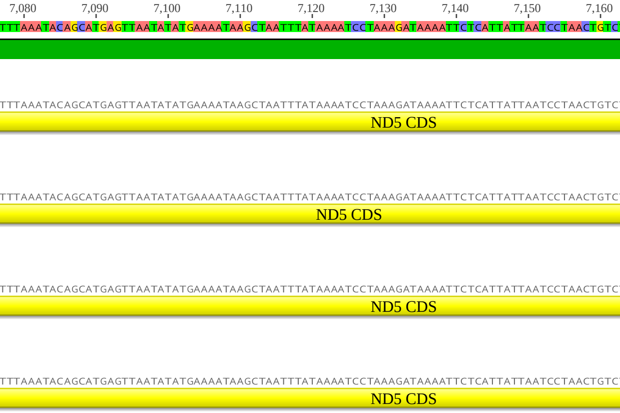

7,080
7,090
7,100
7,110
7,120
7,130
7,140
7,150
7,160
TTTAAATACAGCATGAGTTAATATATGAAAATAAGCTAATTTATAAAATCCTAAAGATAAAATTCTCATTATTAATCCTAACTGTC
TTTAAATACAGCATGAGTTAATATATGAAAATAAGCTAATTTATAAAATCCTAAAGATAAAATTCTCATTATTAATCCTAACTGTC
ND5 CDS
TTTAAATACAGCATGAGTTAATATATGAAAATAAGCTAATTTATAAAATCCTAAAGATAAAATTCTCATTATTAATCCTAACTGTC
ND5 CDS
TTTAAATACAGCATGAGTTAATATATGAAAATAAGCTAATTTATAAAATCCTAAAGATAAAATTCTCATTATTAATCCTAACTGTC
ND5 CDS
TTTAAATACAGCATGAGTTAATATATGAAAATAAGCTAATTTATAAAATCCTAAAGATAAAATTCTCATTATTAATCCTAACTGTC
ND5 CDS

7,170 7,180 7,190 7,200 7,210 7,220 7,230 7,240

TTAAAGTTGATAATGCAATAATTTTCTTTAAATCAAATTCAAAATTAGCACATAAACCTGCTATAAATATAGTTAAACTAGAAAT

TTAAAGTTGATAATGCAATAATTTTCTTTAAATCAAATTCAAAATTAGCACATAAACCTGCTATAAATATAGTTAAACTAGAAAT

TTAAAGTTGATAATGCAATAATTTTCTTTAAATCAAATTCAAAATTAGCACATAAACCTGCTATAAATATAGTTAAACTAGAAAT

TTAAAGTTGATAATGCAATAATTTTCTTTAAATCAAATTCAAAATTAGCACATAAACCTGCTATAAATATAGTTAAACTAGAAAT

TTAAAGTTGATAATGCAATAATTTTCTTTAAATCAAATTCAAAATTAGCACATAAACCTGCTATAAATATAGTTAAACTAGAAAT

7,250 7,260 7,270 7,280 7,290 7,300 7,310 7,320 7,330

AATCAATAAAACTGAAGATCACTCTTCTAATACAAATCTAAATCGAATTAATAAATAAATTCCAGCAGTTACCAAAGTAGATGAATGAAC

TAATCAATAAAACTGAAGATCACTCTTCTAATACAAATCTAAATCGAATTAATAAATAAATTCCAGCAGTTACCAAAGTAGATGAATGAAC

TAATCAATAAAACTGAAGATCACTCTTCTAATACAAATCTAAATCGAATTAATAAATAAATTCCAGCAGTTACCAAAGTAGATGAATGAAC

TAATCAATAAAACTGAAGATCACTCTTCTAATACAAATCTAAATCGAATTAATAAATAAATTCCAGCAGTTACCAAAGTAGATGAATGAAC

TAATCAATAAAACTGAAGATCACTCTTCTAATACAAATCTAAATCGAATTAATAAATAAATTCCAGCAGTTACCAAAGTAGATGAATGAAC

7,340 7,350 7,360 7,370 7,380 7,390 7,400 7,410

TAAAGCTGATACTGGTGTAGGAGCTGCTATTGCAGCAGGTAATCATGAAGAAAAAGGAATTTGAGCACTTTTTGTTAAT

TAAAGCTGATACTGGTGTAGGAGCTGCTATTGCAGCAGGTAATCATGAAGAAAAAGGAATTTGAGCACTTTTTGTTAAT

TAAAGCTGATACTGGTGTAGGAGCTGCTATTGCAGCAGGTAATCATGAAGAAAAAGGAATTTGAGCACTTTTTGTTAAT

TAAAGCTGATACTGGTGTAGGAGCTGCTATTGCAGCAGGTAATCATGAAGAAAAAGGAATTTGAGCACTTTTTGTTAAT

TAAAGCTGATACTGGTGTAGGAGCTGCTATTGCAGCAGGTAATCATGAAGAAAAAGGAATTTGAGCACTTTTTGTTAAT

7,420 7,430 7,440 7,450 7,460 7,470 7,480 7,490

CTAGCTAAAATAATTATTAAACTAATTAACTTTATTTCACAACATTCCTTTATTACCTCTAAATAAAAAATATAATTTT

CTAGCTAAAATAATTATTAAACTAATTAACTTTATTTCACAACATTCCTTTATTACCTCTAAATAAAAAATATAATTTT

CTAGCTAAAATAATTATTAAACTAATTAACTTTATTTCACAACATTCCTTTATTACCTCTAAATAAAAAATATAATTTT

CTAGCTAAAATAATTATTAAACTAATTAACTTTATTTCACAACATTCCTTTATTACCTCTAAATAAAAAATATAATTTT

CTAGCTAAAATAATTATTAAACTAATTAACTTTATTTCACAACATTCCTTTATTACCTCTAAATAAAAAATATAATTTT

7,500 7,510 7,520 7,530 7,540 7,550 7,560 7,570

CAACTCCCAAAATTTAATATTCATGAAATTGCTAATAATAACATAACATCTCCTACACGATTTGATAATACAGTTAATAT

TCAACTCCCAAAATTTAATATTCATGAAATTGCTAATAATAACATAACATCTCCTACACGATTTGATAATACAGTTAATAT

TCAACTCCCAAAATTTAATATTCATGAAATTGCTAATAATAACATAACATCTCCTACACGATTTGATAATACAGTTAATAT

TCAACTCCCAAAATTTAATATTCATGAAATTGCTAATAATAACATAACATCTCCTACACGATTTGATAATACAGTTAATAT

TCAACTCCCAAAATTTAATATTCATGAAATTGCTAATAATAACATAACATCTCCTACACGATTTGATAATACAGTTAATAT

7,580 7,590 7,600 7,610 7,620 7,630 7,640 7,650 7,660

CCCAGCATTTAAACTTTTAATATTTTGAAAATAAATTACTAAACAATAAGAAATTAACCCTAATCCATCTCAACCTAATAAAATTCTAAT

TCCCAGCATTTAAACTTTTAATATTTTGAAAATAAATTACTAAACAATAAGAAATTAACCCTAATCCATCTCAACCTAATAAAATTCTAAT

TCCCAGCATTTAAACTTTTAATATTTTGAAAATAAATTACTAAACAATAAGAAATTAACCCTAATCCATCTCAACCTAATAAAATTCTAAT

TCCCAGCATTTAAACTTTTAATATTTTGAAAATAAATTACTAAACAATAAGAAATTAACCCTAATCCATCTCAACCTAATAAAATTCTAAT

TCCCAGCATTTAAACTTTTAATATTTTGAAAATAAATTACTAAACAATAAGAAATTAACCCTAATCCATCTCAACCTAATAAAATTCTAAT

7,670 7,680 7,690 7,700 7,710 7,720 7,730 7,740

TAAATTAGGTCTAATAACTAATAATATTATAGAAAATACAAATATTAATATAAGTAAAATAAAACGATTAATATTTAAAT

TAAATTAGGTCTAATAACTAATAATATTATAGAAAATACAAATATTAATATAAGTAAAATAAAGCGATTAATATTTAAAT

TAAATTAGGTCTAATAACTAATAATATTATAGAAAATACAAATATTAATATAAGTAAAATAAAACGATTAATATTTAAAT

TAAATTAGGTCTAATAACTAATAATATTATAGAAAATACAAATATTAATATAAGTAAAATAAAACGATTAATATTTAAAT

TAAATTAGGTCTAATAACTAATAATATTATAGAAAATACAAATATTAATATAAGTAAAATAAAACGATTAATATTTAAAT

7,750 7,760 7,770 7,780 7,790 7,800 7,810 7,820

TCACCAATTATATATTCCATTCTATAAAAAATAACTAAACAAGAAATAAATAAAATAAAAGATATAAATAATAATCTTATT

TCACCAATTATATATTCCATTCTATAAAAAATAACTAAACAAGAAATAAATAAAATAAAAGATATAAATAATAATCTTATT

TCACCAATTATATATTCCATTCTATAAAAAATAACTAAACAAGAAATAAATAAAATAAAAGATATAAATAATAATCTTATT

TCACCAATTATATATTCCATTCTATAAAAAATAACTAAACAAGAAATAAATAAAATAAAAGATATAAATAATAATCTTATT

TCACCAATTATATATTCCATTCTATAAAAAATAACTAAACAAGAAATAAATAAAATAAAAGATATAAATAATAATCTTATT

7,830 7,840 7,850 7,860 7,870 7,880 7,890 7,900 7,910

TCAATCAAATAATAAAGTTATAACAATCCTTATTCTATTAAAAAAAAATAATTCATATTCAATAAAAATAACATAATCATAATAAATAAAAT

TCAATCAAATAATAAAGTTATAACAATCCTTATTCTATTAAAAAAAAATAATTCATATTCAATAAAAATAACATAATCATAATAAATAAAAT

TCAATCAAATAATAAAGTTATAACAATCCTTATTCTATTAAAAAAAAATAATTCATATTCAATAAAAATAACATAATCATAATAAATAAAAT

TCAATCAAATAATAAAGTTATAACAATCCTTATTCTATTAAAAAAAAATAATTCATATTCAATAAAAATAACATAATCATAATAAATAAAAT

TCAATCAAATAATAAAGTTATAACAATCCTTATTCTATTAAAAAAAAATAATTCATATTCAATAAAAATAACATAATCATAATAAATAAAAT

7,920 7,930 7,940 7,950 7,960 7,970 7,980 7,990

TAAATTCCAAATATAAAACATAATAACCTTAAAATAAATAAATTAACAAATCAAACTATACAAATTGATAAATTTAAAAT

TAAATTCCAAATATAAAACATAATAACCTTAAAATAAATAAATTAACAAATCAAACTATACAAATTGATAAATTTAAAAT

TAAATTCCAAATATAAAACATAATAACCTTAAAATAAATAAATTAACAAATCAAACTATACAAATTGATAAATTTAAAAT

TAAATTCCAAATATAAAACATAATAACCTTAAAATAAATAAATTAACAAATCAAACTATACAAATTGATAAATTTAAAAT

TAAATTCCAAATATAAAACATAATAACCTTAAAATAAATAAATTAACAAATCAAACTATACAAATTGATAAATTTAAAAT

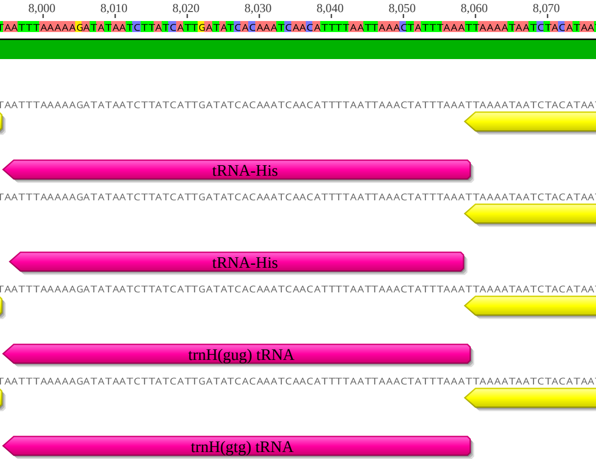
8,000
8,010
8,020
8,030
8,040
8,050
8,060
8,070
TAATTTAAAAAGATATAATCTTATCATTGATATCACAAATCAACATTTTAATTAAACTATTTAAATTAAAATAATCTACATAA
tRNA-His
tRNA-His
trnH(gug) tRNA
trnH(gtg) tRNA

8,080 8,090 8,100 8,110 8,120 8,130 8,140 8,150 8,160

TTCACTTTTTAAAACTAATAAATTTAAAGGAAATCAATGTAATAATAATAAATGAAATTCACGAATTTTTCCACTTCTAAAAGTTC

TTCACTTTTTAAAACTAATAAATTTAAAGGAAATCAATGTAATAATAATAAATGAAATTCACGAATTTTTCCACTTCTAAAAGTTC

TTCACTTTTTAAAACTAATAAATTTAAAGGAAATCAATGTAATAATAATAAATGAAATTCACGAATTTTTCCACTTCTAAAAGTTC

TTCACTTTTTAAAACTAATAAATTTAAAGGAAATCAATGTAATAATAATAAATGAAATTCACGAATTTTTCCACTTCTAAAAGTTC

TTCACTTTTTAAAACTAATAAATTTAAAGGAAATCAATGTAATAATAATAAATGAAATTCACGAATTTTTCCACTTCTAAAAGTTC

8,170 8,180 8,190 8,200 8,210 8,220 8,230 8,240

TTATTCTAATTAATACCTTTCCATGTTGACTATAAGAAAATAAATATAATGAATAAGCAACTCTAAAAAAACATATTATTCC

TTATTCTAATTAATACCTTTCCATGTTGACTATAAGAAAATAAATATAATGAATAAGCAACTCTAAAAAAACATATTATTCC

TTATTCTAATTAATACCTTTCCATGTTGACTATAAGAAAATAAATATAATGAATAAGCAACTCTAAAAAAACATATTATTCC

TTATTCTAATTAATACCTTTCCATGTTGACTATAAGAAAATAAATATAATGAATAAGCAACTCTAAAAAAACATATTATTCC

TTATTCTAATTAATACCTTTCCATGTTGACTATAAGAAAATAAATATAATGAATAAGCAACTCTAAAAAAACATATTATTCC

8,250 8,260 8,270 8,280 8,290 8,300 8,310 8,320 8,330

TAATATAAACATTCTTAATCATGATCACATTACAATTCTATTAATTAAATTAATTTCTCCTCATAAATTCAATCTAGGAGGAGCAGAT

TAATATAAACATTCTTAATCATGATCACATTACAATTCTATTAATTAAATTAATTTCTCCTCATAAATTCAATCTAGGAGGAGCAGAT

TAATATAAACATTCTTAATCATGATCACATTACAATTCTATTAATTAAATTAATTTCTCCTCATAAATTCAATCTAGGAGGAGCAGAT

TAATATAAACATTCTTAATCATGATCACATTACAATTCTATTAATTAAATTAATTTCTCCTCATAAATTCAATCTAGGAGGAGCAGAT

TAATATAAACATTCTTAATCATGATCACATTACAATTCTATTAATTAAATTAATTTCTCCTCATAAATTCAATCTAGGAGGAGCAGAT

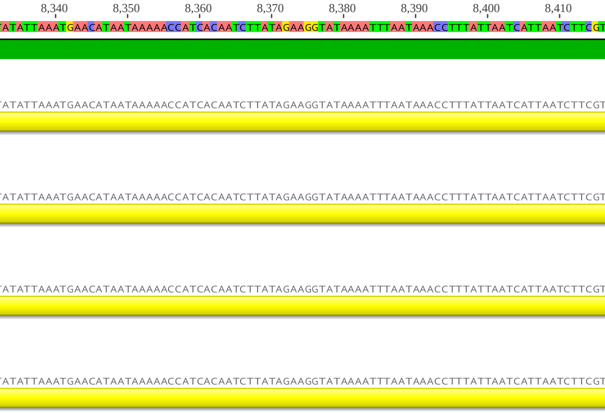

8,340
8,350
8,360
8,370
8,380
8,390
8,400
8,410
TATATTAAATGAACATAATAAAAACCATCACAATCTTATAGAAGGTATAAAATTTAATAAACCTTTATTAATCATTAATCTTCGT
TATATTAAATGAACATAATAAAAACCATCACAATCTTATAGAAGGTATAAAATTTAATAAACCTTTATTAATCATTAATCTTCGT
TATATTAAATGAACATAATAAAAACCATCACAATCTTATAGAAGGTATAAAATTTAATAAACCTTTATTAATCATTAATCTTCGT
TATATTAAATGAACATAATAAAAACCATCACAATCTTATAGAAGGTATAAAATTTAATAAACCTTTATTAATCATTAATCTTCGT
TATATTAAATGAACATAATAAAAACCATCACAATCTTATAGAAGGTATAAAATTTAATAAACCTTTATTAATCATTAATCTTCGT

8,420 8,430 8,440 8,450 8,460 8,470 8,480 8,490

CTTAAAGTTCGCTCATAAATAATATTTACTAAACAAAATAAACCTGAAGAACATAAACCATGAGCAATTATTAATAAA

CTTAAAGTTCGCTCATAAATAATATTTACTAAACAAAATAAACCTGAAGAACATAAACCATGAGCAATTATTAATAAA

CTTAAAGTTCGCTCATAAATAATATTTACTAAACAAAATAAACCTGAAGAACATAAACCATGAGCAATTATTAATAAA

CTTAAAGTTCGCTCATAAATAATATTTACTAAACAAAATAAACCTGAAGAACATAAACCATGAGCAATTATTAATAAA

CTTAAAGTTCGCTCATAAATAATATTTACTAAACAAAATAAACCTGAAGAACATAAACCATGAGCAATTATTAATAAA

8,500 8,510 8,520 8,530 8,540 8,550 8,560 8,570

TAAGATCCACATAAACCTATATATCTTATTGTTATTAAACCTACTAAAACAACTCCTATATGTGATACTGAAGAATAAGCTAC

TAAGATCCACATAAACCTATATATCTTATTGTTATTAAACCTACTAAAACAACTCCTATATGTGATACTGAAGAATAAGCTAC

TAAGATCCACATAAACCTATATATCTTATTGTTATTAAACCTACTAAAACAACTCCTATATGTGATACTGAAGAATAAGCTAC

TAAGATCCACATAAACCTATATATCTTATTGTTATTAAACCTACTAAAACAACTCCTATATGTGATACTGAAGAATAAGCTAC

TAAGATCCACATAAACCTATATATCTTATTGTTATTAAACCTACTAAAACAACTCCTATATGTGATACTGAAGAATAAGCTAC

8,580 8,590 8,600 8,610 8,620 8,630 8,640 8,650 8,660

TAAAGATTTCAAATCAGTTTGACGTAAACAAATTAAACCAACCAATACTCTACCAACCAATCTAATTCTTATTCAAATAAAATTAAAT

TAAAGATTTCAAATCAGTTTGACGTAAACAAATTAAACCAACCAATACTCTACCAACCAATCTAATTCTTATTCAAATAAAATTAAAT

TAAAGATTTCAAATCAGTTTGACGTAAACAAATTAAACCAACCAATACTCTACCAACCAATCTAATTCTTATTCAAATAAAATTAAAT

TAAAGATTTCAAATCAGTTTGACGTAAACAAATTAAACCAACCAATACTCTACCAACCAATCTAATTCTTATTCAAATAAAATTAAAT

TAAAGATTTCAAATCAGTTTGACGTAAACAAATTAAACCAACCAATACTCTACCAACCAATCTAATTCTTATTCAAATAAAATTAAAT

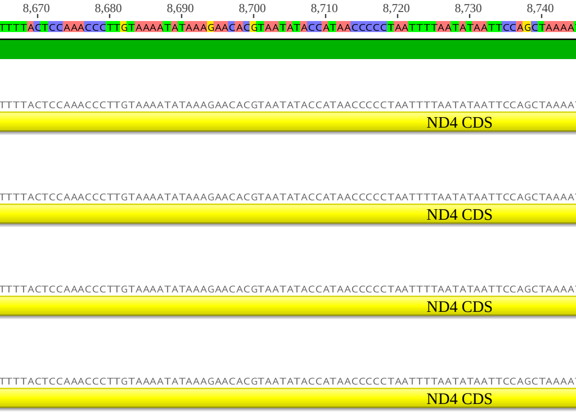

8,670
8,680
8,690
8,700
8,710
8,720
8,730
8,740
TTTTACTCCAAACCCTTGTAAAATATAAAGAACACGTAATATACCATAACCCCCTAATTTTAATATAATTCCAGCTAAAA
TTTTACTCCAAACCCTTGTAAAATATAAAGAACACGTAATATACCATAACCCCCTAATTTTAATATAATTCCAGCTAAAA
ND4 CDS
TTTTACTCCAAACCCTTGTAAAATATAAAGAACACGTAATATACCATAACCCCCTAATTTTAATATAATTCCAGCTAAAA
ND4 CDS
TTTTACTCCAAACCCTTGTAAAATATAAAGAACACGTAATATACCATAACCCCCTAATTTTAATATAATTCCAGCTAAAA
ND4 CDS
TTTTACTCCAAACCCTTGTAAAATATAAAGAACACGTAATATACCATAACCCCCTAATTTTAATATAATTCCAGCTAAAA
ND4 CDS

8,750 8,760 8,770 8,780 8,790 8,800 8,810 8,820 8,830

TTATAGACCCAGAAATAGGAGCTTCAACATGAGCTTTAGGTAATCATAAATGTAATAAAAATATTGGTATTTTTACTAAAAACGAAAAT

TTATAGACCCAGAAATAGGAGCTTCAACATGAGCTTTAGGTAATCATAAATGTAATAAAAATATTGGTATTTTTACTAAAAACGAAAAT

TTATAGACCCAGAAATAGGAGCTTCAACATGAGCTTTAGGTAATCATAAATGTAATAAAAATATTGGTATTTTTACTAAAAACGAAAAT

TTATAGACCCAGAAATAGGAGCTTCAACATGAGCTTTAGGTAATCATAAATGTAATAAAAATATTGGTATTTTTACTAAAAACGAAAAT

TTATAGACCCAGAAATAGGAGCTTCAACATGAGCTTTAGGTAATCATAAATGTAATAAAAATATTGGTATTTTTACTAAAAACGAAAAT

8,840 8,850 8,860 8,870 8,880 8,890 8,900 8,910

TATAAAACAAAAATATAATAATTCAAAATTAATTACATTAAATCTTAATAAATAAAAATCTAAAACTCCTCAAAATTTAT

TATAAAACAAAAATATAATAATTCAAAATTAATTACATTAAATCTTAATAAATAAAAATCTAAAACTCCTCAAAATTTAT

TATAAAACAAAAATATAATAATTCAAAATTAATTACATTAAATCTTAATAAATAAAAATCTAAAACTCCTCAAAATTTAT

TATAAAACAAAAATATAATAATTCAAAATTAATTACATTAAATCTTAATAAATAAAAATCTAAAACTCCTCAAAATTTAT

TATAAAACAAAAATATAATAATTCAAAATTAATTACATTAAATCTTAATAAATAAAAATCTAAAACTCCTCAAAATTTAT

8,920 8,930 8,940 8,950 8,960 8,970 8,980 8,990

ATAAATAAAAAATTCCTCCTAATATAGGTAAAGATACAAATAATGTATAAAATAATAAATAAATTCCAGCTTGTAACCGC

ATAAATAAAAAATTCCTCCTAATATAGGTAAAGATACAAATAATGTATAAAATAATAAATAAATTCCAGCTTGTAACCGC

ATAAATAAAAAATTCCTCCTAATATAGGTAAAGATACAAATAATGTATAAAATAATAAATAAATTCCAGCTTGTAACCGC

ATAAATAAAAAATTCCTCCTAATATAGGTAAAGATACAAATAATGTATAAAATAATAAATAAATTCCAGCTTGTAACCGC

ATAAATAAAAAATTCCTCCTAATATAGGTAAAGATACAAATAATGTATAAAATAATAAATAAATTCCAGCTTGTAACCGC

9,000 9,010 9,020 9,030 9,040 9,050 9,060 9,070

TCAGGTTGATAACCTCATCCTAAAATTAAAAATAAAGTTGGAATTAATCTTCTCTCAAAAAATAAATAAAATTTAAAGAT

TCAGGTTGATAACCTCATCCTAAAATTAAAAATAAAGTTGGAATTAATCTTCTCTCAAAAAATAAATAAAATTTAAAGAT

TCAGGTTGATAACCTCATCCTAAAATTAAAAATAAAGTTGGAATTAATCTTCTCTCAAAAAATAAATAAAATTTAAAGAT

TCAGGTTGATAACCTCATCCTAAAATTAAAAATAAAGTTGGAATTAATCTTCTCTCAAAAAATAAATAAAATTTAAAGAT

TCAGGTTGATAACCTCATCCTAAAATTAAAAATAAAGTTGGAATTAATCTTCTCTCAAAAAATAAATAAAATTTAAAGAT

9,080 9,090 9,100 9,110 9,120 9,130 9,140 9,150 9,160

ATTTATTCTAAAAAAAGTCAATAATAACAATAATAATAACAACAACATATTTAAAATATAAAGATAAATAAAATTATTTAAATTAATAAAT

ATTTATTCTAAAAAAAGTCAATAATAACAATAATAATAACAACAACATATTTAAAATATAAAGATAAATAAAATTATTTAAATTAATAAAT

ATTTATTCTAAAAAAAGTCAATAATAACAATAATAATAACAACAACATATTTAAAATATAAAGATAAATAAAATTATTTAAATTAATAAAT

ATTTATTCTAAAAAAAGTCAATAATAACAATAATAATAACAACAACATATTTAAAATATAAAGATAAATAAAATTATTTAAATTAATAAAT

ATTTATTCTAAAAAAAGTCAATAATAACAATAATAATAACAACAACATATTTAAAATATAAAGATAAATAAAATTATTTAAATTAATAAAT

9,170 9,180 9,190 9,200 9,210 9,220 9,230

TTTTTCTCTTGCCAAAATTATTAAACTAATAATTCAAACTGATAACAATACAAATCCATAAGAAATTAAATCTCC

TTTTTCTCTTGCCAAAATTATTAAACTAATAATTCAAACTGATAACAATACAAATCCATAAGAAATTAAATCTCC

TTTTTCTCTTGCCAAAATTATTAAACTAATAATTCAAACTGATAACAATACAAATCCATAAGAAATTAAATCTCC

TTTTTCTCTTGCCAAAATTATTAAACTAATAATTCAAACTGATAACAATACAAATCCATAAGAAATTAAATCTCC

TTTTTCTCTTGCCAAAATTATTAAACTAATAATTCAAACTGATAACAATACAAATCCATAAGAAATTAAATCTCC

9,240 9,250 9,260 9,270 9,280 9,290 9,300 9,310 9,320

TCCAAATCCATAAGAAACTCTTATATAATAATTACTAAATATATGTAAAATAATTACTAAAAATCTTAATATAAATATTAAATTT

TCCAAATCCATAAGAAACTCTTATATAATAATTACTAAATATATGTAAAATAATTACTAAAAATCTTAATATAAATATTAAATTT

TCCAAATCCATAAGAAACTCTTATATAATAATTACTAAATATATGTAAAATAATTACTAAAAATCTTAATATAAATATTAAATTT

TCCAAATCCATAAGAAACTCTTATATAATAATTACTAAATATATGTAAAATAATTACTAAAAATCTTAATATAAATATTAAATTT

TCCAAATCCATAAGAAACTCTTATATAATAATTACTAAATATATGTAAAATAATTACTAAAAATCTTAATATAAATATTAAATTT

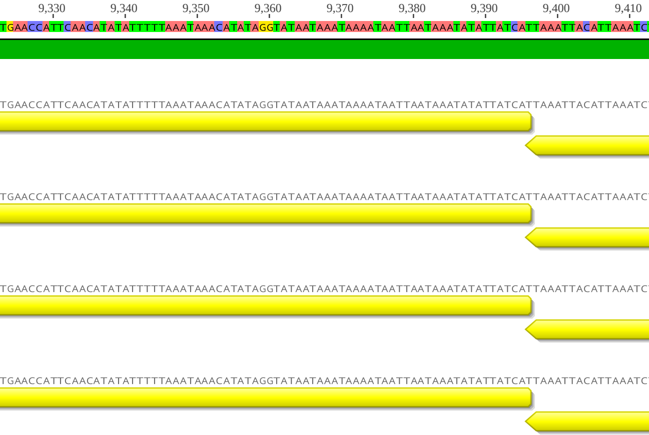
9,330
9,340
9,350
9,360
9,370
9,380
9,390
9,400
9,410
TGAACCATTCAACATATATTTTTAAATAAACATATAGGTATAATAAATAAAATAATTAATAAATATATTATCATTAAATTACATTAAATC
TGAACCATTCAACATATATTTTTAAATAAACATATAGGTATAATAAATAAAATAATTAATAAATATATTATCATTAAATTACATTAAATC
TGAACCATTCAACATATATTTTTAAATAAACATATAGGTATAATAAATAAAATAATTAATAAATATATTATCATTAAATTACATTAAATC
TGAACCATTCAACATATATTTTTAAATAAACATATAGGTATAATAAATAAAATAATTAATAAATATATTATCATTAAATTACATTAAATC
TGAACCATTCAACATATATTTTTAAATAAACATATAGGTATAATAAATAAAATAATTAATAAATATATTATCATTAAATTACATTAAATC

9,420 9,430 9,440 9,450 9,460 9,470 9,480 9,490

TTTGAAAATAATCATTACCATGAGTTCGAATTATTGAAACCAAAATTGAAATACCTAAAGAACTCTCACATACTATAAAAGT

TTTGAAAATAATCATTACCATGAGTTCGAATTATTGAAACCAAAATTGAAATACCTAAAGAACTCTCACATACTATAAAAGT

TTTGAAAATAATCATTACCATGAGTTCGAATTATTGAAACCAAAATTGAAATACCTAAAGAACTCTCACATACTATAAAAGT

TTTGAAAATAATCATTACCATGAGTTCGAATTATTGAAACCAAAATTGAAATACCTAAAGAACTCTCACATACTATAAAAGT

TTTGAAAATAATCATTACCATGAGTTCGAATTATTGAAACCAAAATTGAAATACCTAAAGAACTCTCACATACTATAAAAGT

9,500 9,510 9,520 9,530 9,540 9,550 9,560 9,570 9,580

TCAAAAATAATATACTAAAATATAATTCAAACTCCATATAACTTAAATATAAAAATAAATAAATAAATAATCTTAAAACAATATATTCT

TCAAAAATAATATACTAAAATATAATTCAAACTCCATATAACTTAAATATAAAAATAAATAAATAAATAATCTTAAAACAATATATTCT

ND4L CDS

TCAAAAATAATATACTAAAATATAATTCAAACTCCATATAACTTAAATATAAAAATAAATAAATAAATAATCTTAAAACAATATATTCT

ND4L CDS

TCAAAAATAATATACTAAAATATAATTCAAACTCCATATAACTTAAATATAAAAATAAATAAATAAATAATCTTAAAACAATATATTCT

ND4L CDS

TCAAAAATAATATACTAAAATATAATTCAAACTCCATATAACTTAAATATAAAAATAAATAAATAAATAATCTTAAAACAATATATTCT

ND4L CDS

9,590 9,600 9,610 9,620 9,630 9,640 9,650 9,660

TAATCTTAATAATATTGATAATAAATGCTTACAATTCAAAATAAATACTAATACTCCTATAATAAATAAAATAATAGAAAAATTAAT

TAATCTTAATAATATTGATAATAAATGCTTACAATTCAAAATAAATACTAATACTCCTATAATAAATAAAATAATAGAAAAATTAAT

TAATCTTAATAATATTGATAATAAATGCTTACAATTCAAAATAAATACTAATACTCCTATAATAAATAAAATAATAGAAAAATTAAT

TAATCTTAATAATATTGATAATAAATGCTTACAATTCAAAATAAATACTAATACTCCTATAATAAATAAAATAATAGAAAAATTAAT

TAATCTTAATAATATTGATAATAAATGCTTACAATTCAAAATAAATACTAATACTCCTATAATAAATAAAATAATAGAAAAATTAAT

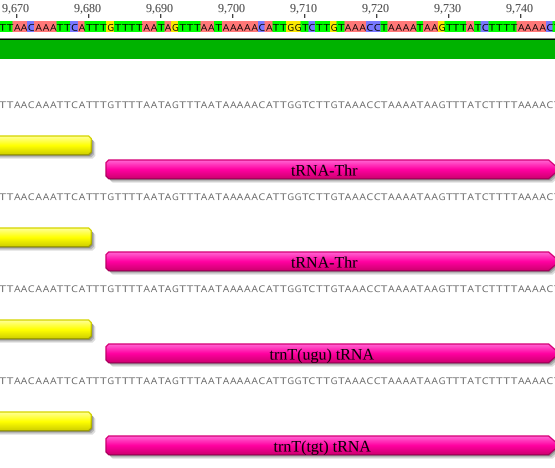
9,670
9,680
9,690
9,700
9,710
9,720
9,730
9,740
TTAACAAATTCATTTGTTTTAATAGTTTAATAAAAACATTGGTCTTGTAAACCTAAAATAAGTTTATCTTTTAAAAC
TTAACAAATTCATTTGTTTTAATAGTTTAATAAAAACATTGGTCTTGTAAACCTAAAATAAGTTTATCTTTTAAAAC
tRNA-Thr
TTAACAAATTCATTTGTTTTAATAGTTTAATAAAAACATTGGTCTTGTAAACCTAAAATAAGTTTATCTTTTAAAAC
tRNA-Thr
TTAACAAATTCATTTGTTTTAATAGTTTAATAAAAACATTGGTCTTGTAAACCTAAAATAAGTTTATCTTTTAAAAC
trnT(ugu) tRNA
TTAACAAATTCATTTGTTTTAATAGTTTAATAAAAACATTGGTCTTGTAAACCTAAAATAAGTTTATCTTTTAAAAC
trnT(tgt) tRNA

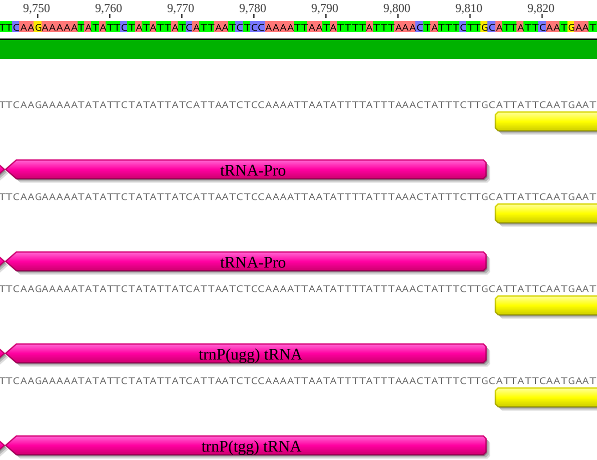
9,750
9,760
9,770
9,780
9,790
9,800
9,810
9,820
TTCAAGAAAAATATATTCTATATTATCATTAATCTCCAAAATTAATATTTTATTTAAACTATTTCTTGCATTATTCAATGAAT
TTCAAGAAAAATATATTCTATATTATCATTAATCTCCAAAATTAATATTTTATTTAAACTATTTCTTGCATTATTCAATGAAT
tRNA-Pro
TTCAAGAAAAATATATTCTATATTATCATTAATCTCCAAAATTAATATTTTATTTAAACTATTTCTTGCATTATTCAATGAAT
tRNA-Pro
TTCAAGAAAAATATATTCTATATTATCATTAATCTCCAAAATTAATATTTTATTTAAACTATTTCTTGCATTATTCAATGAAT
trnP(ugg) tRNA
TTCAAGAAAAATATATTCTATATTATCATTAATCTCCAAAATTAATATTTTATTTAAACTATTTCTTGCATTATTCAATGAAT
trnP(tgg) tRNA

9,830 9,840 9,850 9,860 9,870 9,880 9,890 9,900 9,910

TCTACTTATATTTAGATTAACACTTAATTTAATTTTTATATTTATAAATCACCCTTTAGCCATAGGCTTTGTTTTATTAATTCAAACTAC

TCTACTTATATTTAAATTAACACTTAATTTAATTTTTATATTTATAAATCACCCTTTAGCCATAGGCTTTGTTTTATTAATTCAAACTAC

TCTACTTATATTTAGATTAACACTTAATTTAATTTTTATATTTATAAATCACCCTTTAGCCATAGGCTTTGTTTTATTAATTCAAACTAC

TCTACTTATATTTAGATTAACACTTAATTTAATTTTTATATTTATAAATCACCCTTTAGCCATAGGCTTTGTTTTATTAATTCAAACTAC

TCTACTTATATTTAGATTAACACTTAATTTAATTTTTATATTTATAAATCACCCTTTAGCCATAGGCTTTGTTTTATTAATTCAAACTAC

9,920 9,930 9,940 9,950 9,960 9,970 9,980 9,990 10,000

TTTAATTTCAATAATTACAGGATTAATAACATACTCATTTTGATTTTCTTATATTTTATTTATTGTATTCTTAGGAGGAATATTAG

TTTAATTTCAATAATTACAGGATTAATAACATACTCATTTTGATTTTCTTATATTTTATTTATTGTATTCTTAGGAGGAATATTAG

TTTAATTTCAATAATTACAGGATTAATAACATACTCATTTTGATTTTCTTATATTTTATTTATTGTATTCTTAGGAGGAATATTAG

TTTAATTTCAATAATTACAGGATTAATAACATACTCATTTTGATTTTCTTATATTTTATTTATTGTATTCTTAGGAGGAATATTAG

TTTAATTTCAATAATTACAGGATTAATAACATACTCATTTTGATTTTCTTATATTTTATTTATTGTATTCTTAGGAGGAATATTAG

10,010 10,020 10,030 10,040 10,050 10,060 10,070 10,080

TATTATTTATTTATATTTCATCACTAGCATCTAATGAAATATTTTCTCTATCAATAAAATTATTTATAACTTCAATTAT

TATTATTTATTTATATTTCATCACTAGCATCTAATGAAATATTTTCTCTATCAATAAAATTATTTATAACTTCAATTAT

ND6 CDS

TATTATTTATTTATATTTCATCACTAGCATCTAATGAAATATTTTCTCTATCAATAAAATTATTTATAACTTCAATTAT

ND6 CDS

TATTATTTATTTATATTTCATCACTAGCATCTAATGAAATATTTTCTCTATCAATAAAATTATTTATAACTTCAATTAT

ND6 CDS

TATTATTTATTTATATTTCATCACTAGCATCTAATGAAATATTTTCTCTATCAATAAAATTATTTATAACTTCAATTAT

ND6 CDS

10,090 10,100 10,110 10,120 10,130 10,140 10,150 10,160 10,170

TTTCATAATCATAATAACAATTATTATTTTATTTATTGATAAAAATATAAATATAATATCTATAAATTTTGAAACTAAAAATTTTAATAA

TTTCATAATCATAATAACAATTATTATTTTATTTATTGATAAAAATATAAATATAATATCTATAAATTTTGAAACTAAAAATTTTAATAA

TTTCATAATCATAATAACAATTATTATTTTATTTATTGATAAAAATATAAATATAATATCTATAAATTTTGAAACTAAAAATTTTAATAA

TTTCATAATCATAATAACAATTATTATTTTATTTATTGATAAAAATATAAATATAATATCTATAAATTTTGAAACTAAAAATTTTAATAA

TTTCATAATCATAATAACAATTATTATTTTATTTATTGATAAAAATATAAATATAATATCTATAAATTTTGAAACTAAAAATTTTAATAA

10,180 10,190 10,200 10,210 10,220 10,230 10,240 10,250

TAAAATTTATATTCAAGAAAATTCATTATTAATTAAAATATATAACTATCCATATAACATTATTATTATTATCCTAATAAATTAT

TAAAATTTATATTCAAGAAAATTCATTATTAATTAAAATATATAACTATCCATATAACATTATTATTATTATCCTAATAAATTAT

TAAAATTTATATTCAAGAAAATTCATTATTAATTAAAATATATAACTATCCATATAACATTATTATTATTATCCTAATAAATTAT

TAAAATTTATATTCAAGAAAATTCATTATTAATTAAAATATATAACTATCCATATAACATTATTATTATTATCCTAATAAATTAT

TAAAATTTATATTCAAGAAAATTCATTATTAATTAAAATATATAACTATCCATATAACATTATTATTATTATCCTAATAAATTAT

10,260 10,270 10,280 10,290 10,300 10,310 10,320 10,330

TTATTAATTACACTAATTGCTATTGTAAAAATTACTAAACTTTCAAAAGGACCTTTACGACCAATATTTAATTAAT

TTTATTAATTACACTAATTGCTATTGTAAAAATTACTAAACTTTCAAAAGGACCTTTACGACCAATATTTAATTAAT

TTTATTAATTACACTAATTGCTATTGTAAAAATTACTAAACTTTCAAAAGGACCTTTACGACCAATATTTAATTAAT

TTTATTAATTACACTAATTGCTATTGTAAAAATTACTAAACTTTCAAAAGGACCTTTACGACCAATATTTAATTAAT

TTTATTAATTACACTAATTGCTATTGTAAAAATTACTAAACTTTCAAAAGGACCTTTACGACCAATATTTAATTAAT

10,340 10,350 10,360 10,370 10,380 10,390 10,400 10,410 10,420

GAATAAACCTATACGTCTTAATAATCCTATTTTTAAAATTGCAAATAATTCCCTAATTGACTTACCAACACCTATTAATATCTCATCAT

TGAATAAACCTATACGTCTTAATAATCCTATTTTTAAAATTGCAAATAATTCCCTAATTGACTTACCAACACCTATTAATATCTCATCAT

TGAATAAACCTATACGTCTTAATAATCCTATTTTTAAAATTGCAAATAATTCCCTAATTGACTTACCAACACCTATTAATATCTCATCAT

TGAATAAACCTATACGTCTTAATAATCCTATTTTTAAAATTGCAAATAATTCCCTAATTGACTTACCAACACCTATTAATATCTCATCAT

TGAATAAACCTATACGTCTTAATAATCCTATTTTTAAAATTGCAAATAATTCCCTAATTGACTTACCAACACCTATTAATATCTCATCAT

10,430 10,440 10,450 10,460 10,470 10,480 10,490 10,500 10,510

GATGAAACTTTGGCTCTTTATTAGGCTTATGTCTAATTATCCAAATTATATCAGGATTATTTTTAGCAATACATTATACATCTGATATTT

GATGAAACTTTGGCTCTTTATTAGGCTTATGTCTAATTATCCAAATTATATCAGGATTATTTTTAGCAATACATTATACATCTGATATTT

GATGAAACTTTGGCTCTTTATTAGGCTTATGTCTAATTATCCAAATTATATCAGGATTATTTTTAGCAATACATTATACATCTGATATTT

GATGAAACTTTGGCTCTTTATTAGGCTTATGTCTAATTATCCAAATTATATCAGGATTATTTTTAGCAATACATTATACATCTGATATTT

GATGAAACTTTGGCTCTTTATTAGGCTTATGTCTAATTATCCAAATTATATCAGGATTATTTTTAGCAATACATTATACATCTGATATTT

10,520 10,530 10,540 10,550 10,560 10,570 10,580 10,590

CTTTAGCATTTAATAGAATTATTCATATTTGTCGAAATGTAAATTATGGTTGAATATTACGAACCATTCATGCTAATGGAGCA

CTTTAGCATTTAATAGAATTATTCATATTTGTCGAAATGTAAATTATGGTTGAATATTACGAACCATTCATGCTAATGGAGCAT

CTTTAGCATTTAATAGAATTATTCATATTTGTCGAAATGTAAATTATGGTTGAATATTACGAACCATTCATGCTAATGGAGCAT

CTTTAGCATTTAATAGAATTATTCATATTTGTCGAAATGTAAATTATGGTTGAATATTACGAACCATTCATGCTAATGGAGCAT

CTTTAGCATTTAATAGAATTATTCATATTTGTCGAAATGTAAATTATGGTTGAATATTACGAACCATTCATGCTAATGGAGCAT

10,600 10,610 10,620 10,630 10,640 10,650 10,660 10,670 10,680

CTTTATTTTTTTTATGTATTTATTTTCATATTGGTCGAAGAATATATTATGGATCCTATTTATATACTCATACCTGAATAATTGGAA

CTTTATTTTTTTTATGTATTTATTTTCATATTGGTCGAAGAATATATTATGGATCCTATTTATATACTCATACCTGAATAATTGGAA

CTTTATTTTTTTTATGTATTTATTTTCATATTGGTCGAAGAATATATTATGGATCCTATTTATATACTCATACCTGAATAATTGGAA

CTTTATTTTTTTTATGTATTTATTTTCATATTGGTCGAAGAATATATTATGGATCCTATTTATATACTCATACCTGAATAATTGGAA

CTTTATTTTTTTTATGTATTTATTTTCATATTGGTCGAAGAATATATTATGGATCCTATTTATATACTCATACCTGAATAATTGGAA

10,690 10,700 10,710 10,720 10,730 10,740 10,750 10,760 10,770

TTATAATTCTATTTATATTAATAATAACAGCTTTTATAGGTTATGTATTACCTTGAGGACAAATATCATTCTGAGGAGCAACAGTAATT

TTATAATTCTATTTATATTAATAATAACAGCTTTTATAGGTTATGTATTACCTTGAGGACAAATATCATTCTGAGGAGCAACAGTAATT

TTATAATTCTATTTATATTAATAATAACAGCTTTTATAGGTTATGTATTACCTTGAGGACAAATATCATTCTGAGGAGCAACAGTAATT

TTATAATTCTATTTATATTAATAATAACAGCTTTTATAGGTTATGTATTACCTTGAGGACAAATATCATTCTGAGGAGCAACAGTAATT

TTATAATTCTATTTATATTAATAATAACAGCTTTTATAGGTTATGTATTACCTTGAGGACAAATATCATTCTGAGGAGCAACAGTAATT

10,780 10,790 10,800 10,810 10,820 10,830 10,840 10,850 10,860

ACTAATTTACTATCAGCAATTCCTTACTTAGGTAATAATTTAGTTCAATGAATTTGAGGAGGATTCTCTATTGATAATGCAACATTAACT

ACTAATTTACTATCAGCAATTCCTTACTTAGGTAATAATTTAGTTCAATGAATTTGAGGAGGATTCTCTATTGATAATGCAACATTAACT

ACTAATTTACTATCAGCAATTCCTTACTTAGGTAATAATTTAGTTCAATGAATTTGAGGAGGATTCTCTATTGATAATGCAACATTAACT

ACTAATTTACTATCAGCAATTCCTTACTTAGGTAATAATTTAGTTCAATGAATTTGAGGAGGATTCTCTATTGATAATGCAACATTAACT

ACTAATTTACTATCAGCAATTCCTTACTTAGGTAATAATTTAGTTCAATGAATTTGAGGAGGATTCTCTATTGATAATGCAACATTAACT

10,870 10,880 10,890 10,900 10,910 10,920 10,930 10,940

CGATTCTTTACATTCCACTTTATTTTACCTTTTATTATTATAGCTATAACAATAATTCATCTTATATTTTTACATGAAACAGGA

CGATTCTTTACATTCCACTTTATTTTACCTTTTATTATTATAGCTATAACAATAATTCATCTTATATTTTTACATGAAACAGGA

CYTB CDS

CGATTCTTTACATTCCACTTTATTTTACCTTTTATTATTATAGCTATAACAATAATTCATCTTATATTTTTACATGAAACAGGA

CYTB CDS

CGATTCTTTACATTCCACTTTATTTTACCTTTTATTATTATAGCTATAACAATAATTCATCTTATATTTTTACATGAAACAGGA

CYTB CDS

CGATTCTTTACATTCCACTTTATTTTACCTTTTATTATTATAGCTATAACAATAATTCATCTTATATTTTTACATGAAACAGGA

CYTB CDS

10,950 10,960 10,970 10,980 10,990 11,000 11,010 11,020 11,030

TCAAATAATCCTATTGGATTAAATTCTAATATTGATAAAATTCCATTTCATCCATATTTTTCCTATAAAGATATAATAGGATTTATT

TCAAATAATCCTATTGGATTAAATTCTAATATTGATAAAATTCCATTTCATCCATATTTTTCCTATAAAGATATAATAGGATTTATT

TCAAATAATCCTATTGGATTAAATTCTAATATTGATAAAATTCCATTTCATCCATATTTTTCCTATAAAGATATAATAGGATTTATT

TCAAATAATCCTATTGGATTAAATTCTAATATTGATAAAATTCCATTTCATCCATATTTTTCCTATAAAGATATAATAGGATTTATT

TCAAATAATCCTATTGGATTAAATTCTAATATTGATAAAATTCCATTTCATCCATATTTTTCCTATAAAGATATAATAGGATTTATT

11,040 11,050 11,060 11,070 11,080 11,090 11,100 11,110

ATTTTAATAATAATCTTATTATTATTAACCTTGACTAATCCTTATATATTAAGTGATCCTGATAATTTTACTCCTGCAAACCC

TATTTTAATAATAATATTATTATTATTAACCTTGACTAATCCTTATATATTAAGTGATCCTGATAATTTTACTCCTGCAAACCC

TATTTTAATAATAATCTTATTATTATTAACCTTGACTAATCCTTATATATTAAGTGATCCTGATAATTTTACTCCTGCAAACCC

TATTTTAATAATAATCTTATTATTATTAACCTTGACTAATCCTTATATATTAAGTGATCCTGATAATTTTACTCCTGCAAACCC

TATTTTAATAATAATCTTATTATTATTAACCTTGACTAATCCTTATATATTAAGTGATCCTGATAATTTTACTCCTGCAAACCC

11,120 11,130 11,140 11,150 11,160 11,170 11,180 11,190

TCTAATTACTCCTCCACATATTCAACCAGAATGATATTTTTTATTTGCATATGCTATTCTACGATCTATTCCTAACAAAC

TCTAATTACTCCTCCACATATTCAACCAGAATGATATTTTTTATTTGCATATGCTATTCTACGATCTATTCCTAACAAAC

TCTAATTACTCCTCCACATATTCAACCAGAATGATATTTTTTATTTGCATATGCTATTCTACGATCTATTCCTAACAAAC

TCTAATTACTCCTCCACATATTCAACCAGAATGATATTTTTTATTTGCATATGCTATTCTACGATCTATTCCTAACAAAC

TCTAATTACTCCTCCACATATTCAACCAGAATGATATTTTTTATTTGCATATGCTATTCTACGATCTATTCCTAACAAAC

11,200 11,210 11,220 11,230 11,240 11,250 11,260 11,270 11,280

TAGGAGGAGTAATTGCCCTAATTTCATCAATTACTATTCTAATAATTTTACCATTTTTTCATTTAAGTAAATTTCGAGGAATTCAATTTT

TAGGAGGAGTAATTGCCCTAATTTCATCAATTACTATTCTAATAATTTTACCATTTTTTCATTTAAGTAAATTTCGAGGAATTCAATTTT

TAGGAGGAGTAATTGCCCTAATTTCATCAATTACTATTCTAATAATTTTACCATTTTTTCATTTAAGTAAATTTCGAGGAATTCAATTTT

TAGGAGGAGTAATTGCCCTAATTTCATCAATTACTATTCTAATAATTTTACCATTTTTTCATTTAAGTAAATTTCGAGGAATTCAATTTT

TAGGAGGAGTAATTGCCCTAATTTCATCAATTACTATTCTAATAATTTTACCATTTTTTCATTTAAGTAAATTTCGAGGAATTCAATTTT

11,290 11,300 11,310 11,320 11,330 11,340 11,350 11,360

ATCCAATAAATAAAATAATATTCTGATCAATAACTATAATTATAATAATACTAACATGAATTGGAGCTAAACCTGTT

TATCCAATAAATAAAATAATATTCTGATCAATAACTATAATTATAATAATACTAACATGAATTGGAGCTAAACCTGTT

TATCCAATAAATAAAATAATATTCTGATCAATAACTATAATTATAATAATACTAACATGAATTGGAGCTAAACCTGTT

TATCCAATAAATAAAATAATATTCTGATCAATAACTATAATTATAATAATACTAACATGAATTGGAGCTAAACCTGTT

TATCCAATAAATAAAATAATATTCTGATCAATAACTATAATTATAATAATACTAACATGAATTGGAGCTAAACCTGTT

11,370 11,380 11,390 11,400 11,410 11,420 11,430 11,440

GAAGATCCATACATTATTATTGGACAATTACTAACAATCTTATATTTTTCATACTATTTATTATTTCCTATTACAACTAAAT

GAAGATCCATACATTATTATTGGACAATTACTAACAATCTTATATTTTTCATACTATTTATTATTTCCTATTACAACTAAAT

GAAGATCCATACATTATTATTGGACAATTACTAACAATCTTATATTTTTCATACTATTTATTATTTCCTATTACAACTAAAT

GAAGATCCATACATTATTATTGGACAATTACTAACAATCTTATATTTTTCATACTATTTATTATTTCCTATTACAACTAAAT

GAAGATCCATACATTATTATTGGACAATTACTAACAATCTTATATTTTTCATACTATTTATTATTTCCTATTACAACTAAAT

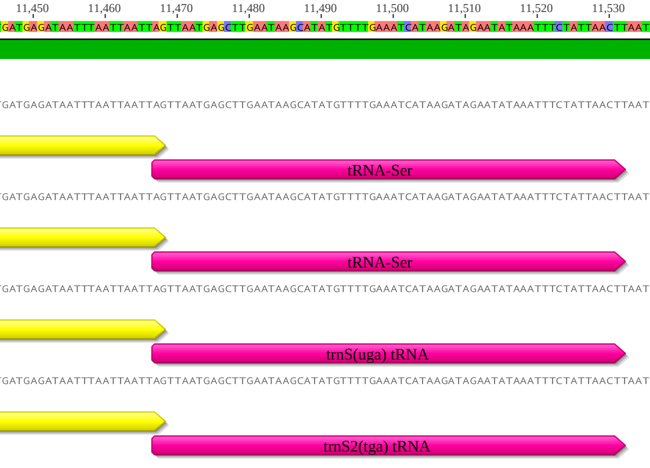

11,450 11,460 11,470 11,480 11,490 11,500 11,510 11,520 11,530
GATGAGATAATTTAATTAATTAGTTAATGAGCTTGAATAAGCATATGTTTTGAAATCATAAGATAGAATATAAATTTCTATTAACTTAAT
tRNA-Ser
tRNA-Ser
trnS(uga) tRNA
trnS2(tga) tRNA

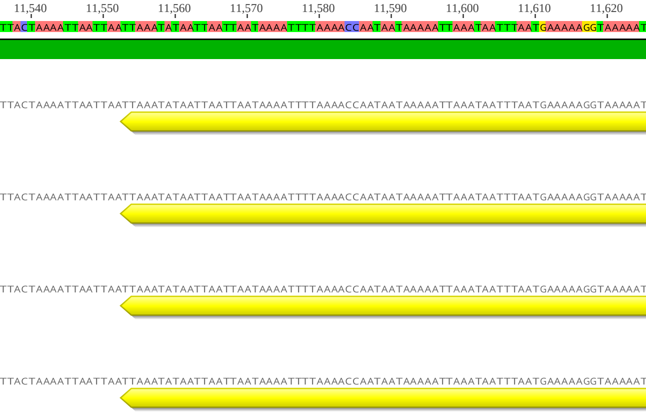
11,540 11,550 11,560 11,570 11,580 11,590 11,600 11,610 11,620
TTACTAAAATTAATTAATTAAATATAATTAATTAATAAAATTTTAAAACCAATAATAAAAATTAAATAATTTAATGAAAAAGGTAAAAAT
TTACTAAAATTAATTAATTAAATATAATTAATTAATAAAATTTTAAAACCAATAATAAAAATTAAATAATTTAATGAAAAAGGTAAAAAT
TTACTAAAATTAATTAATTAAATATAATTAATTAATAAAATTTTAAAACCAATAATAAAAATTAAATAATTTAATGAAAAAGGTAAAAAT
TTACTAAAATTAATTAATTAAATATAATTAATTAATAAAATTTTAAAACCAATAATAAAAATTAAATAATTTAATGAAAAAGGTAAAAAT
TTACTAAAATTAATTAATTAAATATAATTAATTAATAAAATTTTAAAACCAATAATAAAAATTAAATAATTTAATGAAAAAGGTAAAAAT

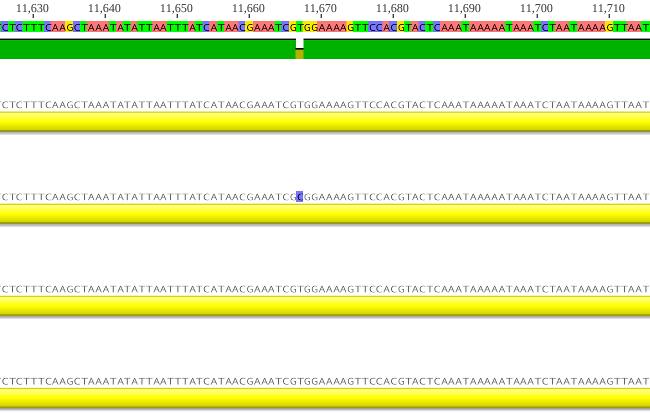
11,630
11,640
11,650
11,660
11,670
11,680
11,690
11,700
11,710
CTCTTTCAAGCTAAATATATTAATTTATCATAACGAAATCGTGGAAAAGTTCCACGTACTCAAATAAAAATAAATCTAATAAAAGTTAAT
CTCTTTCAAGCTAAATATATTAATTTATCATAACGAAATCGTGGAAAAGTTCCACGTACTCAAATAAAAATAAATCTAATAAAAGTTAAT
CTCTTTCAAGCTAAATATATTAATTTATCATAACGAAATCGCGGAAAAGTTCCACGTACTCAAATAAAAATAAATCTAATAAAAGTTAAT
CTCTTTCAAGCTAAATATATTAATTTATCATAACGAAATCGTGGAAAAGTTCCACGTACTCAAATAAAAATAAATCTAATAAAAGTTAAT
CTCTTTCAAGCTAAATATATTAATTTATCATAACGAAATCGTGGAAAAGTTCCACGTACTCAAATAAAAATAAATCTAATAAAAGTTAAT

11,720 11,730 11,740 11,750 11,760 11,770 11,780 11,790

TTAATAAAAAAATATAAAGATAAAATATTACCTCCCAAAAATATTAATCTAAAAATTATTCTTATAAATAAAATTCTT

TTAATAAAAAAATATAAAGATAAAATATTACCTCCCAAAAATATTAATCTAAAAATTATTCTTATAAATAAAATTCTT

TTAATAAAAAAATATAAAGATAAAATATTACCTCCCAAAAATATTAATCTAAAAATTATTCTTATAAATAAAATTCTT

TTAATAAAAAAATATAAAGATAAAATATTACCTCCCAAAAATATTAATCTAAAAATTATTCTTATAAATAAAATTCTT

TTAATAAAAAAATATAAAGATAAAATATTACCTCCCAAAAATATTAATCTAAAAATTATTCTTATAAATAAAATTCTT

11,800 11,810 11,820 11,830 11,840 11,850 11,860 11,870

GAATATTCAGCTAAAAAAATTAATGCAAATCCTCCTCTTCTATATTCAACATTAAATCCTGAAACTAACTCTGACTCTCCTT

TGAATATTCAGCTAAAAAAATTAATGCAAATCCTCCTCTTCTATATTCAACATTAAATCCTGAAACTAACTCTGACTCTCCTT

TGAATATTCAGCTAAAAAAATTAATGCAAATCCTCCTCTTCTATATTCAACATTAAATCCTGAAACTAACTCTGACTCTCCTT

TGAATATTCAGCTAAAAAAATTAATGCAAATCCTCCTCTTCTATATTCAACATTAAATCCTGAAACTAACTCTGACTCTCCTT

TGAATATTCAGCTAAAAAAATTAATGCAAATCCTCCTCTTCTATATTCAACATTAAATCCTGAAACTAACTCTGACTCTCCTT

11,880 11,890 11,900 11,910 11,920 11,930 11,940 11,950 11,960

CAGCAAAATCAAAAGGAGTACGATTAGTTTCAGCTAACATAATTCTAATTCAAACTAAACCTAAAGGAAAAGAAAAAAAAATAAATCAAA

CAGCAAAATCAAAAGGAGTACGATTAGTTTCAGCTAACATAATTCTAATTCAAACTAAACCTAAAGGAAAAGAAAAAAAAATAAATCAAA

CAGCAAAATCAAAAGGAGTACGATTAGTTTCAGCTAACATAATTCTAATTCAAACTAAACCTAAAGGAAAAGAAAAAAAAATAAATCAAA

CAGCAAAATCAAAAGGAGTACGATTAGTTTCAGCTAACATAATTCTAATTCAAACTAAACCTAAAGGAAAAGAAAAAAAAATAAATCAAA

CAGCAAAATCAAAAGGAGTACGATTAGTTTCAGCTAACATAATTCTAATTCAAACTAAACCTAAAGGAAAAGAAAAAAAAATAAATCAAA

11,970 11,980 11,990 12,000 12,010 12,020 12,030 12,040

TATATATTTGATATTTAAAAAATATAAGTATATTAAACCTTTCAATTAAAAAAATTAATCTTATCAAAATTAAACCCAATC

TATATATTTGATATTTAAAAAATATAAGTATATTAAACCTTTCAATTAAAAAAATTAATCTTATCAAAATTAAACCCAATC

ND1 CDS

TATATATTTGATATTTAAAAAATATAAGTATATTAAACCTTTCAATTAAAAAAATTAATCTTATCAAAATTAAACCCAATC

ND1 CDS

TATATATTTGATATTTAAAAAATATAAGTATATTAAACCTTTCAATTAAAAAAATTAATCTTATCAAAATTAAACCCAATC

ND1 CDS

TATATATTTGATATTTAAAAAATATAAGTATATTAAACCTTTCAATTAAAAAAATTAATCTTATCAAAATTAAACCCAATC

ND1 CDS

12,050 12,060 12,070 12,080 12,090 12,100 12,110 12,120 12,130

TAACTTCATAAGAAATTGTTTGAGCAACAGCTCGTAATCCTCCTAATAAAGCATAAATTGAATTAGATGATCAACCAGCAATTATTACTC

TAACTTCATAAGAAATTGTTTGAGCAACAGCTCGTAATCCTCCTAATAAAGCATAAATTGAATTAGATGATCAACCAGCAATTATTACTC

TAACTTCATAAGAAATTGTTTGAGCAACAGCTCGTAATCCTCCTAATAAAGCATAAATTGAATTAGATGATCAACCAGCAATTATTACTC

TAACTTCATAAGAAATTGTTTGAGCAACAGCTCGTAATCCTCCTAATAAAGCATAAATTGAATTAGATGATCAACCAGCAATTATTACTC

TAACTTCATAAGAAATTGTTTGAGCAACAGCTCGTAATCCTCCTAATAAAGCATAAATTGAATTAGATGATCAACCAGCAATTATTACTC

12,140 12,150 12,160 12,170 12,180 12,190 12,200 12,210

TATAAACACTTAATCTTAAACAAGATAATAAAAATAATAAACCTAAATTAAAAGAATATAATTTAACAAAATAAGGT

TATAAACACTTAATCTTAAACAAGATAATAAAAATAATAAACCTAAATTAAAAGAATATAATTTAACAAAATAAGGT

TATAAACACTTAATCTTAAACAAGATAATAAAAATAATAAACCTAAATTAAAAGAATATAATTTAACAAAATAAGGT

TATAAACACTTAATCTTAAACAAGATAATAAAAATAATAAACCTAAATTAAAAGAATATAATTTAACAAAATAAGGT

TATAAACACTTAATCTTAAACAAGATAATAAAAATAATAAACCTAAATTAAAAGAATATAATTTAACAAAATAAGGT

12,220 12,230 12,240 12,250 12,260 12,270 12,280 12,290 12,300

ATACATATTCATAATATAATAGATATAAATAAAGAAAAAATAGGTGAAAAATAATATATAATATAATTAGATAATAAAGGATATCTTTGC

ATACATATTCATAATATAATAGATATAAATAAAGAAAAAATAGGTGAAAAATAATATATAATATAATTAGATAATAAAGGATATCTTTGC

ATACATATTCATAATATAATAGATATAAATAAAGAAAAAATAGGTGAAAAATAATATATAATATAATTAGATAATAAAGGATATCTTTGC

ATACATATTCATAATATAATAGATATAAATAAAGAAAAAATAGGTGAAAAATAATATATAATATAATTAGATAATAAAGGATATCTTTGC

ATACATATTCATAATATAATAGATATAAATAAAGAAAAAATAGGTGAAAAATAATATATAATATAATTAGATAATAAAGGATATCTTTGC

12,310 12,320 12,330 12,340 12,350 12,360 12,370 12,380

TCTTTAGTAAATAATTTAATTGCATCACTAAAAGGTTGAAAAATTCCTATAATTCTAACTTTATTAGGACCTTTACGAAC

TCTTTAGTAAATAATTTAATTGCATCACTAAAAGGTTGAAAAATTCCTATAATTCTAACTTTATTAGGACCTTTACGAAC

TCTTTAGTAAATAATTTAATTGCATCACTAAAAGGTTGAAAAATTCCTATAATTCTAACTTTATTAGGACCTTTACGAAC

TCTTTAGTAAATAATTTAATTGCATCACTAAAAGGTTGAAAAATTCCTATAATTCTAACTTTATTAGGACCTTTACGAAC

TCTTTAGTAAATAATTTAATTGCATCACTAAAAGGTTGAAAAATTCCTATAATTCTAACTTTATTAGGACCTTTACGAAC

12,390 12,400 12,410 12,420 12,430 12,440 12,450 12,460

TTGAATATAACCTAAAACTTTTCGTTCAAATAAAGTTAAAAATCCTACACTTACTAAAATAAAAATTAATAATAATATACTTC

TTGAATATAACCTAAAACTTTTCGTTCAAATAAAGTTAAAAATCCTACACTTACTAAAATAAAAATTAATAATAATATACTTC

TTGAATATAACCTAAAACTTTTCGTTCAAATAAAGTTAAAAATCCTACACTTACTAAAATAAAAATTAATAATAATATACTTC

TTGAATATAACCTAAAACTTTTCGTTCAAATAAAGTTAAAAATCCTACACTTACTAAAATAAAAATTAATAATAATATACTTC

TTGAATATAACCTAAAACTTTTCGTTCAAATAAAGTTAAAAATCCTACACTTACTAAAATAAAAATTAATAATAATATACTTC

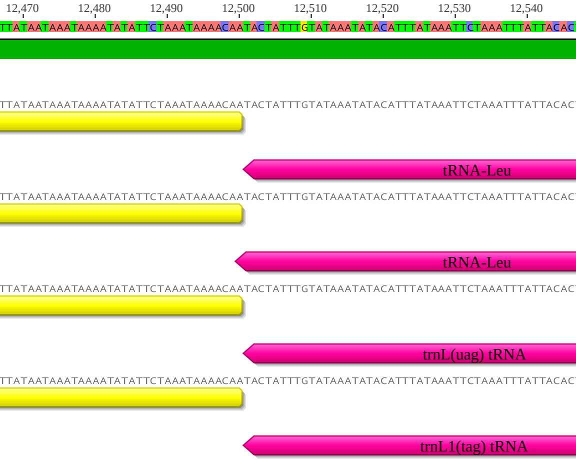

12,470
12,480
12,490
12,500
12,510
12,520
12,530
12,540
TTATAATAAATAAAATATATTCTAAATAAAACAATACTATTTGTATAAATATACATTTATAAATTCTAAATTTATTACAC
TTATAATAAATAAAATATATTCTAAATAAAACAATACTATTTGTATAAATATACATTTATAAATTCTAAATTTATTACAC
tRNA-Leu
TTATAATAAATAAAATATATTCTAAATAAAACAATACTATTTGTATAAATATACATTTATAAATTCTAAATTTATTACAC
tRNA-Leu
TTATAATAAATAAAATATATTCTAAATAAAACAATACTATTTGTATAAATATACATTTATAAATTCTAAATTTATTACAC
trnL(uag) tRNA
TTATAATAAATAAAATATATTCTAAATAAAACAATACTATTTGTATAAATATACATTTATAAATTCTAAATTTATTACAC
trnL1(tag) tRNA

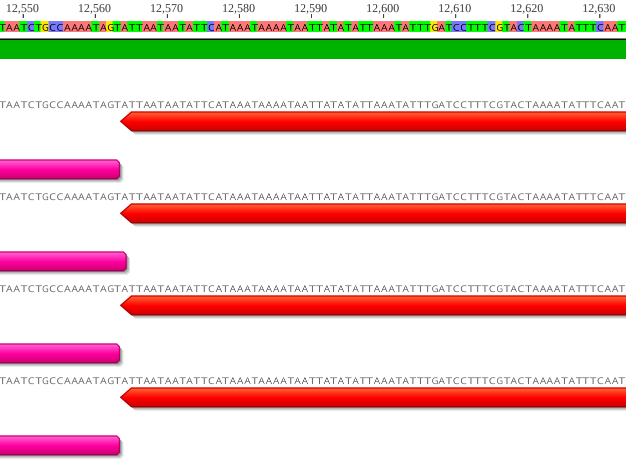

12,550
12,560
12,570
12,580
12,590
12,600
12,610
12,620
12,630
TAATCTGCCAAAATAGTATTAATAATATTCATAAATAAAATAATTATATATTAAATATTTGATCCTTTCGTACTAAAATATTTCAAT
TAATCTGCCAAAATAGTATTAATAATATTCATAAATAAAATAATTATATATTAAATATTTGATCCTTTCGTACTAAAATATTTCAAT
TAATCTGCCAAAATAGTATTAATAATATTCATAAATAAAATAATTATATATTAAATATTTGATCCTTTCGTACTAAAATATTTCAAT
TAATCTGCCAAAATAGTATTAATAATATTCATAAATAAAATAATTATATATTAAATATTTGATCCTTTCGTACTAAAATATTTCAAT
TAATCTGCCAAAATAGTATTAATAATATTCATAAATAAAATAATTATATATTAAATATTTGATCCTTTCGTACTAAAATATTTCAAT

12,640 12,650 12,660 12,670 12,680 12,690 12,700 12,710 12,720

TAACTAAAGATAGAAACCAACCTGGCTTACACCGGTTTGAACTCAGATCATGTAAGAATTTAAAAGTCGAACAGACTTAAATTTTAAGCT

TAACTAAAGATAGAAACCAACCTGGCTTACACCGGTTTGAACTCAGATCATGTAAGAATTTAAAAGTCGAACAGACTTAAATTTTAAGCT

TAACTAAAGATAGAAACCAACCTGGCTTACACCGGTTTGAACTCAGATCATGTAAGAATTTAAAAGTCGAACAGACTTAAATTTTAAGCT

TAACTAAAGATAGAAACCAACCTGGCTTACACCGGTTTGAACTCAGATCATGTAAGAATTTAAAAGTCGAACAGACTTAAATTTTAAGCT

TAACTAAAGATAGAAACCAACCTGGCTTACACCGGTTTGAACTCAGATCATGTAAGAATTTAAAAGTCGAACAGACTTAAATTTTAAGCT

12,730 12,740 12,750 12,760 12,770 12,780 12,790 12,800

TCTTCACCCAAATTTATTTCTTAATCCAACATCGAGGTCGCAATATTTTTTATCAATATGAACTCTCCAAAAAAATTACGCTG

TCTTCACCCAAATTTATTTCTTAATCCAACATCGAGGTCGCAATATTTTTTATCAATATGAACTCTCCAAAAAAATTACGCTG

TCTTCACCCAAATTTATTTCTTAATCCAACATCGAGGTCGCAATATTTTTTATCAATATGAACTCTCCAAAAAAATTACGCTG

TCTTCACCCAAATTTATTTCTTAATCCAACATCGAGGTCGCAATATTTTTTATCAATATGAACTCTCCAAAAAAATTACGCTG

TCTTCACCCAAATTTATTTCTTAATCCAACATCGAGGTCGCAATATTTTTTATCAATATGAACTCTCCAAAAAAATTACGCTG

12,810 12,820 12,830 12,840 12,850 12,860 12,870 12,880 12,890

TTATCCCTAAAGTAACTTATTCTATTAATCAATATTAAAAGATCAAAAAATCATAAATTAATGTATAAATAAATAAAAGTTAATTAAATT

TTATCCCTAAAGTAACTTATTCTATTAATCAATATTAAAAGATCAAAAAATCATAAATTAATGTATAAATAAATAAAAGTTAATTAAATT

TTATCCCTAAAGTAACTTATTCTATTAATCAATATTAAAAGATCAAAAAATCATAAATTAATGTATAAATAAATAAAAGTTAATTAAATT

TTATCCCTAAAGTAACTTATTCTATTAATCAATATTAAAAGATCAAAAAATCATAAATTAATGTATAAATAAATAAAAGTTAATTAAATT

TTATCCCTAAAGTAACTTATTCTATTAATCAATATTAAAAGATCAAAAAATCATAAATTAATGTATAAATAAATAAAAGTTAATTAAATT

12,900 12,910 12,920 12,930 12,940 12,950 12,960 12,970 12,980

TTAATATCACCCCAATAAAATATTTATATAAATAAATAAAATTTAATTTCTAATTTTTATAAACTCAGTAAATATAAAGATTTATAGGGT

TTAATATCACCCCAATAAAATATTTATATAAATAAATAAAATTTAATTTCTAATTTTTATAAACTCAGTAAATATAAAGATTTATAGGGT

TTAATATCACCCCAATAAAATATTTATATAAATAAATAAAATTTAATTTCTAATTTTTATAAACTCAGTAAATATAAAGATTTATAGGGT

TTAATATCACCCCAATAAAATATTTATATAAATAAATAAAATTTAATTTCTAATTTTTATAAACTCAGTAAATATAAAGATTTATAGGGT

TTAATATCACCCCAATAAAATATTTATATAAATAAATAAAATTTAATTTCTAATTTTTATAAACTCAGTAAATATAAAGATTTATAGGGT

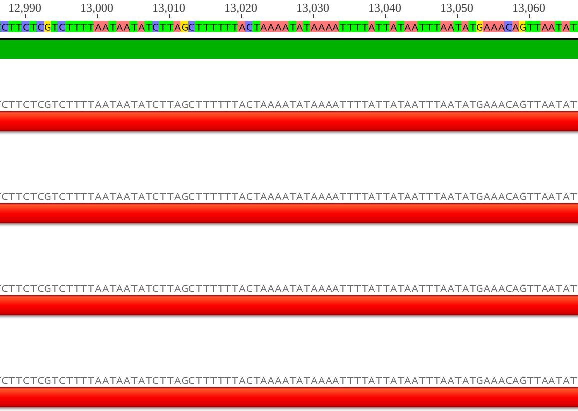

12,990
13,000
13,010
13,020
13,030
13,040
13,050
13,060
CTTCTCGTCTTTTAATAATATCTTAGCTTTTTTACTAAAATATAAAATTTTATTATAATTTAATATGAAACAGTTAATAT
CTTCTCGTCTTTTAATAATATCTTAGCTTTTTTACTAAAATATAAAATTTTATTATAATTTAATATGAAACAGTTAATAT
CTTCTCGTCTTTTAATAATATCTTAGCTTTTTTACTAAAATATAAAATTTTATTATAATTTAATATGAAACAGTTAATAT
CTTCTCGTCTTTTAATAATATCTTAGCTTTTTTACTAAAATATAAAATTTTATTATAATTTAATATGAAACAGTTAATAT
CTTCTCGTCTTTTAATAATATCTTAGCTTTTTTACTAAAATATAAAATTTTATTATAATTTAATATGAAACAGTTAATAT

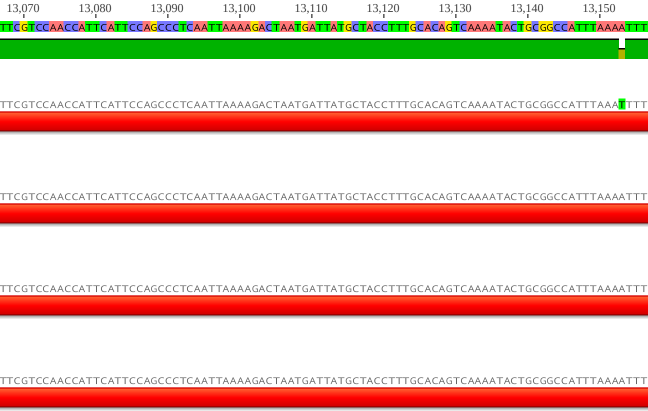
13,070
13,080
13,090
13,100
13,110
13,120
13,130
13,140
13,150
TTCGTCCAACCATTCATTCCAGCCCTCAATTAAAAGACTAATGATTATGCTACCTTTGCACAGTCAAAATACTGCGGCCATTTAAAATTT
TTCGTCCAACCATTCATTCCAGCCCTCAATTAAAAGACTAATGATTATGCTACCTTTGCACAGTCAAAATACTGCGGCCATTTAAATTTT
TTCGTCCAACCATTCATTCCAGCCCTCAATTAAAAGACTAATGATTATGCTACCTTTGCACAGTCAAAATACTGCGGCCATTTAAAATTT
TTCGTCCAACCATTCATTCCAGCCCTCAATTAAAAGACTAATGATTATGCTACCTTTGCACAGTCAAAATACTGCGGCCATTTAAAATTT
TTCGTCCAACCATTCATTCCAGCCCTCAATTAAAAGACTAATGATTATGCTACCTTTGCACAGTCAAAATACTGCGGCCATTTAAAATTT

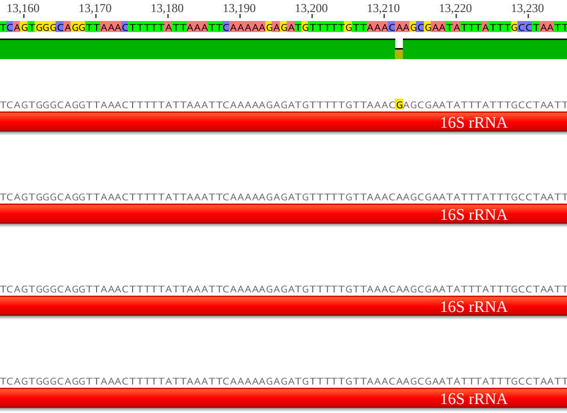

13,160
13,170
13,180
13,190
13,200
13,210
13,220
13,230
TCAGTGGGCAGGTTAAACTTTTTATTAAATTCAAAAAGAGATGTTTTTGTTAAACAAGCGAATATTTATTTGCCTAATT
TCAGTGGGCAGGTTAAACTTTTTATTAAATTCAAAAAGAGATGTTTTTGTTAAACGAGCGAATATTTATTTGCCTAATT
16S rRNA
TCAGTGGGCAGGTTAAACTTTTTATTAAATTCAAAAAGAGATGTTTTTGTTAAACAAGCGAATATTTATTTGCCTAATT
16S rRNA
TCAGTGGGCAGGTTAAACTTTTTATTAAATTCAAAAAGAGATGTTTTTGTTAAACAAGCGAATATTTATTTGCCTAATT
16S rRNA
TCAGTGGGCAGGTTAAACTTTTTATTAAATTCAAAAAGAGATGTTTTTGTTAAACAAGCGAATATTTATTTGCCTAATT
16S rRNA

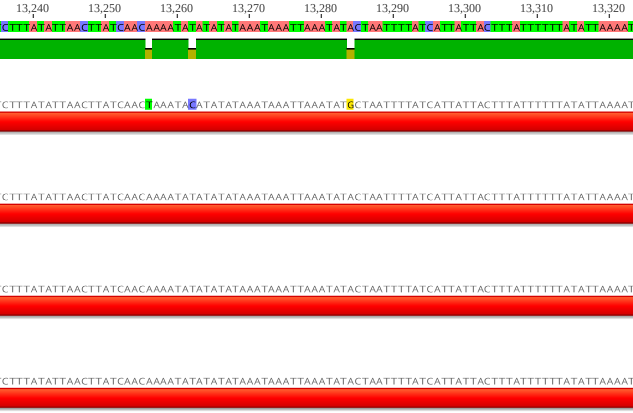
13,240
13,250
13,260
13,270
13,280
13,290
13,300
13,310
13,320
CTTTATATTAACTTATCAACAAAATATATATATAAATAAATTAAATATACTAATTTTATCATTATTACTTTATTTTTTATATTAAAAT
TCTTTATATTAACTTATCAACTAAATACATATATAAATAAATTAAATATGCTAATTTTATCATTATTACTTTATTTTTTATATTAAAAT
TCTTTATATTAACTTATCAACAAAATATATATATAAATAAATTAAATATACTAATTTTATCATTATTACTTTATTTTTTATATTAAAAT
TCTTTATATTAACTTATCAACAAAATATATATATAAATAAATTAAATATACTAATTTTATCATTATTACTTTATTTTTTATATTAAAAT
TCTTTATATTAACTTATCAACAAAATATATATATAAATAAATTAAATATACTAATTTTATCATTATTACTTTATTTTTTATATTAAAAT

13,330 13,340 13,350 13,360 13,370 13,380 13,390 13,400

TAAATATTTTAATAAATTAATTAAAATAAAATAAATAATTTTTTAATAATATTAATTATAACACTATCACTAATAATAACTACT

TAAATATTTTAATAAATTAATTAAAATAAAATAAATAATTTTTTAATAATATTAATTATAACACTATCACTAATAATAACTACT

TAAATATTTTAATAAATTAATTAAAATAAAATAAATAATTTTTTAATAATATTAATTATAACACTATCACTAATAATAACTACT

TAAATATTTTAATAAATTAATTAAAATAAAATAAATAATTTTTTAATAATATTAATTATAACACTATCACTAATAATAACTACT

TAAATATTTTAATAAATTAATTAAAATAAAATAAATAATTTTTTAATAATATTAATTATAACACTATCACTAATAATAACTACT

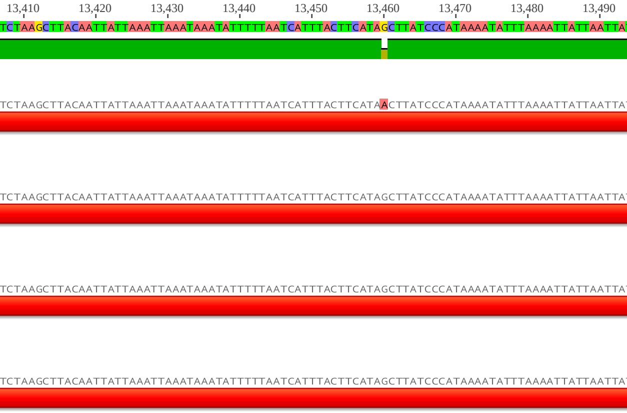

13,410
13,420
13,430
13,440
13,450
13,460
13,470
13,480
13,490
TCTAAGCTTACAATTATTAAATTAAATAAATATTTTTAATCATTTACTTCATAGCTTATCCCATAAAATATTTAAAATTATTAATTAT
TCTAAGCTTACAATTATTAAATTAAATAAATATTTTTAATCATTTACTTCATAACTTATCCCATAAAATATTTAAAATTATTAATTAT
TCTAAGCTTACAATTATTAAATTAAATAAATATTTTTAATCATTTACTTCATAGCTTATCCCATAAAATATTTAAAATTATTAATTAT
TCTAAGCTTACAATTATTAAATTAAATAAATATTTTTAATCATTTACTTCATAGCTTATCCCATAAAATATTTAAAATTATTAATTAT
TCTAAGCTTACAATTATTAAATTAAATAAATATTTTTAATCATTTACTTCATAGCTTATCCCATAAAATATTTAAAATTATTAATTAT

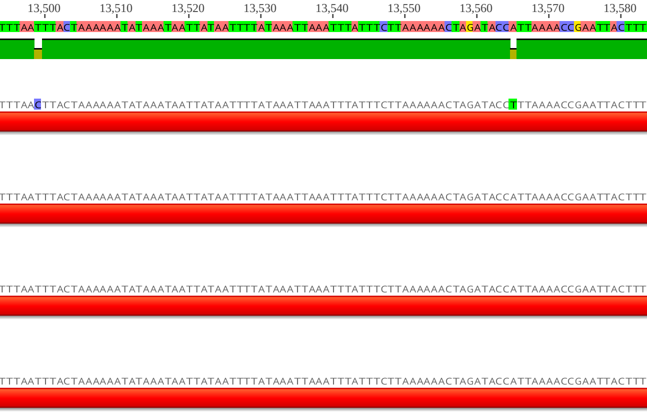

13,500
13,510
13,520
13,530
13,540
13,550
13,560
13,570
13,580
TTTAATTTACTAAAAAATATAAATAATTATAATTTTATAAATTAAATTTATTTCTTAAAAAACTAGATACCATTAAAACCGAATTACTTT
TTTAACTTACTAAAAAATATAAATAATTATAATTTTATAAATTAAATTTATTTCTTAAAAAACTAGATACCTTTAAAACCGAATTACTTT
TTTAATTTACTAAAAAATATAAATAATTATAATTTTATAAATTAAATTTATTTCTTAAAAAACTAGATACCATTAAAACCGAATTACTTT
TTTAATTTACTAAAAAATATAAATAATTATAATTTTATAAATTAAATTTATTTCTTAAAAAACTAGATACCATTAAAACCGAATTACTTT
TTTAATTTACTAAAAAATATAAATAATTATAATTTTATAAATTAAATTTATTTCTTAAAAAACTAGATACCATTAAAACCGAATTACTTT

13,590 13,600 13,610 13,620 13,630 13,640 13,650 13,660

TCACTTCCAATATATTATTAAAAATAATTTTATTACATTAAATTTCATAATATATTAACTCTTTTAAAATCGAAAAATTT

TCACTTCCAATATATTATTAAAAATAATTTTATTACATTAAATTTCATAATATATTAACTCTTTTAAAATCGAAAAATTT

TCACTTCCAATATATTATTAAAAATAATTTTATTACATTAAATTTCATAATATATTAACTCTTTTAAAATCGAAAAATTT

TCACTTCCAATATATTATTAAAAATAATTTTATTACATTAAATTTCATAATATATTAACTCTTTTAAAATCGAAAAATTT

TCACTTCCAATATATTATTAAAAATAATTTTATTACATTAAATTTCATAATATATTAACTCTTTTAAAATCGAAAAATTT

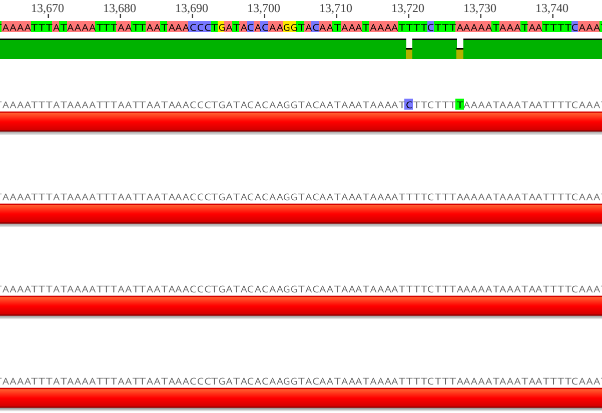
13,670
13,680
13,690
13,700
13,710
13,720
13,730
13,740
AAAATTTATAAAATTTAATTAATAAACCCTGATACACAAGGTACAATAAATAAAATTTTCTTTAAAAATAAATAATTTTCAAA
AAAATTTATAAAATTTAATTAATAAACCCTGATACACAAGGTACAATAAATAAAATCTTCTTTTAAAATAAATAATTTTCAAA
AAAATTTATAAAATTTAATTAATAAACCCTGATACACAAGGTACAATAAATAAAATTTTCTTTAAAAATAAATAATTTTCAAA
AAAATTTATAAAATTTAATTAATAAACCCTGATACACAAGGTACAATAAATAAAATTTTCTTTAAAAATAAATAATTTTCAAA
AAAATTTATAAAATTTAATTAATAAACCCTGATACACAAGGTACAATAAATAAAATTTTCTTTAAAAATAAATAATTTTCAAA

13,750 13,760 13,770 13,780 13,790 13,800 13,810 13,820 13,830

TTATTTCAATATTCTTTCACAATACTAATTCACTATAATAAAATATATTTTTTCTATTAACATACTAAAAAAACTAATAAATATTTT

TTATTTCAATATTCTTTCACAATACTAATTCACTATAATAAAATATATTTTTTCTATTAACATACTAAAAAAACTAATAAATATTTT

TTATTTCAATATTCTTTCACAATACTAATTCACTATAATAAAATATATTTTTTCTATTAACATACTAAAAAAACTAATAAATATTTT

TTATTTCAATATTCTTTCACAATACTAATTCACTATAATAAAATATATTTTTTCTATTAACATACTAAAAAAACTAATAAATATTTT

TTATTTCAATATTCTTTCACAATACTAATTCACTATAATAAAATATATTTTTTCTATTAACATACTAAAAAAACTAATAAATATTTT

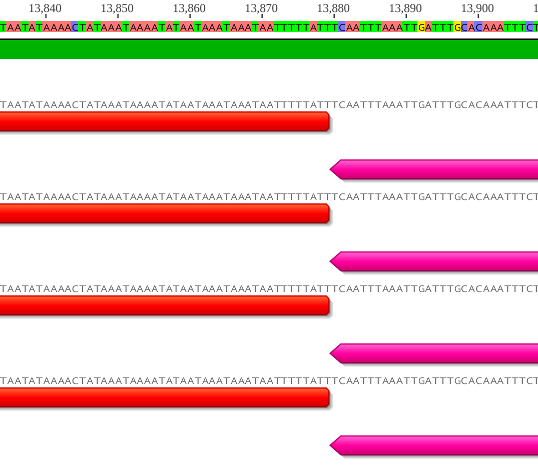
13,840
13,850
13,860
13,870
13,880
13,890
13,900
TAATATAAAACTATAAATAAAATATAATAAATAAATAATTTTTATTTCAATTTAAATTGATTTGCACAAATTTCT
TAATATAAAACTATAAATAAAATATAATAAATAAATAATTTTTATTTCAATTTAAATTGATTTGCACAAATTTCT
TAATATAAAACTATAAATAAAATATAATAAATAAATAATTTTTATTTCAATTTAAATTGATTTGCACAAATTTCT
TAATATAAAACTATAAATAAAATATAATAAATAAATAATTTTTATTTCAATTTAAATTGATTTGCACAAATTTCT
TAATATAAAACTATAAATAAAATATAATAAATAAATAATTTTTATTTCAATTTAAATTGATTTGCACAAATTTCT

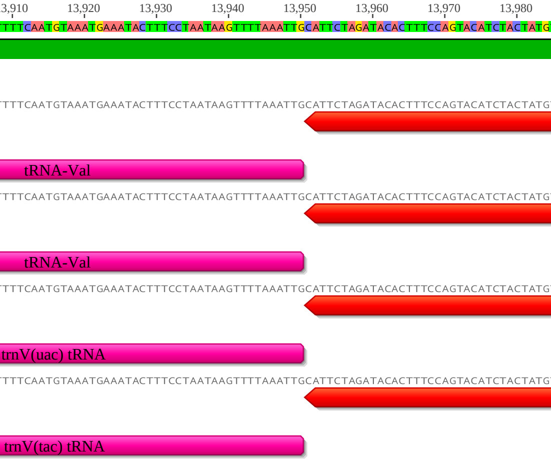

13,910
13,920
13,930
13,940
13,950
13,960
13,970
13,980
TTTTCAATGTAAATGAAATACTTTCCTAATAAGTTTTAAATTGCATTCTAGATACACTTTCCAGTACATCTACTATG
TTTTCAATGTAAATGAAATACTTTCCTAATAAGTTTTAAATTGCATTCTAGATACACTTTCCAGTACATCTACTATG
tRNA-Val
TTTTCAATGTAAATGAAATACTTTCCTAATAAGTTTTAAATTGCATTCTAGATACACTTTCCAGTACATCTACTATG
tRNA-Val
TTTTCAATGTAAATGAAATACTTTCCTAATAAGTTTTAAATTGCATTCTAGATACACTTTCCAGTACATCTACTATG
trnV(uac) tRNA
TTTTCAATGTAAATGAAATACTTTCCTAATAAGTTTTAAATTGCATTCTAGATACACTTTCCAGTACATCTACTATG
trnV(tac) tRNA

13,990 14,000 14,010 14,020 14,030 14,040 14,050 14,060

TTACGACTTATCTTACCTTAATAATAAGAGTGACGGGCGATATGTACATATTTTAGAGCCAAAATCAAATTATTTATCTTTA

TTACGACTTATCTTACCTTAATAATAAGAGTGACGGGCGATATGTACATATTTTAGAGCCAAAATCAAATTATTTATCTTTA

TTACGACTTATCTTACCTTAATAATAAGAGTGACGGGCGATATGTACATATTTTAGAGCCAAAATCAAATTATTTATCTTTA

TTACGACTTATCTTACCTTAATAATAAGAGTGACGGGCGATATGTACATATTTTAGAGCCAAAATCAAATTATTTATCTTTA

TTACGACTTATCTTACCTTAATAATAAGAGTGACGGGCGATATGTACATATTTTAGAGCCAAAATCAAATTATTTATCTTTA

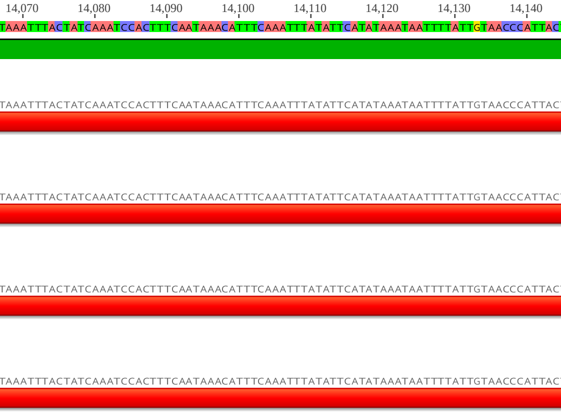
14,070
14,080
14,090
14,100
14,110
14,120
14,130
14,140
TAAATTTACTATCAAATCCACTTTCAATAAACATTTCAAATTTATATTCATATAAATAATTTTATTGTAACCCATTAC
TAAATTTACTATCAAATCCACTTTCAATAAACATTTCAAATTTATATTCATATAAATAATTTTATTGTAACCCATTAC
TAAATTTACTATCAAATCCACTTTCAATAAACATTTCAAATTTATATTCATATAAATAATTTTATTGTAACCCATTAC
TAAATTTACTATCAAATCCACTTTCAATAAACATTTCAAATTTATATTCATATAAATAATTTTATTGTAACCCATTAC
TAAATTTACTATCAAATCCACTTTCAATAAACATTTCAAATTTATATTCATATAAATAATTTTATTGTAACCCATTAC

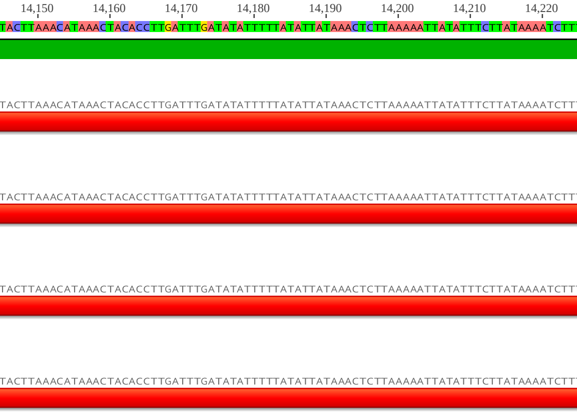
14,150
14,160
14,170
14,180
14,190
14,200
14,210
14,220
TACTTAAACATAAACTACACCTTGATTTGATATATTTTTATATTATAAACTCTTAAAAATTATATTTCTTATAAAATCTT
TACTTAAACATAAACTACACCTTGATTTGATATATTTTTATATTATAAACTCTTAAAAATTATATTTCTTATAAAATCTT
TACTTAAACATAAACTACACCTTGATTTGATATATTTTTATATTATAAACTCTTAAAAATTATATTTCTTATAAAATCTT
TACTTAAACATAAACTACACCTTGATTTGATATATTTTTATATTATAAACTCTTAAAAATTATATTTCTTATAAAATCTT
TACTTAAACATAAACTACACCTTGATTTGATATATTTTTATATTATAAACTCTTAAAAATTATATTTCTTATAAAATCTT

14,230 14,240 14,250 14,260 14,270 14,280 14,290 14,300

TAAATAACAACGGTATATAAATTGAATACAAGTTCAAGTAAGGTCCAACGTGGATTATCAATTACAGAACAGGTTCCTCT

TAAATAACAACGGTATATAAATTGAATACAAGTTCAAGTAAGGTCCAACGTGGATTATCAATTACAGAACAGGTTCCTCT

TAAATAACAACGGTATATAAATTGAATACAAGTTCAAGTAAGGTCCAACGTGGATTATCAATTACAGAACAGGTTCCTCT

TAAATAACAACGGTATATAAATTGAATACAAGTTCAAGTAAGGTCCAACGTGGATTATCAATTACAGAACAGGTTCCTCT

TAAATAACAACGGTATATAAATTGAATACAAGTTCAAGTAAGGTCCAACGTGGATTATCAATTACAGAACAGGTTCCTCT

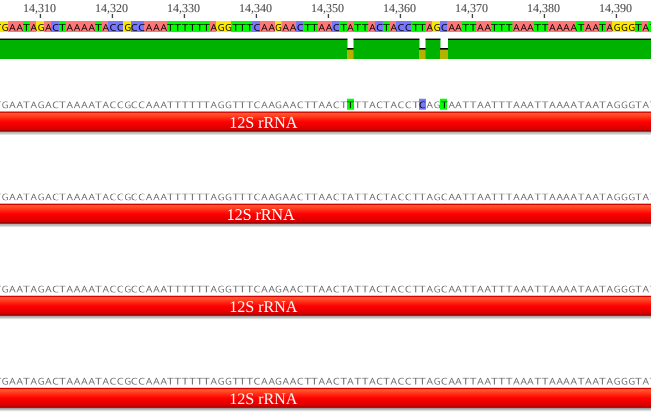

14,310
14,320
14,330
14,340
14,350
14,360
14,370
14,380
14,390
GAATAGACTAAAATACCGCCAAATTTTTTAGGTTTCAAGAACTTAACTATTACTACCTTAGCAATTAATTTAAATTAAAATAATAGGGTA
GAATAGACTAAAATACCGCCAAATTTTTTAGGTTTCAAGAACTTAACTTTTACTACCTCAGTAATTAATTTAAATTAAAATAATAGGGTA
12S rRNA
GAATAGACTAAAATACCGCCAAATTTTTTAGGTTTCAAGAACTTAACTATTACTACCTTAGCAATTAATTTAAATTAAAATAATAGGGTA
12S rRNA
GAATAGACTAAAATACCGCCAAATTTTTTAGGTTTCAAGAACTTAACTATTACTACCTTAGCAATTAATTTAAATTAAAATAATAGGGTA
12S rRNA
GAATAGACTAAAATACCGCCAAATTTTTTAGGTTTCAAGAACTTAACTATTACTACCTTAGCAATTAATTTAAATTAAAATAATAGGGTA
12S rRNA

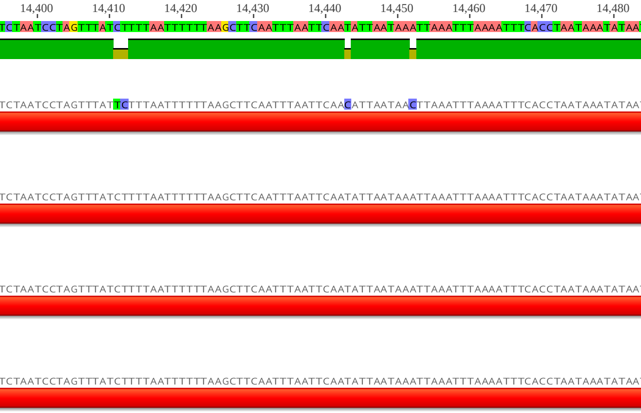
14,400 14,410 14,420 14,430 14,440 14,450 14,460 14,470 14,480
TCTAATCCTAGTTTATCTTTTAATTTTTTAAGCTTCAATTTAATTCAATATTAATAAATTAAATTTAAAATTTCACCTAATAAATATAAT
TCTAATCCTAGTTTATTCTTTAATTTTTTAAGCTTCAATTTAATTCAACATTAATAACTTAAATTTAAAATTTCACCTAATAAATATAAT
TCTAATCCTAGTTTATCTTTTAATTTTTTAAGCTTCAATTTAATTCAATATTAATAAATTAAATTTAAAATTTCACCTAATAAATATAAT
TCTAATCCTAGTTTATCTTTTAATTTTTTAAGCTTCAATTTAATTCAATATTAATAAATTAAATTTAAAATTTCACCTAATAAATATAAT
TCTAATCCTAGTTTATCTTTTAATTTTTTAAGCTTCAATTTAATTCAATATTAATAAATTAAATTTAAAATTTCACCTAATAAATATAAT

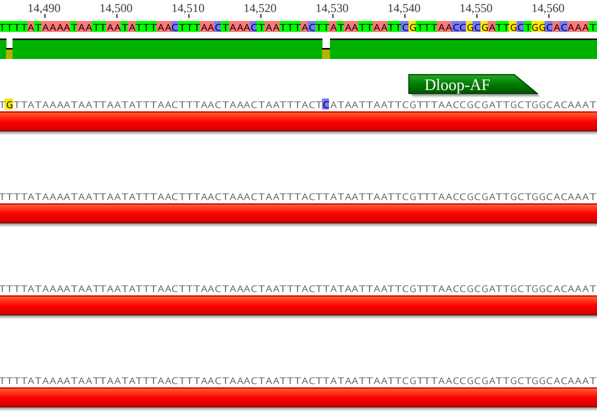
14,490
14,500
14,510
14,520
14,530
14,540
14,550
14,560
TTTTATAAAATAATTAATATTTAACTTTAACTAAACTAATTTACTTATAATTAATTCGTTTAACCGCGATTGCTGGCACAAAT
Dloop-AF
TGTTATAAAATAATTAATATTTAACTTTAACTAAACTAATTTACTCATAATTAATTCGTTTAACCGCGATTGCTGGCACAAAT
TTTTATAAAATAATTAATATTTAACTTTAACTAAACTAATTTACTTATAATTAATTCGTTTAACCGCGATTGCTGGCACAAAT
TTTTATAAAATAATTAATATTTAACTTTAACTAAACTAATTTACTTATAATTAATTCGTTTAACCGCGATTGCTGGCACAAAT
TTTTATAAAATAATTAATATTTAACTTTAACTAAACTAATTTACTTATAATTAATTCGTTTAACCGCGATTGCTGGCACAAAT

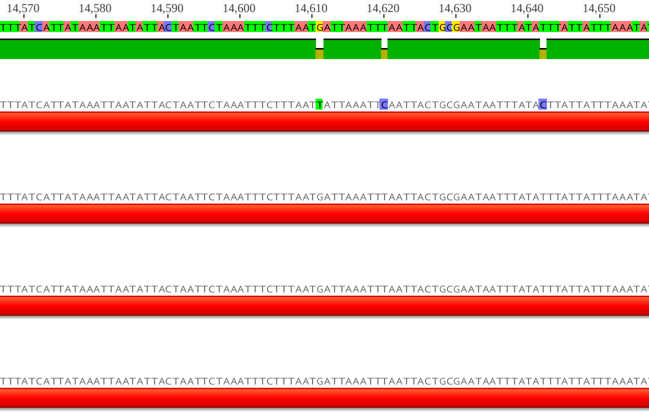
14,570 14,580 14,590 14,600 14,610 14,620 14,630 14,640 14,650
TTTATCATTATAAATTAATATTACTAATTCTAAATTTCTTTAATGATTAAATTTAATTACTGCGAATAATTTATATTTATTATTTAAATA
TTTATCATTATAAATTAATATTACTAATTCTAAATTTCTTTAATTATTAAATTCAATTACTGCGAATAATTTATACTTATTATTTAAATA
TTTATCATTATAAATTAATATTACTAATTCTAAATTTCTTTAATGATTAAATTTAATTACTGCGAATAATTTATATTTATTATTTAAATA
TTTATCATTATAAATTAATATTACTAATTCTAAATTTCTTTAATGATTAAATTTAATTACTGCGAATAATTTATATTTATTATTTAAATA
TTTATCATTATAAATTAATATTACTAATTCTAAATTTCTTTAATGATTAAATTTAATTACTGCGAATAATTTATATTTATTATTTAAATA

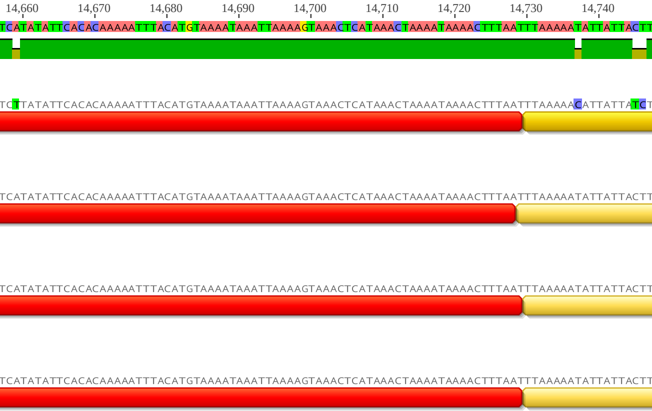
14,660
14,670
14,680
14,690
14,700
14,710
14,720
14,730
14,740
TCATATATTCACACAAAAATTTACATGTAAAATAAATTAAAAGTAAACTCATAAACTAAAATAAAACTTTAATTTAAAAATATTATTACTT
TCTTATATTCACACAAAAATTTACATGTAAAATAAATTAAAAGTAAACTCATAAACTAAAATAAAACTTTAATTTAAAAACATTATTATCT
TCATATATTCACACAAAAATTTACATGTAAAATAAATTAAAAGTAAACTCATAAACTAAAATAAAACTTTAATTTAAAAATATTATTACTT
TCATATATTCACACAAAAATTTACATGTAAAATAAATTAAAAGTAAACTCATAAACTAAAATAAAACTTTAATTTAAAAATATTATTACTT
TCATATATTCACACAAAAATTTACATGTAAAATAAATTAAAAGTAAACTCATAAACTAAAATAAAACTTTAATTTAAAAATATTATTACTT

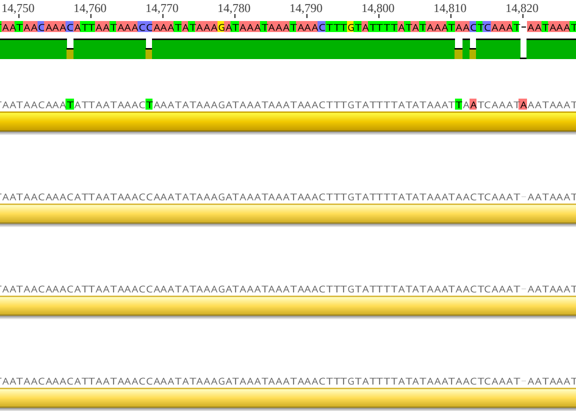
14,750
14,760
14,770
14,780
14,790
14,800
14,810
14,820
AATAACAAACATTAATAAACCAAATATAAAGATAAATAAATAAACTTTGTATTTTATATAAATAACTCAAAT-AATAAAT
AATAACAAATATTAATAAACTAAATATAAAGATAAATAAATAAACTTTGTATTTTATATAAATTAATCAAATAAATAAAT
AATAACAAACATTAATAAACCAAATATAAAGATAAATAAATAAACTTTGTATTTTATATAAATAACTCAAAT-AATAAAT
AATAACAAACATTAATAAACCAAATATAAAGATAAATAAATAAACTTTGTATTTTATATAAATAACTCAAAT-AATAAAT
AATAACAAACATTAATAAACCAAATATAAAGATAAATAAATAAACTTTGTATTTTATATAAATAACTCAAAT-AATAAAT

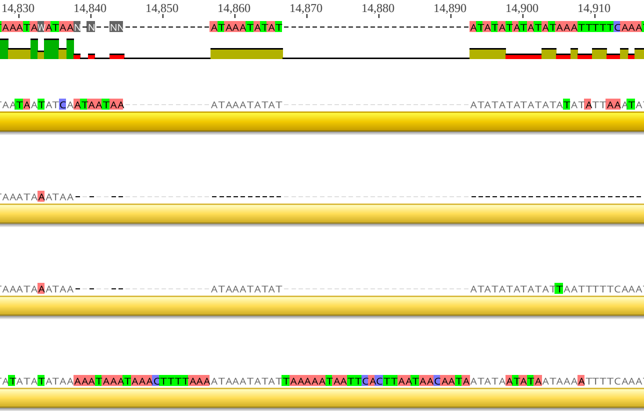
14,830
14,840
14,850
14,860
14,870
14,880
14,890
14,900
14,910
AAATAWATAAN N NN ATAAATATAT ATATATATATATATAAATTTTTCAAA
AATAATATCAATAATAA ATAAATATAT ATATATATATATATATATTAAATAT
AAATAAATAA
AAATAAATAA ATAAATATAT ATATATATATATTAATTTTTCAAA
TATATTATAAAAATAAATAAACTTTTAAAATAAATATATTAAAAATAATTCACTTAATAACAATAATATAAATATAATAAAATTTTCAAA

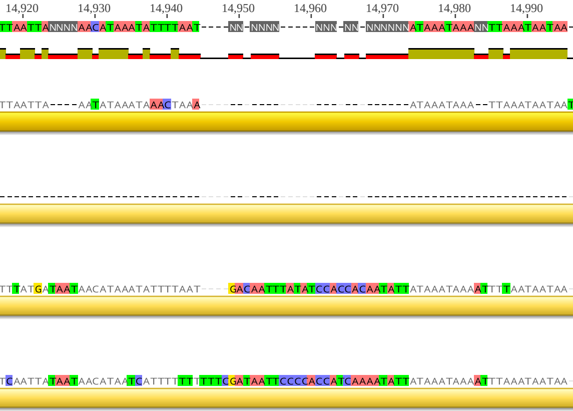

14,920
14,930
14,940
14,950
14,960
14,970
14,980
14,990
TTAATTANNNNAACATAAATATTTTAAT----NN-NNNN-----NNN-NN-NNNNNNATAAATAAANNTTAAATAATAA-
TTAATTA----AATATAAATAAACTAAA--------------------ATAAATAAA--TTAAATAATAAT
TTTATGATAATAACATAAATATTTTAAT----GACAATTTATATCCACCACAATATTATAAATAAAATTTTAATAATAA-
TCAATTATAATAACATAATCATTTTTTTTTTCGATAATTCCCCACCATCAAAATATTATAAATAAAATTTAAATAATAA-

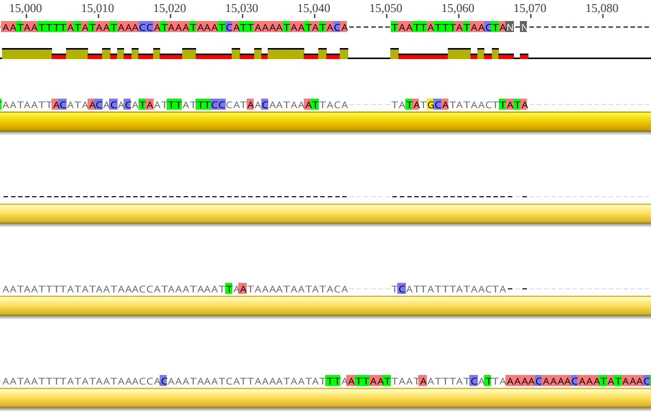
15,000
15,010
15,020
15,030
15,040
15,050
15,060
15,070
15,080

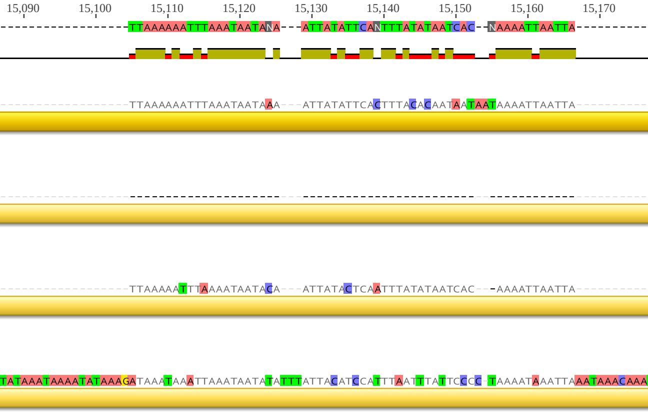
15,090
15,100
15,110
15,120
15,130
15,140
15,150
15,160
15,170

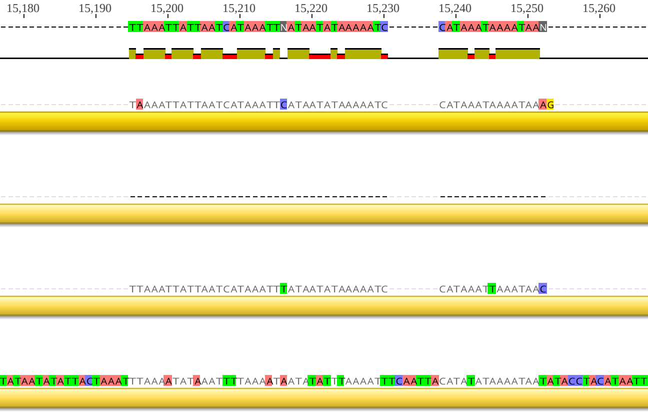
15,180
15,190
15,200
15,210
15,220
15,230
15,240
15,250
15,260
TTAAATTATTAATCATAAATTNATAATATAAAAATC
CATAAATAAAATAAN
TAAAATTATTAATCATAAATTCATAATATAAAAATC
CATAAATAAAATAAAG
TTAAATTATTAATCATAAATTTATAATATAAAAATC
CATAAATTAAATAAC

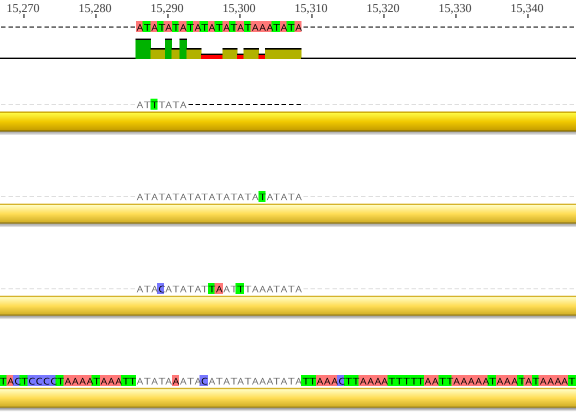
15,270
15,280
15,290
15,300
15,310
15,320
15,330
15,340
ATATATATATATATATAAATATA
ATTTATA
ATATATATATATATATATATATA
ATACATATATTAATTTAAATATA
TACTCCCCTAAAATAAATTATATAAATACATATATAAATATATTAAACTTAAAATTTTTAATTAAAAATAAATATAAAAT

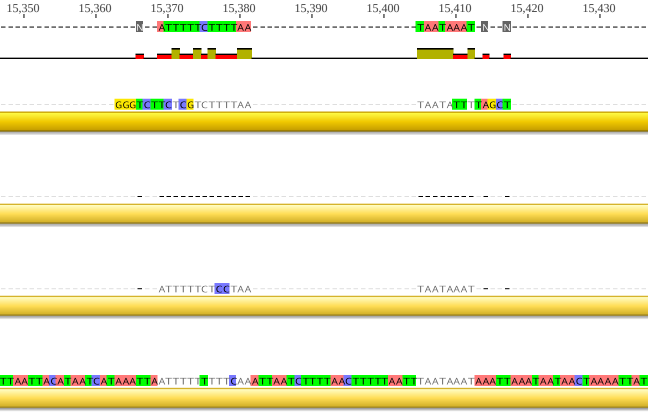
15,350
15,360
15,370
15,380
15,390
15,400
15,410
15,420
15,430
N
ATTTTTCTTTTAA
TAATAAAT
N
N
GGGTCTTCTCGTCTTTTAA
TAATATTTTTAGCT
ATTTTTCTCCTAA
TAATAAAT
TTAATTACATAATCATAAATTAATTTTTTTTTTCAAATTAATCTTTTAACTTTTTAATTTAATAAATAAATTAAATAATAACTAAAATTAT

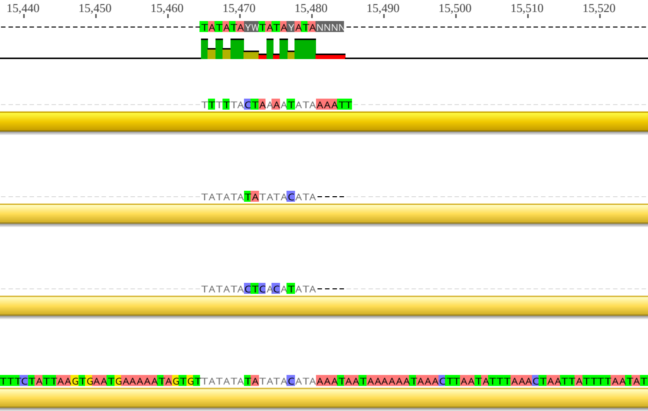
15,440
15,450
15,460
15,470
15,480
15,490
15,500
15,510
15,520
TATATAYWTATAYATANNNN
TTTTTACTAAAATATAAAATT
TATATATATATACATA
TATATACTCACATATA
TTTCTATTAAGTGAATGAAAAATAGTGTTATATATATATACATAAAATAATAAAAAATAAACTTAATATTTAAACTAATTATTTTAATAT

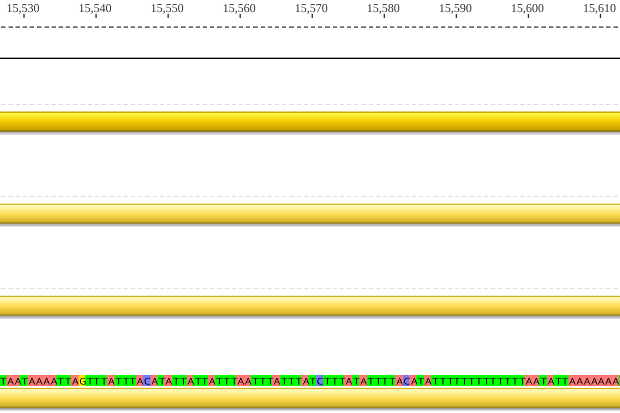

15,530
15,540
15,550
15,560
15,570
15,580
15,590
15,600
15,610
TAATAAAATTAGTTTATTTACATATTATTATTTAATTTATTTATCTTTATATTTTACATATTTTTTTTTTTTTTAATATTAAAAAAA

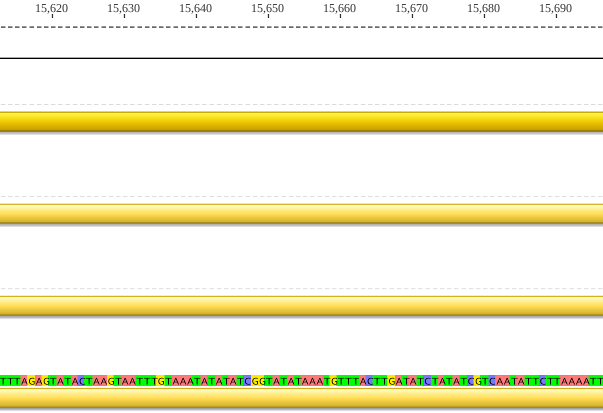
15,620
15,630
15,640
15,650
15,660
15,670
15,680
15,690
TTTAGAGTATACTAAGTAATTTGTAAATATATATCGGTATATAAATGTTTACTTGATATCTATATCGTCAATATTCTTAAAATT

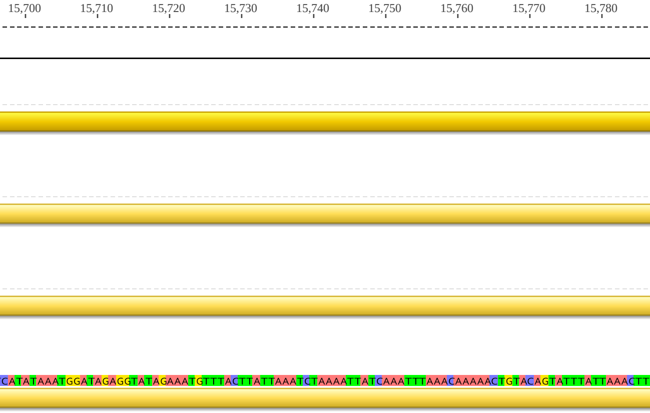
15,700 15,710 15,720 15,730 15,740 15,750 15,760 15,770 15,780
CATATAAATGGATAGAGGTATAGAAATGTTTACTTATTAAATCTAAAATTATCAAATTTAAACAAAAACTGTACAGTATTTATTAAACTT

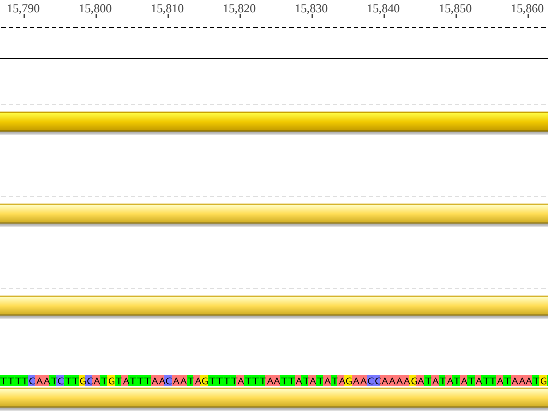
15,790
15,800
15,810
15,820
15,830
15,840
15,850
15,860
TTTTCAATCTTGCATGTATTTAACAATAGTTTTATTTAATTATATATAGAACCAAAAGATATATATATTATAAATG

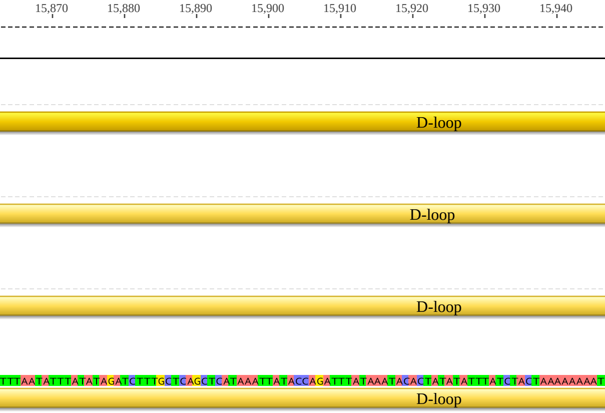

15,870
15,880
15,890
15,900
15,910
15,920
15,930
15,940
D-loop
D-loop
D-loop
TTTAATATTTATATAGATCTTTGCTCAGCTCATAAATTATACCAGATTTATAAATACACTATATATTTATCTACTAAAAAAAAT
D-loop

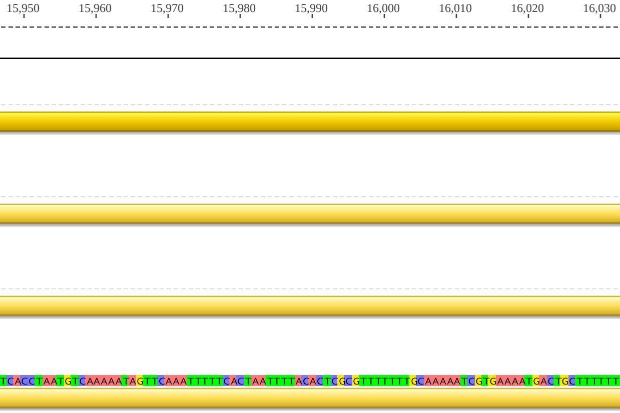
15,950
15,960
15,970
15,980
15,990
16,000
16,010
16,020
16,030
TCACCTAATGTCAAAAATAGTTCAAATTTTTCACTAATTTTACACTCGCGTTTTTTTGCAAAAATCGTGAAAATGACTGCTTTTTT

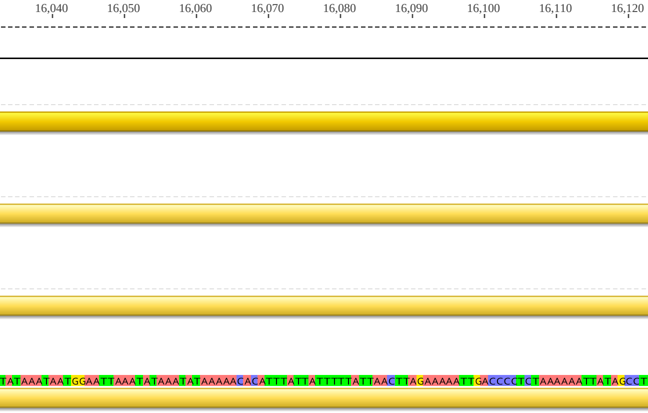
16,040
16,050
16,060
16,070
16,080
16,090
16,100
16,110
16,120
TATAAATAATGGAATTAAATATAAATATAAAAACACATTTATTATTTTTATTAACTTAGAAAAATTGACCCCTCTAAAAAATTATAGCCT

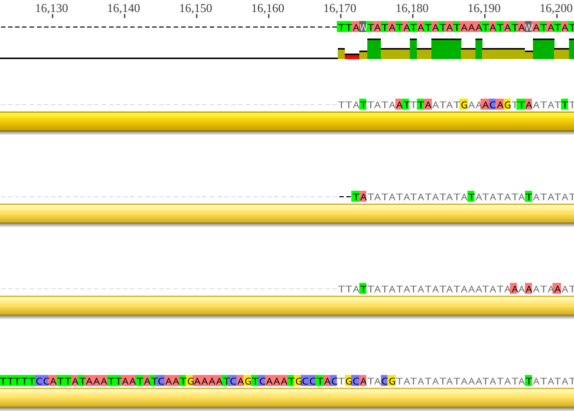
16,130
16,140
16,150
16,160
16,170
16,180
16,190
16,200
TTAWTATATATATATATAAATATATAWATATAT
TTATTATAATTTAATATGAAACAGTTAATATTT
TATATATATATATATATATATATATATATAT
TTATTATATATATATATAAATATAAAAATAAAT
TTTTTCCATTATAAATTAATATCAATGAAAATCAGTCAAATGCCTACTGCATACGTATATATATAAATATATATATAT

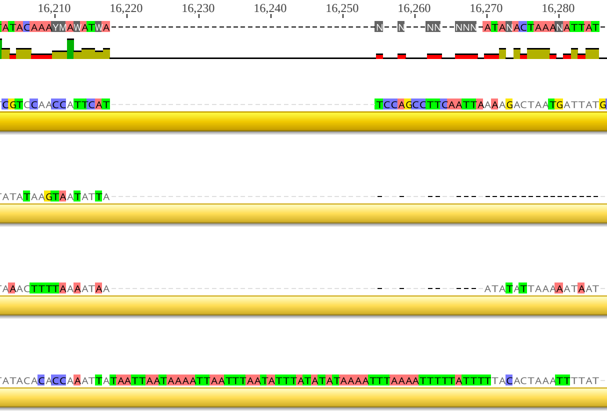
16,210
16,220
16,230
16,240
16,250
16,260
16,270
16,280

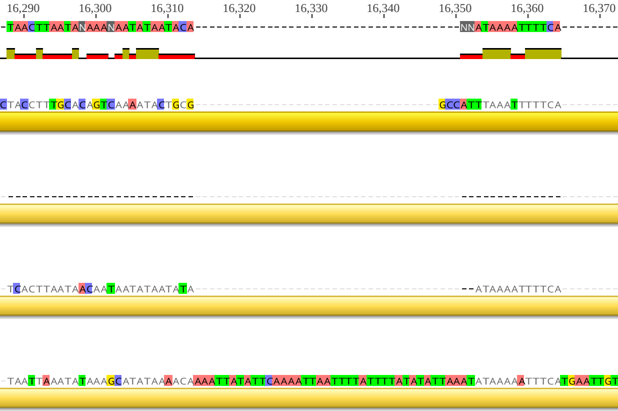
16,290
16,300
16,310
16,320
16,330
16,340
16,350
16,360
16,370
TAACTTAATANAAANAATATAATACA
NNATAAAATTTTCA
CTACCTTTGCACAGTCAAAATACTGCG
GCCATTTAAATTTTTCA
TCACTTAATAACAATAATATAATATA
ATAAAATTTTCA
TAATTAAATATAAAGCATATAAACAAAATTATATTCAAAATTAATTTTATTTTATATATTAAATATAAAAATTTCATGAATTGT

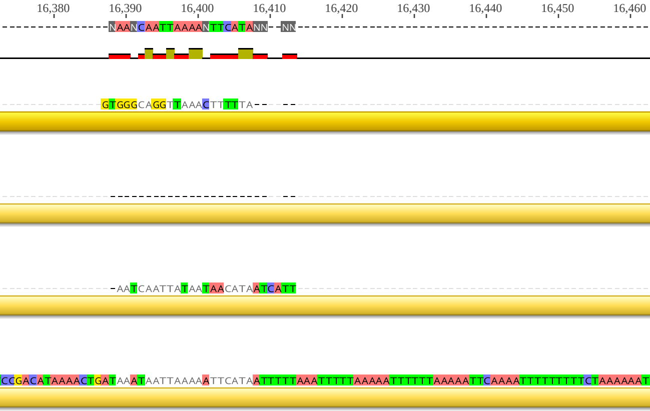
16,380
16,390
16,400
16,410
16,420
16,430
16,440
16,450
16,460
NAANCAATTAAAANTTCATANN NN
GTGGGCAGGTTAAACTTTTTA
AATCAATTATAATAACATAATCATT
CCGACATAAAACTGATAAATAATTAAAAATTCATAATTTTTAAATTTTTAAAAATTTTTTAAAAATTCAAAATTTTTTTTTCTAAAAAAT

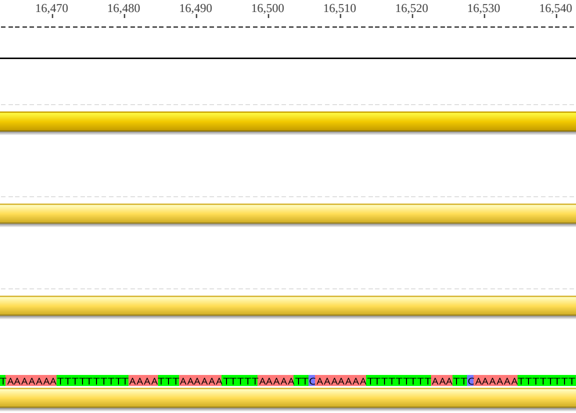
16,470
16,480
16,490
16,500
16,510
16,520
16,530
16,540
TAAAAAAATTTTTTTTTTAAAATTTAAAAAATTTTTAAAAATTCAAAAAAATTTTTTTTTAAATTCAAAAAATTTTTTTT

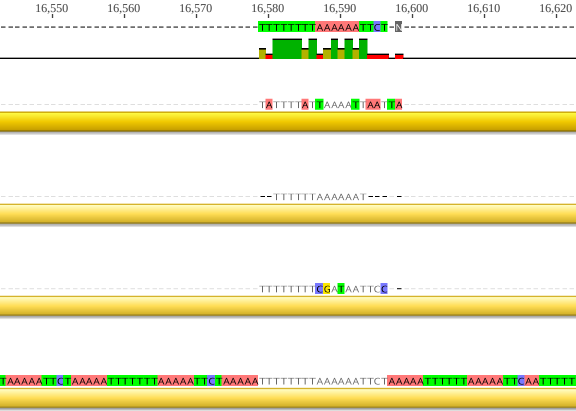
16,550
16,560
16,570
16,580
16,590
16,600
16,610
16,620
TTTTTTTTAAAAAATTCT
N
TATTTTATTAAAATTAATTA
TTTTTTAAAAAAT
TTTTTTTTCGATAATTCC
TAAAAATTCTAAAAATTTTTTTAAAAATTCTAAAAATTTTTTTTAAAAAATTCTAAAAATTTTTTAAAAATTCAATTTTT

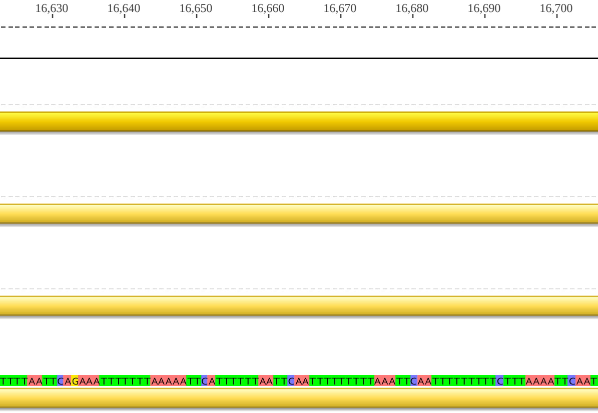

16,630
16,640
16,650
16,660
16,670
16,680
16,690
16,700
TTTTAATTCAGAAATTTTTTTAAAAATTCATTTTTTAATTCAATTTTTTTTTAAATTCAATTTTTTTTTCTTTAAAATTCAAT

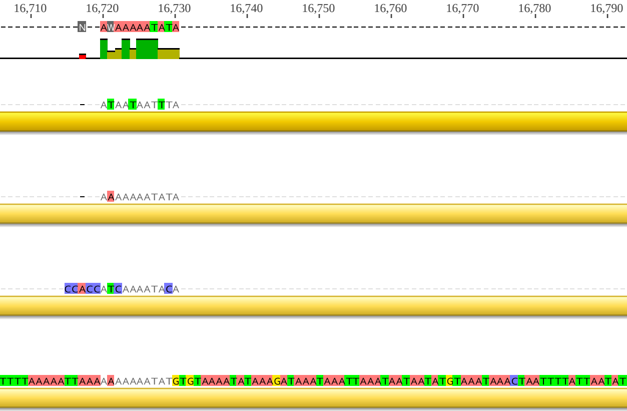
16,710
16,720
16,730
16,740
16,750
16,760
16,770
16,780
16,790
N
AWAAAAATATA
ATAATAATTTA
AAAAAAATATA
CCACCATCAAAATACA

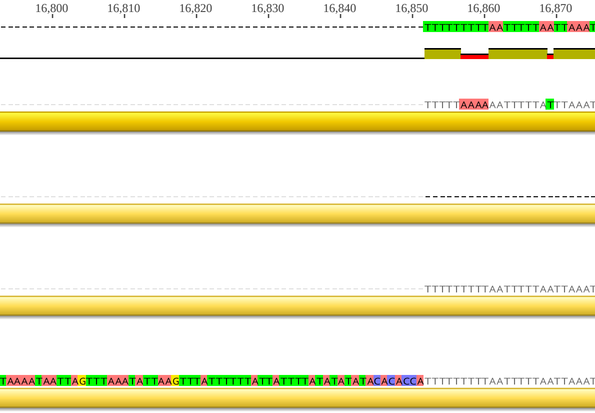
16,800
16,810
16,820
16,830
16,840
16,850
16,860
16,870
TTTTTTTTTAATTTTTAATTAAAT
TTTTTAAAAAATTTTTATTTAAAT
TTTTTTTTTAATTTTTAATTAAAT
TAAAATAATTAGTTTAAATATTAAGTTTATTTTTTTATTATTTTATATATATACACACCATTTTTTTTTAATTTTTAATTAAAT

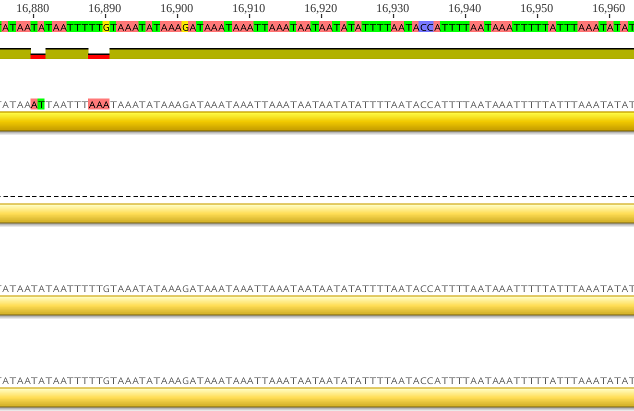
16,880 16,890 16,900 16,910 16,920 16,930 16,940 16,950 16,960
ATAATATAATTTTTGTAAATATAAAGATAAATAAATTAAATAATAATATATTTTAATACCATTTTAATAAATTTTTATTTAAATATAT
TATAAATTAATTTAAATAAATATAAAGATAAATAAATTAAATAATAATATATTTTAATACCATTTTAATAAATTTTTATTTAAATATAT
TATAATATAATTTTTGTAAATATAAAGATAAATAAATTAAATAATAATATATTTTAATACCATTTTAATAAATTTTTATTTAAATATAT
TATAATATAATTTTTGTAAATATAAAGATAAATAAATTAAATAATAATATATTTTAATACCATTTTAATAAATTTTTATTTAAATATAT

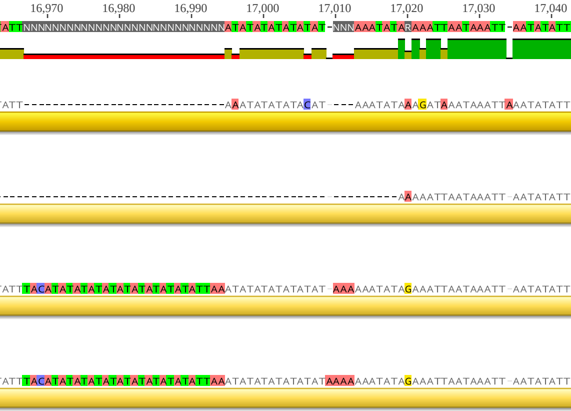

16,970
16,980
16,990
17,000
17,010
17,020
17,030
17,040

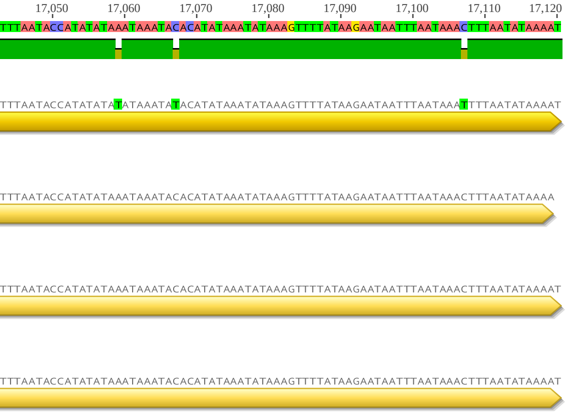

17,050
17,060
17,070
17,080
17,090
17,100
17,110
17,120
TTTAATACCATATATAAATAAATACACATATAAATATAAAGTTTTATAAGAATAATTTAATAAACTTTAATATAAAAT
TTTAATACCATATATATATAAATATACATATAAATATAAAGTTTTATAAGAATAATTTAATAAATTTTAATATAAAAT
TTTAATACCATATATAAATAAATACACATATAAATATAAAGTTTTATAAGAATAATTTAATAAACTTTAATATAAAA
TTTAATACCATATATAAATAAATACACATATAAATATAAAGTTTTATAAGAATAATTTAATAAACTTTAATATAAAAT
TTTAATACCATATATAAATAAATACACATATAAATATAAAGTTTTATAAGAATAATTTAATAAACTTTAATATAAAAT
